# Tejaas: reverse regression increases power for detecting *trans*-eQTLs

**DOI:** 10.1186/s13059-021-02361-8

**Published:** 2021-05-06

**Authors:** Saikat Banerjee, Franco L. Simonetti, Kira E. Detrois, Anubhav Kaphle, Raktim Mitra, Rahul Nagial, Johannes Söding

**Affiliations:** 1 Quantitative and Computational Biology, Max-Planck Institute for Biophysical Chemistry, Göttingen, 37077 Germany; 2 Georg-August University, Göttingen, 37075 Germany; 3 Indian Institute of Technology, Kanpur, India; 4 Campus-Institut Data Science (CIDAS), University of Göttingen, Göttingen, 37073 Germany; 5 Cluster of Excellence “Multiscale Bioimaging” (MBExC), University of Göttingen, Göttingen, 37075 Germany

## Abstract

*Trans*-acting expression quantitative trait loci (*trans*-eQTLs) account for ≥70*%* expression heritability and could therefore facilitate uncovering mechanisms underlying the origination of complex diseases. Identifying *trans*-eQTLs is challenging because of small effect sizes, tissue specificity, and a severe multiple-testing burden. Tejaas predicts *trans*-eQTLs by performing L2-regularized “reverse” multiple regression of each SNP on all genes, aggregating evidence from many small *trans*-effects while being unaffected by the strong expression correlations. Combined with a novel unsupervised k-nearest neighbor method to remove confounders, Tejaas predicts 18851 unique *trans*-eQTLs across 49 tissues from GTEx. They are enriched in open chromatin, enhancers, and other regulatory regions. Many overlap with disease-associated SNPs, pointing to tissue-specific transcriptional regulation mechanisms.

**Supplementary Information:**

The online version contains supplementary material available at (10.1186/s13059-021-02361-8).

## Introduction

The detection, prevention, and therapeutics of complex diseases, such as atherosclerosis, Alzheimer’s disease, or schizophrenia, can improve with better understanding of the genetic pathways underlying these diseases. Over the last decade, genome-wide association studies (GWASs) have identified tens of thousands of bona fide genetic loci associated with complex traits and diseases. However, it remains unclear how most of the disease-associated variants exert their effects and influence disease risk. Over 90% of the GWAS variants are single-nucleotide polymorphisms (SNPs) in non-coding regions [1], potentially regulating gene expression that influence disease risk. Indeed, eQTL mapping has identified many genetic variants that affect gene expression. These have been mostly limited to *cis*-eQTLs, which modulate the expression of proximal genes (usually within ±1 Mbp), while little is known about *trans*-eQTLs, which modulate distal genes or those residing on different chromosomes.

The discovery of *trans*-eQTLs is critical to advance our understanding of causative disease pathways because they account for a large proportion of the heritability of gene expression. Several recent studies converge on an estimate of 60%-90% genetic variance in gene expression contributed by *trans*-eQTLs and only 10–40% by *cis*-eQTLs (see Table 1 in [2] for an overview). The recently proposed omnigenic model of complex traits highlights the importance of *trans*-regulated networks in understanding causative disease pathways [2, 3]. According to this model, most of the genetic variance is driven by weak trans effects of peripheral genes on a set of core genes, which in turn affect the risk to develop the disease.

However, in contrast to *cis*-eQTLs, *trans*-eQTLs are notoriously difficult to discover. Standard eQTL methods perform simple regression of each gene on all SNPs. Such methods have been routinely and successfully used for predicting *cis*-eQTLs, where the number of association tests is limited to SNPs in the vicinity of each gene. However, for *trans*-eQTLs, testing all genes against all SNPs imposes a hefty multiple testing burden. The major impediment, however, comes from the small effect sizes of *trans*-eQTLs on individual genes. Moreover, combining signals across multiple tissues is hindered by the tissue-specificity of *trans*-eQTLs. Such methods would therefore require enormous sample sizes—more than one million by some estimates [4]—to reliably identify *trans*-eQTLs, and it will take years to develop such resources.

Several alternative strategies have been proposed to discover *trans*-eQTL associations. The standard practice is to search for *trans*-eQTLs among restricted sets to reduce the multiple testing burden, for instance among trait-associated SNPs [5] or among SNPs with significant *cis*-associations [6]. A few methods have been developed to find *trans*-eQTLs using distinctive biological signatures. For example, GNetLMM [7] implicitly assumes that a *trans*-eQTL targets a *trans*-eGene via an intermediate *cis*-eGene. Their method tests for association between the SNP and the candidate gene using a linear mixed model, while conditioning on another set of genes that affect the candidate gene but are uncorrelated to the *cis*-eGene. Another method [8] used tensor decomposition to succinctly encode the behavior of coregulated gene networks with latent components that represent the major modes of variation in gene expression across patients and tissues, testing for association between SNPs and the latent components. A class of methods using mediation analysis try to identify the genetic control points or *cis*-mediators of gene co-expression networks [9–11]. These methods regress the candidate *trans*-eGene on the *cis*-eGene (not on the SNP) by adaptively selecting for potential confounding variables using the SNP as an “instrumental variable.” More recently, a method for imputing gene expression was used to learn and predict each gene’s expression from its *cis*-eQTLs, and then the observed gene expressions were tested for association with the predicted gene expressions to find *trans*-eGenes [12].

*Trans*-eQTLs are believed to affect the expression of a proximal diffusible factor such as a transcription, RNA-binding or signaling factor, chromatin modifier, or possibly a non-coding RNA, which in turn directly or indirectly affects the expression of the trans genes [13]. It is therefore expected that *trans*-eQTLs affect tens or hundreds of target genes in trans. Many examples in humans (see, e.g., [14, 15]) and strong evidence in yeast [16] support this hypothesis. If this information could be used effectively to discover *trans*-eQTLs, it might easily compensate their weaker effect sizes and multiple testing burden.

We expect the target genes to have more significant *p* values for association with their *trans*-eQTL than expected by chance. Brynedal et al. [17] presented a method (CPMA) that tests whether the distribution of regression *p* values for association of the candidate SNP with each gene expression level has an excess of low *p* values arising from the association of the target genes with the SNP. However, the *p* values inherit the strong correlation from their gene expressions. Therefore, if one gene has a *p* value near zero by chance, many strongly correlated genes will also have very low *p* values. This makes it difficult to decide if an enrichment of *p* values near zero is due to trans genes or due to chance, diminishing the power of the method significantly.

**Fig. 1:**
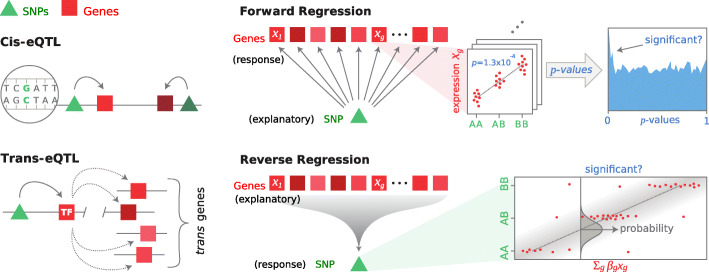
Forward and reverse regression for *trans*-eQTL discovery. *Trans*-eQTLs affect multiple genes simultaneously by exerting a *cis*-effect on a diffusible *trans*-acting factor such as a transcription factor (TF) (left). In forward regression (FR), we perform univariate regression of the expression level of each gene individually on the candidate SNP’s genotype (= centered minor allele frequency) and estimate whether the distribution of resulting association *p* values is enriched near zero. In reverse regression (Tejaas), we perform L_2_-regularized multiple regression of the candidate SNP’s genotype jointly on all gene expression levels. Crucially, reverse regression is not negatively affected by correlations between gene expression levels

Here, we circumvent the problem by reversing the direction of regression (Fig. 1). Instead of regressing each expression level on the SNP’s minor allele count, we perform multiple regression of the SNP on *all* genes jointly. In this way, no matter how strong the correlations, they do not negatively impact the test for association between gene expressions and SNP. This approach brings two decisive advantages: First, the information from each and every target gene is accumulated while automatically taking their redundancy through correlations into account. Therefore, the more target genes a SNP has, the more sensitive reverse regression will be, even when individual effect sizes are much below the significance level for individual gene-SNP association tests. Second, the multiple testing burden is reduced in comparison to single SNP-gene tests because association is tested for all genes at once.

We developed an open-source software Tejaas in Python/C++ that implements a complete pipeline for identifying *trans*-eQTL SNPs and their target genes from genotype and RNA-Seq expression data. It uses a novel, nonlinear, nonparametric and unsupervised K-nearest neighbor clustering to correct for unknown confounder variables. Predicted *trans*-eQTLs are ranked by *p* values and possible target genes are reported with their single SNP-gene association *p* values. Note that in the remainder of the manuscript, “predicted *trans*-eQTLs” refers to both true and false associations identified as *trans*-eQTL SNPs.

We applied Tejaas to the Genotype Tissue Expression (GTEx) dataset and predicted 18851 unique *trans*-eQTLs in 49 tissues with a *p* value threshold for genome-wide significance of *p*<5×10^−8^, which corresponds to false discovery rates below 5%. These putative *trans*-eQTLs are significantly enriched in various functional genomic signatures such as chromatin accessibility, functional histone marks, and reporter assay annotations and are also enriched among GWAS SNPs associated to various complex traits.

## Results

### Methods overview

Tejaas (Trans-EQTLs by Joint Association AnalysiS) computes the Reverse Regression RR-score *q*_rev_ to discover and rank *trans*-eQTLs, making use of the expectation that each *trans*-eQTL has multiple target genes. To our knowledge, only one other method makes use of it, the “forward” regression method CPMA by Brynedal et al. [17]. In order to compare Tejaas with CPMA, we implemented our own version of Forward Regression (FR) within Tejaas, as there is no publicly available software for CPMA. We used MatrixEQTL [18] as representative of all methods using single SNP-gene regression.

The FR-score *q*_fwd_ and the RR-score *q*_rev_ are summarized in Fig. 1. For details, see Online Methods and Additional file 1: Section S1 and S2. The FR score evaluates the distribution of the *p* values for the pairwise linear association of a candidate SNP with each of the *G* gene expression levels. SNPs without *trans*-effect should have uniformly distributed *p*-values, while we expect *trans*-eQTLs to have a distribution that is enriched near zero, contributed by their target genes.

Reverse Regression (RR) performs a multiple linear regression using expression levels of all genes to explain the genotype of a candidate SNP. Let **x** denote the vector of centered minor allele counts of a SNP for *N* samples and **Y** be the *G*×*N* matrix of preprocessed expression levels for *G* genes. Analogous to a common practice of modeling binary GWAS traits using linear instead of logistic regression for ease of computation [19], we model **x** using linear regression,

1 $$ p\left({\mathbf{x} \mid \mathbf{Y}} \right) \propto \mathcal{N}\left({\mathbf{x} \mid \boldsymbol{\beta^{\mathsf{T}}\mathbf{Y}, \mathbb{I}\sigma^{2}}}\right)  $$

where ***β*** is the vector of regression coefficients. Generally, the number of explanatory variables (genes) is much larger than the number of samples (*G*≫*N*) in currently available eQTL data sets. To avoid overfitting, we introduce a normal prior on ***β***, with mean 0 and variance *γ*^2^,

2 $$  p(\boldsymbol{\beta}) = \mathcal{N}\left({\boldsymbol{\beta} \mid 0, \gamma^{2}}\right).  $$

This *L*_2_ regularization pushes the effect size of non-target genes towards zero. We calculated the significance of the *trans*-eQTL model (***β***≠***0***) compared to the null model (***β*** = ***0***) using Bayes theorem to obtain

3 $$  \ln\left(\frac{P\left({\boldsymbol{\beta} \neq \boldsymbol{0} \mid \mathbf{x}, \mathbf{Y}}\right)}{P\left({\boldsymbol{\beta}} = \boldsymbol{0} \mid \mathbf{x}, \mathbf{Y}\right)}\right) = \frac{1}{2} \mathbf{x}^{\mathsf{T}}\mathbf{W}\mathbf{x} + \text{const}  $$

with

4 $$ \mathbf{W} := \frac{1}{\sigma^{2}}{\mathbf{Y}^{\mathsf{T}}}\left(\mathbf{Y}\mathbf{Y}^{\mathsf{T}}+ \frac{\sigma^{2}}{\gamma^{2}}\mathbb{I}_{G}\right)^{-1}\mathbf{Y}.   $$

We therefore defined the RR-score as *q*_rev_:=**x**^T^**Wx**.

The null distribution of *q*_rev_ is different for every SNP and can be obtained by randomly permuting the sample labels of the genotype multiple times. Although it is computationally infeasible to obtain the null distribution empirically for each SNP independently, we were able to analytically calculate the expectation and variance of *q*_rev_ under this permuted null model (Additional file 1: Appendix 1). Assuming that the null distribution is Gaussian, which holds well in practice (Additional file 1: Figure S1 and Section S2.6), we calculate a *p* value to get the significance of any observed *q*_rev_.

The assumption of normality of the RR-score null distribution breaks down when standard confounder correction methods are used (Additional file 1: Figure S2, Section S2.6 and Section S3.1). Therefore, we developed a novel, non-parametric, non-linear confounder correction using k-nearest neighbors, which we call KNN correction (Additional file 1: section 3.2). The KNN correction does not require the confounders to be known but efficiently corrects for both hidden and known confounders (Additional file 1: Section S5.4, Figures S4, S5 and S14).

Tejaas is a fast and efficiently MPI-parallelized software (Additional file 1: Figure S3) written in Python and C++. It is open-source and released under GNU General Public License v3 (Availability of Data and Materials).

### Simulation studies

We applied Tejaas reverse regression, FR, and MatrixEQTL on semi-synthetic datasets to compare their performance in well-defined settings. The simulations also allowed us to find optimum values for the number of nearest neighbors *K* and the effect size variance *γ*^2^.

For simulations, we followed the strategy of Hore et al. [8] (Online Methods and Additional file 1: Section S4). Briefly, for each simulation with 12639 SNPs and 12639 genes, we randomly selected 800 SNPs as *cis*-eQTLs, out of which 30 were also *trans*-eQTLs. The cis target genes of the *trans*-eQTLs were considered as transcription factors (TFs) and regulated multiple target genes downstream. Some strategies were different from the work of Hore et al. to make the simulations more realistic. First, we sampled the genotype directly from real data. Second, we used the covariance matrix of real gene expression as the background noise for the synthetic gene expression. Third, we included the first three genotype principal components as confounders to mimic population substructure. The simulation parameters were chosen to reflect a conservative estimate of our expectations in reality (Additional file 1: Figure S6 and Section S4.1.3).

Ranking performance is often summarized using the area under the ROC curve (AUC), the curve of true positive rate (fraction of true positives with respect to all positives) versus false positive rate (fraction of false positives with respect to all negatives) for all thresholds. However, for prediction tasks where the number of negatives is much larger than the number of positives, as in trans eQTL discovery, most part of the ROC curve corresponds to such a high FDR (false discovery rate/type I error rate) that it is irrelevant. For example, if there are 10 times more negatives than positives, at FPR = 0.1, the number of false positives is equal to the total number of positives and hence the FDR is at least 0.5. To alleviate this deficiency, we use the partial area under the ROC curve (pAUC) up to FPR =0.1. An ideal predictor will obtain pAUC =0.1 (Additional file 1: Figure S7 and Section S4.2).

**Fig. 2:**
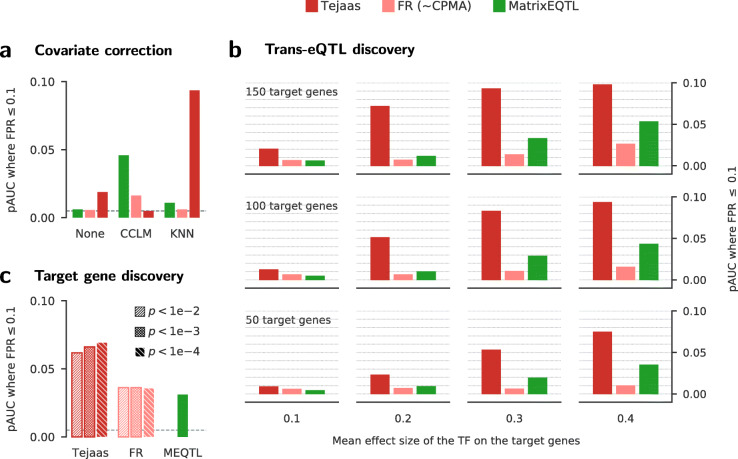
Sensitivity for *trans*-eQTL discovery on simulated data. We compared the performance of Tejaas reverse regression, forward regression (FR) (similar to CPMA) and MatrixEQTL, by computing the partial area under the ROC curve (pAUC) up to a false positive rate (FPR) of 0.1. A perfect method has pAUC = 0.1 and a random one 0.005. pAUCs are averaged over 20 simulations. **a** pAUC for different confounder correction methods: no correction (none), correction using linear regression of known confounders (CCLM) on inverse normal transformed gene expression, and our k-nearest neighbors correction with *K* = 30 (KNN). The gray dotted line corresponds to the random expectation (pAUC = 0.005). **b** pAUC for different numbers of target genes for the cis transcription factor (TF) mediating the *trans*-eQTL (from top to bottom) and different mean effect sizes of the TF on the target genes (from left to right). **c** pAUC for predicting correct SNP-gene pairs (target gene discovery) by the different methods. SNP-gene pairs were ranked by their association *p*-values. For Tejaas and FR, *trans*-eQTLs were preselected using a cutoff on their *trans*-eQTL *p*-value (legend), while MatrixEQTL does not provide any such preselection. Gray dotted line as in **a**. One simulation setting with mean TF effect size of 0.4 and 100 target genes (see Figure S11 for other simulation settings)

Figure 2a shows how the pAUC is affected by three confounder correction methods: (1) without any confounder correction (none), (2) the de facto standard method using residuals after linear regression with known confounders (CCLM), and (3) the K-nearest-neighbor correction (KNN). For Tejaas, we set *γ*=0.2 (Additional file 1: Figure S8) and *K*=30 (Additional file 1: Figures S9 and S10) empirically. To avoid false discovery of *cis*-eQTLs as *trans*-eQTLs, we masked all cis genes within ±1 Mb of each candidate SNP for Tejaas and Forward Regression (Additional file 1: Section S2.10).

The best combination of method and confounder correction is Tejaas with KNN correction (Fig. 2a). CCLM is effective for MatrixEQTL, but it does not work in combination with Tejaas because it renders the null *q*_rev_ distribution non-Gaussian and thereby leads to wrong *p* values (Additional file 1: Figure S2, Section S2.6 and Section S3.1). For FR and MatrixEQTL, CCLM works much better than KNN because we provided it with the known confounders, whereas KNN did not and can not use these. Unlike in simulations, we do not have exact knowledge of most of the confounders in real data. Hence, it is encouraging that the KNN correction works well even without knowledge of the confounders.

In Fig. 2b, we analyzed the methods’ performance depending on (1) the number of target genes of the TF linked to the *trans*-eQTL and (2) the effect size of the TF on the target genes. For the sensitivity (true positive rate) of the ranking of *trans*-eQTLs by each method in each simulation, see Additional file 1: Figure S12. For MatrixEQTL and FR, we chose the CCLM correction and for Tejaas, the KNN correction. Surprisingly, FR has slightly lower pAUC than MatrixEQTL throughout. The pAUC of Tejaas is at least twofold higher than the next best method under all conditions, although it does not use the known confounders. At mean effect size 0.2, the pAUC is up to 5 times higher than that of MatrixEQTL. The higher pAUC of Tejaas than other methods is persistent when varying the number of confounders and the effect size of confounders (Additional file 1: Figure S11).

We also compared the performance of Tejaas, FR, and MatrixEQTL to predict the target genes of *trans*-eQTLs (Fig. 2c and Additional file 1: Figure S13). For Tejaas and FR, *trans*-eQTLs were first preselected using a *p* value cutoff, and then the target genes were ranked by their SNP-gene association *p* values. Each true positive is a correctly predicted pair of *trans*-eQTL SNP and target gene; each false positive is a wrongly predicted pair. The marked improvement by Tejaas to identify target genes demonstrates that, by pre-selecting *trans*-eQTL SNP candidates, many false positive SNP-gene pairs are discarded.

### Genotype Tissue Expression *trans*-eQTL analysis

We applied Tejaas to data from the Genotype Tissue Expression (GTEx) project [20–22]. The GTEx project aims to provide insights into mechanisms of gene regulation by collecting RNA-Seq gene expression measurements from 54 tissues in hundreds of human donors, of which we used 49 tissues that have ≥70 samples with both genotype and expression measurements.

We downloaded GTEx v8 data (Availability of Data and Materials), converted the gene expression read counts obtained from phASER to standardized TPMs (Transcripts per Millions), and used the KNN correction with 30 nearest neighbors to remove confounders (Additional file 1: Section S5). Using a small hold-out test set for adipose subcutaneous tissue, we obtained *γ*=0.1. We noticed that in four tissues, this choice led to nonGaussian distributions of *q*_rev_ on null SNPs. A systematic analysis of the non-Gaussianity led to a choice of *γ*=0.006 for these remaining four tissues (Additional file 1: Figure S15 and Section S5.5). For each candidate SNP, we removed all cis genes within ±1 Mbp to avoid detecting the relatively stronger *cis*-eQTL signals and thereby inflating *q*_rev_ (Additional file 1: Figure S17). All SNPs with a genome-wide significant RR-score *p* value (*p*≤5×10^−8^) were reported as *trans*-eQTLs. To reduce redundancy, we pruned the list by retaining only the *trans*-eQTLs with lowest *p* values in each independent LD region defined by SNPs with *r*^2^>0.5.

**Fig. 3:**
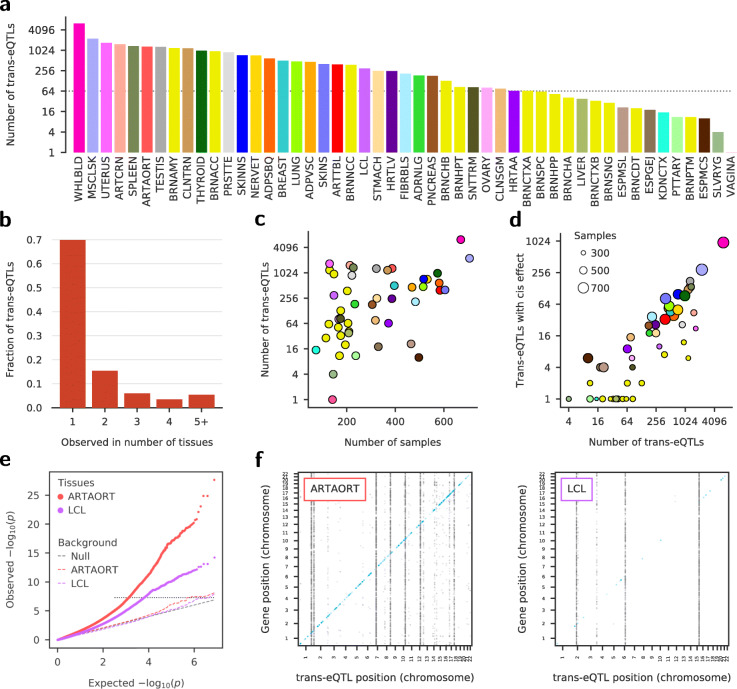
Tejaas identifies many thousands of putative *trans*-eQTLs in GTEx data. In each of the 49 GTEx tissues, we applied the KNN confounder correction and calculated genome-wide reverse regression *p* values with Tejaas. Cis genes within ± 1Mb of the candidate SNP were excluded from the regression. From the genome-wide significant SNPs (*p*<5 × 10^−8^), we selected the strongest in each LD region as lead *trans*-eQTLs, removing other SNPs in strong LD (*r*^2^≥0.5) with the lead SNP. **a** Number of lead *trans*-eQTLs discovered per tissue, on a logarithmic scale. For GTEx tissue abbreviations, see Additional file 1: Appendix 2. The dotted line indicates the cutoff used for choosing tissues for enrichment analysis. **b** Proportion of *trans*-eQTLs discovered in a given number of tissues (excluding brain tissues). Seventy percent of the lead *trans*-eQTLs are not in strong LD with any lead *trans*-eQTL from another tissue. **c** Number of lead *trans*-eQTLs discovered in a tissue (log scale) versus the number of samples for that tissue (tissue colors as in **a**). **d** About a fifth of the *trans*-eQTLs have detectable *cis*-effects. Number of lead *trans*-eQTLs versus the number of discovered lead *trans*-eQTLs that also happen to be *cis*-eQTLs in GTEx consortium analysis [6]. Tissue colors as in **a**, radii scale with sample sizes (legend). (see Fig. 4a for corresponding enrichments.) **e** Representative examples of quantile-quantile plots for artery aorta (ARTAORT) and EBV-transformed lymphocytes (LCL) with their negative controls (dashed), obtained by randomly permuting the sample IDs of genotypes. **f** Representative examples *trans*-eQTL maps for ARTAORT and LCL, with genomic positions of *trans*-eQTLs (*x*-axis) against the genomic positions of their target genes (*y*-axis). The diagonal band (blue) corresponds to *cis*-eQTLs

The LD-pruned lists contain 16929 unique lead *trans*-eQTLs in non-brain GTEx tissues and 1922 in brain tissues (Fig. 3a). For comparison, the latest analysis by the GTEx consortium on the same data yielded 142 *trans*eQTLs across 49 tissues analyzed at 5% false discovery rate (FDR), of which 41 were observed in testis [6]. To get a rough estimate of our FDRs at the cutoff *p* value of 5×10^−8^, we note that the expectation value of the number of false positive predictions for 8×10^6^ tested SNPs per tissue is about 0.4, and even less after LD-pruning. Hence, for a tissue with *T* predicted *trans*-eQTLs below the cutoff *p* value, the FDR should be roughly ≤0.4/*T*. It follows that 47 out of 49 tissues have FDRs at cutoff below 5% with many much below that.

**Fig. 4:**
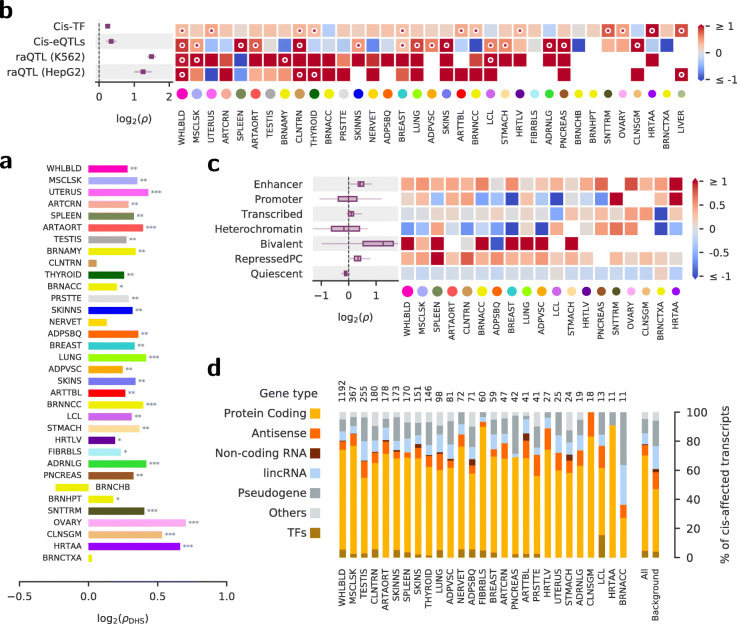
Lead *trans*-eQTLs are enriched in open chromatin and regulatory regions. **a** Log_2_ enrichments (x-axis) within accessible chromatin regions from [24]. The significance is denoted by * for *p* ≤0.05, ** for *p* ≤0.01, and *** for *p* ≤0.001. The GTEx tissues are ordered by the number of lead *trans*-eQTLs. For tissue abbreviations, see Additional file 1: Appendix 2. **b**, Log_2_ enrichments near known eQTLs and reporter assay QTLs (raQTLs) [25]. *Cis*-TF: enrichment to occur within ±100kbp from transcription factors reported in [26]; *Cis*-eQTL: enrichment among *cis*-eQTLs SNPs reported in the GTEx v8 analysis [6]; raQTL: enrichment in raQTL regions showing enhancer-like activity in K562 or HepG2 cells [25]. Heatmap colors encode log_2_ enrichment, circular marks signify *p*<0.01. The area of the colored circles on *x*-axis labels indicates the log number of lead *trans*-eQTL associations identified. Left plot: mean log_2_ enrichment across all tissues. **c** Log_2_ enrichments within tissue-specific regulatory regions. Only tissues that could be matched to the corresponding tissue annotation in the Roadmap Epigenomics Project [27] and had at least 10 *trans*-eQTLs are shown. Enhancers and bivalently marked regions show clear enrichments for most tissues. **d** Types of transcripts affected in cis by the lead *trans*-eQTLs. Only tissues with at least 10 *cis*-affected transcripts (numbers on top) are shown

The *trans*-eQTLs are tissue-specific, with 70% occurring in single tissues (Fig. 3b). The number of *trans*-eQTLs discovered increases roughly exponentially with the number of samples (Fig. 3c) for *N*>250, pointing to the importance of sample size to discover more *trans*-eQTLs. Interestingly, about a fifth of *trans*-eQTLs in each tissue are also significant *cis*-eQTLs (Fig. 3d). The effects on the target genes could plausibly be mediated by these *cis*-eGenes. The quantile-quantile plots for two representative tissues demonstrate the enrichment in significant Tejaas *p* values, while the negative controls show the expected uniform distribution of *p* values (Fig. 3e) and no significant association in the respective Manhattan plots (Additional file 1: Figure S23), confirming the correctness of the *p* values reported by Tejaas. The maps of *trans*-eQTLs and their target genes (Fig. 3f) illustrate similar patterns as observed earlier in yeast [16].

### Functional enrichment analyses of *trans*-eQTLs

Given the known difficulties to replicate and validate *trans*-eQTLs [5, 23] and the lack of RNA-Seq datasets with coverage of tissues other than whole blood, we tested the validity of our results by analyzing the enrichment of the predicted *trans*-eQTLs in functionally annotated genomic regions. One would expect only true eQTLs to be enriched in these regions. The functional enrichment measurements were compared to a set of randomly chosen SNPs from the GTEx genotypes (Additional file 1: Section S5.6). The *trans*-eQTLs were discovered excluding all genes in the vicinity of that SNP, and therefore, it is unlikely to observe functional enrichments driven by falsely discovered *cis*-eQTLs.

In Fig. 4, we show the functional enrichment of tissues which had more than 64 *trans*-eQTLs, as indicated by the dotted line in Fig. 3a. This mostly includes non-brain tissues. With low number of *trans*-eQTLs, enrichment analyses would be statistically unreliable, as for example, observed when comparing all the brain tissues (Additional file 1: Figure S22).

DNase I hypersensitive sites (DHSs) mark accessible regions of the chromatin and could indicate regulatory or biochemical activity, such as promoters, enhancers, or actively transcribed regions. Predicted *trans*-eQTLs occur more often than expected by chance within the DHS regions measured and aggregated across 125 cell and tissue types [24], with significant positive DHS enrichment (*p*≤0.05) in 30 out of 34 tissues and a *p* value ≤0.01 in 26 tissues (Fig. 4a). Using data available in the GTEx Portal, we also found enrichment across a range of annotated regulatory elements such as enhancers and transcription binding sites (Additional file 1: Figure S16). The enrichment in open chromatin and annotated regulatory regions suggest that the predicted *trans*-eQTLs possess regulatory activity more often than expected by chance.

*Trans*-eQTLs may also act via *cis*-eQTLs, where the *cis*-eGene (for example, some known TF) regulates other distant genes. Indeed, we observed a significant enrichment of *trans*-eQTLs being also *cis*-eQTLs [6] in the same tissue (Fig. 4b). The *cis*-eGenes of the novel *trans*-eQTLs have a higher proportion of protein-coding genes than the background distribution of all GTEx *cis*-eGenes (orange, Fig. 4d). Although the *cis*-affected genes are not enriched in TFs (gold, Fig. 4d), the *trans*-eQTLs are enriched proximal (≤ 100Kb) to TFs (first line in Fig. 4b).

In Fig. 4b, we show the enrichment of the *trans*-eQTLs being also reporter assay QTLs (raQTLs) for two cell types, K562 and HepG2 [25]. Reporter assay QTLs (raQTLs) are SNPs that affect the activity of promoter or enhancer elements. K562 is an erythroleukemia cell line with strong similarities to whole blood tissue and HepG2 cells are derived from hepatocellular carcinoma with similarities to liver tissue. The *trans*-eQTLs from whole blood and liver are strongly enriched (*p*<0.01), suggesting that at least some *trans*-eQTLs act via altering the activity of putative regulatory elements in a cell type-specific manner.

With the high sensitivity to discover *trans*-eQTL by Tejaas, it becomes possible to describe and disentangle tissue-specific enrichments. Using chromatin state predictions from a set of tissues from the Roadmap Epigenomics Project [27], we show that the *trans*-eQTLs are enriched in enhancer, bivalent, and repressed polycomb regions of their matched tissues (Fig. 4c). As expected, they are depleted in the inaccessible heterochromatin regions for most of the tissues.

We checked for possible confounding due to population substructure and cross-mappable genes (by ambiguously mapped reads). Some of the *trans*-eQTLs have quite different allele frequencies between GTEx subpopulations (Additional file 1: Figure S20). After adapting our null background to match the distribution of allele frequency differences (between subpopulations) of the predicted *trans*-eQTLs, the enrichments in DHS and GWAS are not significantly affected (Additional file 1: Figure S21). Saha et al. [28] had earlier raised the concern of false trans signals from ambiguously mapped reads. With cross-mappability filter, thousands of genes are removed from the expression data, which necessitates re-estimating the prior *γ* (Additional file 1: Table S1, Figure S18 and Section S5.9). We found similar enrichment in DHS and *cis*-eQTLs even after masking all possible cross-mappable genes for each tested SNP (Additional file 1: Figure S19).

### Association with complex diseases

**Fig. 5:**
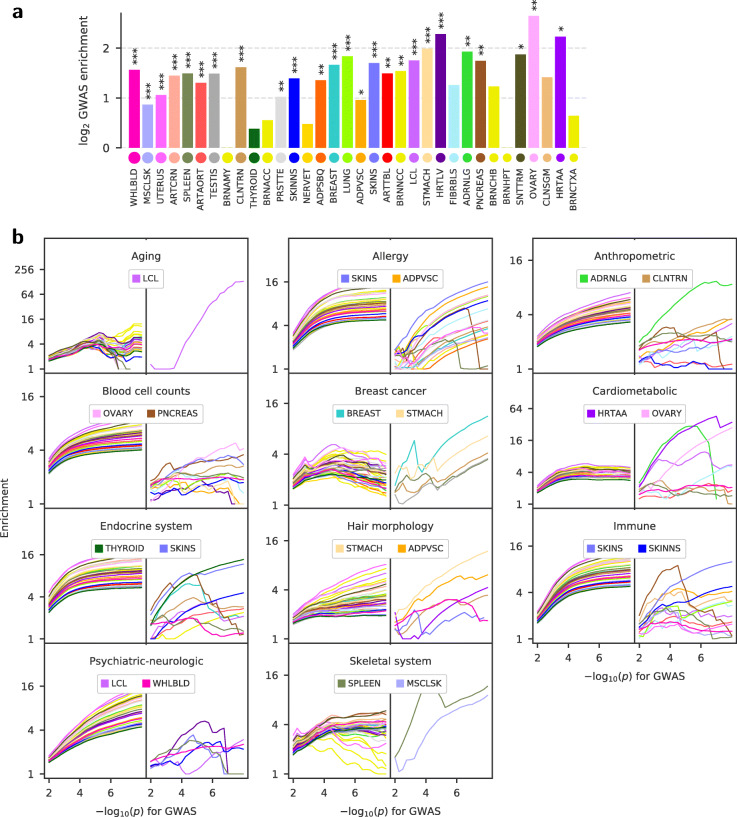
*Trans*-eQTLs are enriched among GWAS risk SNPs for complex diseases. **a***Trans*-eQTLs are enriched with SNPs from the GWAS Catalog. Significance is denoted by * for *p* ≤0.05, ** for *p* ≤0.01, and *** for *p* ≤0.001. **b** Enrichment of *cis*-eQTLs (left panel) identified by GTEx consortium and enrichment of *trans*-eQTLs (right panel) identified by Tejaas for 11 disease categories compiled from 86 GWAS of complex diseases [30] (tissue colors as in Fig. 3a). The enrichment generally increases with decreasing *p* value cutoff (*x*-axis) for the GWAS-associated SNPs. Only those tissues with significant enrichment (*p* ≤0.05) at a cutoff of *p* = 1 ×10^-6^ are shown in the plot. While *cis*-eQTLs are enriched for the majority of tissues, enrichment of *trans*-eQTLs in any disease category is tissue-specific, and the top two tissues with maximum enrichments are noted in the legends

We investigated the overlap between *trans*-eQTLs discovered by Tejaas and GWAS variants to search for *trans*-regulatory mechanisms that affect complex diseases. First, we checked for every tissue, whether more *trans*-eQTLs overlap with GWAS catalog SNPs [29] than expected by chance. Out of the 28 tissues that have more than 100 lead *trans*-eQTLs, 27 tissues showed positive enrichment in the GWAS catalog SNPs (Fig. 5a). Twenty-one tissues had an enrichment *p* value *p*≤0.05, 20 had *p*≤0.01, and 15 had *p*≤0.001. The GWAS catalog SNPs overlapping the *trans*-eQTLs are associated with a wide range of traits, many of which are not related to complex diseases.

To focus on associations with complex diseases, we used the imputed GWAS summary statistics of 87 complex diseases compiled by Barbeira et al. [30]. After filtering for significant GWAS SNPs among the GTEx genotype, there were 86 traits which were broadly classified into 11 disease categories. We found that the predicted *trans*-eQTLs from specific tissues are enriched among SNPs associated with various disease categories, as shown in Fig. 5b. The enrichment of predicted *trans*-eQTLs increases as we decrease the *p* value threshold for the GWAS-associated SNPs. Despite the much greater challenges to predict *trans*-eQTLs than to predict *cis*-eQTLs, the enrichments of the predicted *trans*-eQTLs are in a similar range to the enrichment of *cis*-eQTLs for the most enriched tissues. In contrast to the *cis*-eQTLs, the *trans*-eQTL enrichments indicate tissue specificity of *trans*-eQTLs within each of the disease categories. Several tissue–disease category associations are suggestive of a physiological link. For example, *trans*-eQTLs discovered in heart atrial appendage (HRTAA) and transformed lymphocytes (LCL) are enriched among SNPs associated with cardiometabolic disease. Other suggestive tissue–disease category associations are thyroid gland with endocrine diseases, breast tissue with breast cancer, skeletal muscle (MSCSK) with skeletal system diseases, visceral adipose (ADPVSC) tissue with allergy, and adrenal glands (ADRNLG) with anthropometric traits. All tissue–disease category associations with significant enrichment (*p*≤0.05) for *trans*-eQTLs at a nominal GWAS cutoff of *p*≤1×10^−6^ are listed in Additional file 1: Table S2. Some associations could hint at interesting, unknown roles of certain tissues in specific diseases, for instance transverse colon (CLNTRN) anthropometric traits, or whole blood (WHLBLD) with psychiatric-neurologic diseases. Further insight can be obtained from the disease-specific enrichment for each tissue in Additional file 1: Figure S23, such as stomach (STMACH) *trans*-eQTLs enriched in SNPs associated with Crohn’s disease or thyroid *trans*-eQTLs enriched in SNPs associated with hypothyroidism.

To investigate possible implications and mechanisms of the *trans*-eQTL associations identified by Tejaas, we focused on *trans*-eQTLs found in tissues that are suggestive of a physiological relation to their associated GWAS traits. For each of them, we examined their top 20 target genes.

SNP rs60977503 (chr2:217006659), predicted to be a *trans*-eQTL in breast tissue, overlaps with a GWAS hit in estrogen receptor-negative breast cancer. Among the top 20 predicted target genes of rs60977503, we found four genes associated with breast cancer. These include FAM183A, which is upregulated in breast cancer cells in response to Notch signaling [31]; MUC4, expressed in 95% of breast carcinomas [32]; HSPB6, which is downregulated in breast cancer [33, 34]; and CCL28, which promotes breast cancer proliferation, tumor growth and metastasis [35].

Similarly, SNP rs4538604, predicted as a *trans*-eQTL in stomach, resides in the inflammatory bowel disease (IBD) 5 locus that has also been associated with Crohn’s disease [36]. Some of its *cis*-genes have been linked to the disease, such as RAPGEF6, implicated in recovery after mucosal injury [37] and SLC22A5 [38]. Among the top predicted trans target genes of rs4538604 is the receptor for the chemotactic and inflammatory peptide anaphylatoxin C5a (C5AR1). It has been found to be differentially expressed in ulcerative colitis patients [39] and IBD patients that respond to Anti-TNF *α* [40]. The *trans*-targets RPS21 and ZNF773 are also associated with colorectal cancer [41, 42]. At least seven other GWAS hits associated with Crohn’s disease overlap with *trans*-eQTLs, four in small intestine and two more in spleen tissue [43], highlighting the potential relevance of our predictions.

As a third example, rs12040085 is a predicted *trans*-eQTL in adipose visceral tissue in the 1p33 locus. This region is a GWAS locus related to body mass index (BMI) and body fat percentage. Eight of the top 20 predicted trans target gene of rs12040085 are directly associated with BMI, obesity, and body height. Four of them, CDIN1 (chr15), LINGO1 (chr15), LINC01184 (chr5), and LOC105369911 (chr12), lie within reported GWAS loci related to BMI, body height, and obesity and are located on different chromosomes from rs12040085 [44–47]. The target genes TRDMT1, ZNF418, NAT1, and CDC7 have been experimentally associated through their expression levels or through knockouts, or are used as biomarkers, for waist circumference, BMI, obesity, or insulin resistance [48–52].

These examples point to the important role that *trans*-eQTLs could play in complex diseases. It will of course require larger analysis and more automated methods to integrate multiple data sources for finemapping and analyzing all predicted candidates. All our results and scripts used in this study are made public to facilitate further analyses.

## Discussion

Most applications of regression follow the assumed direction of cause and effect. The effect is used as the response variable and the potential causes are the covariates. Here, we propose to turn the direction around, using gene expression levels (the effects) as covariates and the SNP (the potential cause) as response. This reverse regression approach allows us to aggregate their explanatory signal from hundreds of gene expression levels while being unaffected by their strong correlations.

We created a fast, parallelized, open-source software and showed its power using semi-synthetic data. With its combination of reverse regression and KNN correction, Tejaas is more powerful than other existing methods to predict *trans*-eQTLs. We combined reverse regression with a method for SNP-gene association testing to identify the target genes of a discovered *trans*-eQTL because the *L*_2_ regularization does not encourage sparsity and therefore is not suited for selecting the most informative covariates.

The new KNN correction is a simple but efficient method for removing confounders. It can correct out non-linear confounding effects; therefore, it should work even if those effects are not well approximated by linear, additive models. It also does not require the confounders to be known. For future eQTL pipelines, it could prove to be very useful when applied after correcting the known confounders with linear methods.

We applied Tejaas on the GTEx dataset and predicted thousands of *trans*-eQTLs at genome-wide significance. To our knowledge, these results represent the first systematic large-scale identification of *trans*-eQTL associations in the GTEx dataset. Simple regression of SNP-gene pairs could not have discovered those *trans*-eQTLs because of their low effect sizes. Forward regression, on the other hand, is impeded by the strong correlated noise of the gene expression levels [17].

The large number of observed *trans*-eQTLs allowed us to obtain statistically significant enrichments for them in regions characterized as functional or regulatory according to various independent experimental genome-wide procedures. So far, most studies have identified too few *trans*-eQTLs for such an analysis. Large-scale meta-analysis projects had inherent selection biases which did not allow for enrichment analyses. For example, the meta-analysis of 31684 individuals on whole blood by the eQTLGen consortium [5], which predicted 3853*trans*-eQTLs, tested only GWAS-associated SNPs for *trans*-effects. Consequently, the discovered *trans*-eQTLs inherited the enrichments of the GWAS-associated SNPs.

One major source of false *trans*-eQTL predictions could be population substructure. False associations between SNPs and gene expression levels can arise if both of them are influenced by subpopulation membership, for example via life style or via epistatic effects with the genetic background. We would expect such false positive *trans*-eQTLs to show up in several tissues. The observation that 70% of the predicted *trans*-eQTLs are tissue-specific and only ∼5% are found simultaneously in 5 or more tissues (Fig. 3b) indicates that false positives do not make up a large part of our predictions. Some of the *trans*-eQTLs have quite different allele frequencies between populations, but subsequent analyses using matched null background showed significant DHS enrichment and GWAS enrichment (Additional file 1: Figure S21). This suggests weak, if any, confounding by population substructure in our approach.

The aggregation of weak signals from many covariates in Tejaas is reminiscent of the burden test [53] and the sequence kernel association test (SKAT) [54, 55], which were developed for finding rare genetic variants associated with a trait. Whereas burden and SKAT ask whether to reject the null hypothesis ***β***=***0***, Tejaas uses Bayesian model comparison of the null model ***β***=0 with the alternative model ***β***≠***0*** (Eq. (3)) while integrating out the unknown effect strengths ***β***. For *γ*→0, Tejaas’ test statistic (*q*_rev_) tends towards the unweighted SKAT statistic (Additional file 1: Section S2.7). However, Tejaas predictions were clearly better for larger *γ* values (Additional file 1: Figure S8b).

There are several limitations to our method. First, the normality assumption of the null model depends on the choice of the prior *γ* in Eq. (2). As expected, a high value of *γ* (>0.2) could lead to overfitting, whereas a low value (e.g. *γ*<0.001) can severely reduce the sensitivity to discover *trans*-eQTLs. *γ* has to be set depending on the input gene expression using the simple procedure described in Additional file 1: Section S2.8. As discussed, four out of 49 tissues in GTEx required a different setting of *γ* from the rest. Second, the input gene expression cannot be corrected for confounders using the standard approach of regressing the known confounders or hidden PEER factors [56] (Additional file 1: Section S3.1). Third, Tejaas was developed to aggregate weak effects across many target genes to detect *trans*-eQTLs, and it may not pick up strong, single SNP-gene associations like standard *trans*-mapping methods. Therefore, Tejaas and standard *trans*-mapping methods are complementary, and we expect rather low overlaps between *trans*-eQTLs predicted by these two approaches. However, the weak *trans*-effects predicted by Tejaas might be detected by standard *trans*-mapping when using a sufficiently large sample size, for example, the eQTLGen whole blood meta-analysis with 31684 individuals [5]. Only 0.96% of the eQTLGen *trans*-eQTLs overlap with the putative *trans*-eQTLs predicted by Tejaas on GTEx data (enrichment *p* value ≈3×10^−9^ compared to random overlap; Additional file 1: Appendix 3). This could in part be due to the complementary nature of the analyses and in part by the prediction of false positive associations. Finally, although we report the Benjamini-Hochberg adjusted *p* values for the target genes of the *trans*-eQTLs, they suffer from two drawbacks: (1) since we select the candidate *trans*-eQTL SNPs based on their association with gene expression levels (double dipping) [57], the *p* values of the SNP-gene pairs are not uniformly distributed under the null model any more. Therefore, the FDR adjustment can result in too optimistic values. (2) The i.i.d. assumption for the *p* values is not correct due to correlation among the gene expression levels, leading to correlated *p* values and miscalibrated FDR adjustments. Hence, the adjusted *p* values can only serve to rank target genes for any given *trans*-eQTL, but they are neither directly comparable with standard *trans*-mapping FDR-adjusted *p* values nor between different *trans*-eQTLs.

Tejaas is to our knowledge the first method whose sensitivity for *trans*-eQTL discovery does not depend on the presence of a cis effect, because cis genes are masked before reverse regression. The *trans*-eQTLs are therefore unbiased with respect to potential cis effects. We can detect a significant cis effect for about a fifth of the predicted *trans*-eQTLs in most tissues, which is more than expected by chance (*p*<0.01 for most tissues, Fig. 4b). However, if *trans*-eQTLs act via diffusible factors as is generally believed, why do not all *trans*-eQTLs have a cis effect? First, some diffusible factors might be as yet unannotated non-coding RNAs. Second, it is likely that we cannot detect the cis effects for a good fraction of *trans*-eQTLs because of low signal-to-noise ratios. Third, cis and trans effects might not occur in the same tissue, and fourth, the cis effects might have an influence on cellular or organismal decisions that amplify them enormously. For example, some SNPs might influence the bias in cell differentiation (such as B versus T cells), which impacts cell type composition. Others influence the threshold for switching on or off certain pathways such as for producing insulin. The consequences of such decisions would strongly manifest themselves in the gene expression levels as trans effects, while the cis effects would only be present in a tiny number of cells that might even reside in a different tissue. For example, the small number of hematopoietic stem cells in the bone marrow would influence blood cell composition, and beta cells producing insulin in the pancreas would influence gene expression in the liver, muscle, and adipose tissues.

Robust identification of *trans*-eQTLs will help us to dissect the interplay between genetic variation, expression levels of genes and the risk for complex diseases. We will need to further increase the number of samples in eQTL datasets. In addition, we need statistical methods with high sensitivity and accuracy to discover *trans*-eQTLs. We are working on a Bayesian approach for target gene discovery that employs a sparsity-enforcing spike-and-slab prior for the effect sizes, which has been previously used with success in other contexts such as GWAS fine-mapping [58, 59]. In summary, Tejaas represents a major step towards this goal and predicts about two orders of magnitude more *trans*-eQTLs on the GTEx v8 dataset than the state of the art at <5*%* false discovery rate. We hope that Tejaas will help to realize the tremendous value of the RNA-seq eQTL datasets that are already available or in production.

## Methods

### Forward Regression

For each SNP, we calculated the *p* values of association with all the *G* genes independently. Under the null hypothesis that the SNP is not a *trans*-eQTL, these *p* values will be independent and identically distributed (iid) with a uniform probability density function,

5 $$ {p} \sim \text{Unif}\left(0, 1\right).   $$

We sort the *p* values in increasing order; the *k*th smallest value is called the *k*th order statistic and is denoted as *p*_(*k*)_. Then, *p*_(*k*)_ will be a Beta-distributed random variable,

6 $$ p_{(k)} \sim \text{Beta}\left(k, G+1-k\right).  $$

and the expectation of ln(*p*_(*k*)_) will be

7 $$ \mathbb{E}\left[{\ln \left(p_{(k)}\right)}\right] = \psi\left(k\right) - \psi\left(G + 1\right)  $$

where *ψ* denotes the digamma function. If the candidate SNP is a *trans*-eQTL and there is an enrichment of *p* values near zero, then the cumulative sum of $\mathbb {E}\left [{\ln \left (p_{(k)}\right)}\right ] - \ln (p_{(k)})$ over *k* will increase monotonically, pass through a maximum and then decrease to an asymptotic value of zero. Hence, we defined the FR-score as,

8 $$\begin{array}{*{20}l} {q}_{\text{fwd}} &= \max_{k}\sum_{k=1}^{G} \left(\mathbb{E}\left[\ln \left(p_{(k)}\right)\right] - \ln \left(p_{(k)}\right)\right)  \\ &= \max_{k}\sum_{k=1}^{K}\left(\psi(k) - \psi(G + 1) - \ln p_{(k)}\right) \end{array} $$

It would be sufficient to calculate the *q*_fwd_ from only the first *K* genes because the rest will not contribute to the low *p* values. We obtained an empirical null distribution for *q*_fwd_ by permuting the columns of the real genotype matrix—thereby removing any association with the gene expression but retaining the correlation between the gene expression levels. For each SNP, we calculated the *p* value for *q*_fwd_ from this empirical null.

### Reverse regression

Let **x** be the genotype vector for a candidate SNP and **Y** be the *G*×*N* matrix of gene expression levels for *G* genes and *N* samples. Both **x** and **Y** are centered and normalized. We model **x** with a univariate normal distribution whose mean depends linearly on the gene expression

9 $$ P\left(\mathbf{x} \mid \mathbf{Y}, \boldsymbol{\beta}\right) \propto \mathcal{N}\left(\mathbf{x} \mid \boldsymbol{\beta}^{\mathsf{T}} \mathbf{Y}, \mathbb{I}\sigma^{2}\right).   $$

where ***β*** is the vector of regression coefficients. and *σ*^2^ is the variance of the candidate SNP. The number of samples *N* will usually be on the order of a hundred to a few thousand, much smaller than the number of explanatory variables *G*≈20000. Therefore, simple maximization of the likelihood would lead to overtrained ***β***. Hence we define a normal prior on ***β***,

10 $$ \boldsymbol{\beta} \sim \mathcal{N}\left(\boldsymbol{\beta} \mid \boldsymbol{0}, \mathbb{I}\gamma^{2}\right).   $$

Let $\mathcal {H}_{1}$ be the *trans*-eQTL model which allows ***β***≠***0*** and $\mathcal {H}_{0}$ be the null model for which ***β***=***0***. According to Bayes’ theorem,

11 $$\begin{array}{*{20}l} &P\left(\mathcal{H}_{1} \mid \mathbf{x}, \mathbf{Y}\right) \\ &= \frac{P\left(\mathbf{x} \mid \mathbf{Y}, \mathcal{H}_{1}\right)P\left(\mathcal{H}_{1}\right)} {P\left(\mathbf{x} \mid \mathbf{Y}, \mathcal{H}_{1}\right)P\left(\mathcal{H}_{1}\right) + P\left(\mathbf{x} \mid \mathbf{Y}, \mathcal{H}_{0}\right)P\left(\mathcal{H}_{0}\right)} \\ &= \left(1 + \left(\frac{P\left(\mathbf{x} \mid \mathbf{Y}, \mathcal{H}_{1}\right)P\left(\mathcal{H}_{1}\right)} {P\left(\mathbf{x} \mid \mathbf{Y}, \mathcal{H}_{0}\right)P\left(\mathcal{H}_{0}\right)}\right)^{-1}\right)^{-1}  \end{array} $$

The probability for the model $\mathcal {H}_{1}$ is a monotonically increasing function of the likelihood ratio,

12 $$\begin{array}{*{20}l} & {}\frac{P\left(\mathbf{x} \mid \mathbf{Y}, \mathcal{H}_{1}\right)}{P\left(\mathbf{x} \mid \mathbf{Y}, \mathcal{H}_{0}\right)} = \frac{\int P\left(\mathbf{x}, \boldsymbol{\beta} \mid \mathbf{Y}\right)d\boldsymbol{\beta}}{P\left(\mathbf{x} \mid \mathbf{Y}, \boldsymbol{\beta} = \boldsymbol{0}\right)} \\ &{} = \int \frac{P\left(\mathbf{x} \mid \mathbf{Y}, \boldsymbol{\beta}\right) P(\boldsymbol{\beta})}{P\left(\mathbf{x} \mid \mathbf{Y}, \boldsymbol{\beta} = \boldsymbol{0}\right)} d\boldsymbol{\beta}\\ &{}= \int \frac{1}{\left({2\pi\gamma^{2}}\right)^{G/2}} \text{exp}{\left(\frac{\boldsymbol{\beta}^{\mathsf{T}}\mathbf{Y}\mathbf{x}}{\sigma^{2}} - \frac{\boldsymbol{\beta}^{\mathsf{T}}}{2\sigma^{2}}\left({\mathbf{Y}\mathbf{Y}^{\mathsf{T}} + \frac{\sigma^{2}}{\gamma^{2}}}\right)\boldsymbol{\beta}\right)}d\boldsymbol{\beta} \\ &{} = \frac{1}{\left({2\pi\gamma^{2}}\right)^{G/2}|\boldsymbol{\Lambda}|^{1/2}} \text{exp}\left(\frac{1}{2\sigma^{2}}\mathbf{x}^{\mathsf{T}}\mathbf{Y}^{\mathsf{T}}\boldsymbol{\Lambda^{-1}\mathbf{Y}\mathbf{x}}\right),  \end{array} $$

where we have defined $\boldsymbol {\Lambda } := \mathbf {Y}\mathbf {Y}^{\mathsf {T}} + \left (\sigma ^{2} / \gamma ^{2}\right)\mathbb {I}_{G}$. The integration was done using the technique of quadratic complementation. Motivated by Eq. 12, we defined our test statistic RR-score, denoted *q*_rev_, as

13 $$\begin{array}{*{20}l} {q}\kern0.2ex_{\text{rev}} &= \frac{1}{\sigma^{2}}\mathbf{x}^{\mathsf{T}}\mathbf{Y}^{\mathsf{T}}\boldsymbol{\Lambda^{-1}}\mathbf{Y}\mathbf{x} = \mathbf{x}^{\mathsf{T}}\mathbf{W}\mathbf{x}  \end{array} $$

where

14 $$\begin{array}{*{20}l} \mathbf{W} &:= \frac{1}{\sigma^{2}}\mathbf{Y}^{\mathsf{T}}\left(\mathbf{Y}\mathbf{Y}^{\mathsf{T}} + \frac{\sigma^{2}}{\gamma^{2}}\mathbb{I}_{G}\right)^{-1}\mathbf{Y}.  \end{array} $$

### Null model

Given *q*_rev_ for the candidate SNP, we would like to know how significant this score is. We obtain the null model ${q}\kern 0.2ex_{\text {rev}}^{\text {null}}$ by permuting the elements of **x**. The distribution of ${q}\kern 0.2ex_{\text {rev}}^{\text {null}}$ will be different for every candidate SNP depending on their minor allele frequency (MAF) and the variance of the genotype (*σ*^2^). We derived analytical expressions for the expectation value $\mu _{q} := \left \langle {{q}\kern 0.2ex_{\text {rev}}^{\text {null}}}\right \rangle $ and variance $\sigma ^{2}_{q} := \text {Var}\left [ {q}\kern 0.2ex_{\text {rev}}^{\text {null}}\right ]$ under the permutation null model for any symmetric matrix **W** and any centered vector **x** (see Additional file 1: Appendix 1). Our analytical calculations of *μ*_*q*_ and *σ*_*q*_ match those obtained from the empirical permutation of **x** (Additional file 1: Figure S1). We approximate ${q}\kern 0.2ex_{\text {rev}}^{\text {null}}$ by $\mathcal {N}\left (\mu _{q}, \sigma ^{2}_{q}\right)$. Finally, the *p* value of *q*_rev_ for the candidate SNP is

15 $$  p \approx \Phi \left(\frac{{q}\kern0.2ex_{\text{rev}} - \mu_{q}}{\sigma_{q}}\right),  $$

where *Φ*(*z*) denotes the cumulative normal distribution for a random variable *z*.

### KNN correction

Gene expression measurements are notorious for being dominated by strong confounding effects and the subtle effects of *trans*-eQTLs are at risk of being drowned out by these strong systematic noise. For the KNN correction, we assume that confounding effects dominate the gene expression. If the samples are close to one another in the expression space, we expect them to be affected by the same confounders. Let **y**_*n*_ and **x**_*n*_ be the vectors of expression levels and genotypes respectively for the *n*th sample. The contribution of confounding effects on **y**_*n*_ can be corrected by removing the average expression among the *K* nearest neighbors of that sample:

16 $$\begin{array}{*{20}l}  \mathbf{y}_{n} &\leftarrow \mathbf{y}_{n} - \frac{1}{K} \sum_{m \in \text{NN}^{K}_{n}} \mathbf{y}_{m} \end{array} $$

17 $$\begin{array}{*{20}l} \mathbf{x}_{n} &\leftarrow \mathbf{x}_{n} - \frac{1}{K} \sum_{m \in \text{NN}^{K}_{n}} \mathbf{x}_{m}. \end{array} $$

The nearest neighbors $\text {NN}_{n}^{K}$ is calculated from the euclidean distances between the samples in a reduced dimension gene expression space. We also remove genotype confounders (such as population substructure) which might lead to similar gene expressions. KNN was shown to be a useful approach for many learning tasks, and since its naive form has a single parameter (*K*), overfitting does not typically occur [60, 61]. The choice of *K* should be such that it captures the locally varying effects of the confounders. A very small value of *K* would not be able to render the statistical noise, while a very large value of *K* will start removing long-range *trans*-effects (Additional file 1: Figure S10). KNN correction does not require the knowledge of known covariates, it is unsupervised and non-linear. Since KNN does not reduce the rank of the gene expression matrix, it works well with Tejaas.

### Simulation method

Simulated data consisted of genotype and gene expression for 450 individuals. After pre-filtering of the GTEx genotype, we randomly sampled 12639 SNPs. We randomly selected 800 SNPs to be *cis*-eQTLs. From these *cis*-eQTLs, we selected a subset 30 SNPs to be *trans*-eQTLs. We simulated the gene expression data for 12639 genes, containing non-genetic signals (background noise and confounding factors) and genetic signals (*cis* and *trans* effects) following the strategy of Hore et al. [8]. Each gene contained only one SNP, equivalent to assuming that there is at most one *cis*-eQTL per gene. Hore et al. used heteroscedastic background noise, but we created a correlated Gaussian noise with a covariance matrix obtained from the gene expressions in the artery aorta tissue of GTEx. We used the first three principal components of the genotype along with 7 other hypothetical covariates to generate the confounding effects. Each confounding factor was assumed to be affecting a set of randomly chosen 6320 genes with effect sizes sampled from $\mathcal {N}(0, 1)$. The strength of *cis*-effects were sampled from Gamma (4, 0.1) and the direction was chosen randomly. For the *trans*-eQTLs, the strength of *cis*-effect was constant (0.6). Additive combination of the noise, the effect of confounding factors and the effect of *cis*-eQTLs gives a temporary gene expression matrix, on top of which the effects of *trans*-eQTLs were added. The cis target gene of the *trans*-eQTLs is considered a transcription factor (TF), which regulated multiple target genes downstream. This ensured that the *trans*-eQTLs were indirectly associated with the target genes with practically low effect sizes. The effect sizes of the TF on the target genes were sampled from Gamma (*ψ*^trans^,0.02). We performed simulations with 50, 100, and 150 target genes and sampled the effect sizes of the TFs on the target genes according to a Gamma distribution with mean effect size between 0.1 and 0.4. More details about the simulations can be found in Additional file 1: Section S4.

### GTEx data and quality control

We analyzed 49 tissues with ≥70 samples with available genotype and expression measurements from the GTEx v8 project. We downloaded the genotype files and phased RNA-seq read count expression matrix. The obtained genotype was quality filtered by the GTEx consortium [6]. Genotype was split in chromosomes, variants with missing values were filtered out, and sex chromosomes were removed. 8048655 variants with minor allele frequency (MAF) ≥0.01 were retained for0 further analysis. We calculated TPMs (Transcripts Per Million) from the phASER expression matrix. We retained genes with expression values >0.1 and more than 6 mapped reads in at least 20% of the samples.

For finding target genes of the *trans*-eQTLs, we needed the explicit covariate-corrected gene expression. We downloaded the covariate files from the GTEx portal [62] and used the first 5 principal components of the genotype, donor sex, WGS sequencing platform (HiSeq 2000 or HiSeq X), and WGS library construction protocol (PCR-based or PCR-free). Additionally, from phenotype files available in dbGaP, we included donor age and post mortem interval in minutes (‘TRISCHD’) as covariates. We inverse normal transformed the TPMs and used CCLM to remove the effect of covariates.

### LD pruning

We calculated LD between variants with PLINK using an *r*^2^>0.5 within an 200 kbp sliding window. We pruned the list of *trans*-eQTLs by retaining only those lowest *p* values in each independent LD regions.

### Functional enrichment

For every functional annotation, we sampled 5000 random SNPs from the GTEx genotype. The fraction of random annotated SNPs averaged over 50 replicates gives the background frequency. The fraction of annotated *trans*-eQTLs divided by the background frequency gives the annotation enrichment. We used a binomial test to calculate the *p* values for the enrichment *ρ*. If *T* is the number of *trans*-eQTLs in the tissue, then the probability of finding *k* annotated *trans*-eQTLs is,

18 $$ P(x = k) = Binomial\left(T, k, \left\langle{f_{\text{bg}}}\right\rangle\right).  $$

where 〈*f*_bg_〉 is the background frequency and *P*(*x*>*k*) gives us the *p* value for the *trans*-eQTLs in that tissue to be enriched in the corresponding feature. See also Additional file 1: Section S5.6.

### GWAS data

We used two libraries of GWAS-associated SNPs: (i) GWAS catalog [63] and (ii) set of 87 complex trait GWAS compiled by Barbeira et al. [30] (see Additional file 1: Section S6.1). These studies were imputed and harmonized to GTEx v8 variants with MAF ≥0.01 in European samples.

### GWAS enrichment

For the GWAS catalog, we calculated the enrichment of lead *trans*-eQTLs by using the same procedure as described above for the functional enrichment. We randomly sampled 5000 SNPs from the GTEx genotype. The fraction of random SNPs that overlap with the GWAS-associated SNPs averaged over 300 replicates gives the background frequency. The fraction of lead *trans*-eQTLs that overlap with the GWAS-associated SNPs divided by the background frequency gives the GWAS enrichment.

For the set of 87 complex trait GWAS, we also compared the GWAS enrichment between *cis*-eQTLs and *trans*-eQTLs. Here, we calculated GWAS enrichment as the fraction of eQTLs (cis or trans) that overlap with GWAS-associated SNPs compared to the fraction of all tested SNPs that overlap with GWAS-associated SNPs (Additional file 1: Fig. S24). Enrichment is calculated for different *p* value cutoffs of the GWAS-associated SNPs (*x*-axis on Fig. 5b). *Cis*-eQTLs were obtained from the GTEx portal. For more details, see Additional file 1: Section S6.2.

## Supplementary Information

**Additional file 1** Supplementary text, supplementary figures S1-S25, supplementary tables S1-S2, Appendix 1-3

**Additional file 2** Review history

## Data Availability

**Tejaas and analyses pipelines.** Tejaas is released under the GNU GPL v3.0 and available at https://github.com/soedinglab/tejaas, and uploaded to Zenodo [64]. The code used for simulations is available at https://github.com/banskt/trans-eqtl-simulation. The code used for GTEx analyses is available at https://github.com/banskt/trans-eqtl-pipeline. **New results.** We have publicly released the *trans*-eQTLs discovered by applying Tejaas on GTEx data; the summary association statistics for 49 tissues are available at https://wwwuser.gwdg.de/~compbiol/tejaas/2021_04/ and uploaded to Zenodo [65]. **Source data.** This study analyzed data from the GTEx project, which are publicly available by application from dbGap (Study Accession phs000424.v8.p2). The results for the GTEx Analysis v8 were downloaded from the GTEx portal (https://gtexportal.org). The GWAS catalog was downloaded from https://www.ebi.ac.uk/gwas/home, and the GWAS summary statistics from 87 traits harmonized and imputed to GTEx v8 variants are available at 10.5281/zenodo.3657902. Reporter Assay QTLs were obtained from https://sure.nki.nl/. DHS annotations were obtained from [24] https://resources.altius.org/publications/Nature_Thurman_et_al/. Tissue-matched regulatory elements were downloaded from the Roadmap Epigenomics Project https://egg2.wustl.edu/roadmap/web_portal/chr_state_learning.html. GENCODE annotations v26 downloaded from https://www.gencodegenes.org/human/release_26.html Transcription Factors dataset was obtained from [26] http://humantfs.ccbr.utoronto.ca/download.php **Other software.** Other software used in our analyses include: MatrixEQTL [18], downloaded from http://www.bios.unc.edu/research/genomic_software/Matrix_eQTL; PLINK [66], downloaded from https://www.cog-genomics.org/plink/2.0/; LDstore [67], downloaded from http://www.christianbenner.com/; VCFTools [68], downloaded from http://vcftools.sourceforge.net/.
