# Supplementary material for: Tejaas: reverse regression increases power for detecting trans-eQTLs
Source: Genome Biol. 2021 May 6;22:142. doi: 10.1186/s13059-021-02361-8 (PMC8101255; doi:10.1186/s13059-021-02361-8)
Supplement: Supplementary file 1 — Additional file 1 Supplementary text, supplementary figures S1-S25, supplementary tables S1-S2, Appendix 1-3 [file 13059_2021_2361_MOESM1_ESM.pdf]

Additional file 1

Supplementary text, supplementary figures S1-S25, supplementary tables S1-S2, Appendix 1-3

Tejaas: Reverse regression increases power for detecting trans-eQTLs

Saikat Banerjee[†,1,*], **Franco L. Simonetti**[†,1], **Kira E. Detrois**[1,2], **Anubhav Kaphle**[1,2], **Raktim Mitra**[3], **Rahul Nagial**[3], **and Johannes Söding**[1,4,5,*]

[1]Quantitative and Computational Biology, Max-Planck Institute for Biophysical Chemistry, 37077 Göttingen, Germany
[2]Georg-August University, 37075 Göttingen, Germany
[3]Indian Institute of Technology, Kanpur, India
[4]Campus-Institut Data Science (CIDAS), University of Göttingen, Germany
[5]Cluster of Excellence "Multiscale Bioimaging" (MBExC), University of Göttingen, Germany
[*]Correspondence to : soeding@mpibpc.mpg.de, bnrj.saikat@gmail.com
[†]These authors contributed equally.

Contents

1 Forward regression **4**
- 1.1 Notation . . . 4
- 1.2 FR-score . . . 4
- 1.3 Null model and p-values for FR-score . . . 5
- 1.4 Comparison with CPMA . . . 6

2 Reverse regression **6**
- 2.1 Notation . . . 6
- 2.2 Model description . . . 6
- 2.3 Bayesian model comparison . . . 7
- 2.4 RR-score: Definition . . . 8
- 2.5 Numerical calculation . . . 8
- 2.6 Null model . . . 9
- 2.7 Comparison of RR-score with sequence kernel association test (SKAT) . . . 10
- 2.8 Choice of the regularizer . . . 12
- 2.9 Target genes . . . 13
- 2.10 Cis-masking . . . 13
- 2.11 Computational requirements . . . 13

3 Confounder correction **14**
- 3.1 Residuals from multiple linear regression . . . 14
- 3.2 K-nearest neighbor (KNN) correction . . . 14

4 Trans-eQTL simulation **17**
- 4.1 Data simulation . . . 17
  - 4.1.1 Genotypes . . . 18
  - 4.1.2 Gene expression . . . 18
  - 4.1.3 Choice of simulation parameters . . . 20
- 4.2 ROC pAUC analysis . . . 23
- 4.3 Methods compared . . . 24
- 4.4 Supplementary simulation results . . . 24

5 GTEx analysis **28**
- 5.1 Genotype data . . . 28
- 5.2 RNA-Seq expression data . . . 29
- 5.3 Covariate correction . . . 29
- 5.4 Efficacy of KNN correction . . . 30
- 5.5 Tejaas regularizer selection for GTEx . . . 31
- 5.6 Tejaas results on GTEx . . . 33
- 5.7 Method for calculating feature enrichments . . . 33
- 5.8 Regulatory element enrichment . . . 33
- 5.9 Effect of cis-masking . . . 33
- 5.10 Effect of cross-mappable genes . . . 34
- 5.11 Effect of GTEx populations in predicted trans-eQTLs . . . 36
- 5.12 Regulatory element enrichment in brain tissues . . . 37
- 5.13 Performance of Tejaas on null data . . . 38
- 5.14 Replication in eQTLGen . . . 38

6 GWAS analysis **38**
6.1 Data source for GWAS summary statistics 40
6.2 Calculation of GWAS enrichment 40
6.3 Supplementary results 43

Appendix 1. Expectation and variance of RR-score under the null hypothesis **44**
Expectation value of RR-score 45
Variance of RR-score 46

Appendix 2. Abbreviation of GTEx tissues **51**

Appendix 3. eQTLGen Replication **52**

References **53**

List of Figures

S1 Validation of analytical calculation of mean and variance of RR-score null distribution 10
S2 Effect of covariate correction on null model 11
S3 Computational time and memory requirements of Tejaas 15
S4 Normal approximation of permuted q_{rev} remains valid after KNN correction . . 16
S5 Distance between samples before and after KNN correction. 17
S6 Calibration of the choice of simulation parameters 21
S7 Definition of pAUC 23
S8 Selection of standard deviation of the regularizer in simulations 25
S9 Inverse normal transform reduces power of Tejaas 25
S10 Optimization of number of neighbors used for KNN correction 26
S11 Effect of confounding factors in finding trans-eQTLs 27
S12 Sensitivity of different methods in finding trans-eQTLs 28
S13 Comparison of ranking performance of different methods in finding target genes of trans-eQTLs 29
S14 Known covariates of GTEx can explain a significant proportion of KNN correction 30
S15 Non-Gaussian parameters for all GTEx tissues 32
S16 Trans-eQTLs are enriched in known regulatory elements 34
S17 Cis-masking controls falsely identifying cis-eQTLs as trans-eQTLs 35
S18 Effect of cross-mappable genes on trans-eQTL discovery 36
S19 Effect of cross-mappable genes on functional enrichment of trans-eQTLs 37
S20 Distribution of F_{ST} values in GTEx and predicted trans-eQTLs 37
S21 DHS enrichment of trans-eQTLs after adjusting background for F_{ST} 38
S22 Functional enrichments in brain tissues 39
S23 Performance of Tejaas on null data 39
S24 Calculation of GWAS enrichment 41
S25 Overlap with GWAS-associated SNPs suggests tissue-specific regulation mechanism of trans-eQTLs 44

List of Tables

S1 Summary of number of trans-eQTLs before and after cross-mappability filter . . 35
S2 GWAS enrichment of trans-eQTLs for different tissues and disease categories . . 41

1 Forward regression

A trans-eQTL is generally expected to influence the expression levels of tens to hundreds of genes and we take advantage of this signature to increase the sensitivity to detect them. Brynedal *et al.* proposed a method called cross-phenotype meta-analysis (CPMA) to analyze the p-value distribution of pairwise associations of a candidate SNP with all measured genes [1]. We follow the same idea but use a different statistic for forward regression (FR) to find the trans-eQTLs.

1.1 Notation

We use bold capital cases for matrices. $\mathbf{X}$ is the $I \times N$ matrix of genotypes for I SNPs and N samples. $\mathbf{Y}$ is the $G \times N$ matrix of gene expression levels for G genes and N samples. For the FR analysis, both $\mathbf{X}$ and $\mathbf{Y}$ are centered and normalized. We use bold small cases for vectors. The rows of $\mathbf{X}$ and $\mathbf{Y}$ are denoted as $\mathbf{x}_i$ and $\mathbf{y}_g$ respectively for every SNP $i \in \{1, \ldots, I\}$ and for every gene $g \in \{1, \ldots, G\}$. The columns of $\mathbf{X}$ and $\mathbf{Y}$ are denoted as $\mathbf{x}_n$ and $\mathbf{y}_n$ respectively for every sample $n \in \{1, \ldots, N\}$. Both $\mathbf{x}_i$ and $\mathbf{y}_g$ are vectors of size N, while $\mathbf{x}_n$ and $\mathbf{y}_n$ are of sizes I and G respectively.

1.2 FR-score

For each SNP $\mathbf{x}_i$, we calculated the p-values of association with $\mathbf{y}_g$ for all the $g \in \{1, \ldots, G\}$ genes independently. Under the null hypothesis that the SNP is not a trans-eQTL, these p-values will be independent and identically distributed (iid) with a uniform probability density function,

$$p \sim \text{Unif}\,(0, 1)\ . \tag{1}$$

If SNP i is a trans-eQTL, then more genes than expected by chance will have low p-values for association with the SNP, leading to a higher density of p-values near zero. We defined the FR-score (q_{fwd}) as a statistic that estimates the difference between the observed p-value distribution from the data and the uniform distribution expected by chance.

We sort the p-values in increasing order and the kth smallest value is called the kth order statistic, and is denoted as $p_{(k)}$. If p is uniformly distributed ($p \in (0, 1)$), then $p_{(k)}$ will be a Beta-distributed random variable,

$$p_{(k)} \sim \text{Beta}\,(k, G + 1 - k)\ . \tag{2}$$

For any random variable $z \sim \text{Beta}\,(\alpha, \beta)$,

$$\begin{aligned} \mathbb{E}\,[z] &= \frac{\alpha}{\alpha + \beta} \\ \mathbb{E}\,[\ln z] &= \psi\,(\alpha) - \psi\,(\alpha + \beta)\ , \end{aligned} \tag{3}$$

where ψ denotes the digamma function. Using the identities in Eq. 3 and noting that $\alpha = k$ and $\beta = G + 1 - k$, we obtained the expectation of $\ln p_{(k)}$ as,

$$\mathbb{E}\left[\ln p_{(k)}\right] = \psi\,(k) - \psi\,(G + 1) \tag{4}$$

If the candidate SNP is a trans-eQTL and there is an enrichment of p-values near zero, then the observed values of $\ln p_{(k)}$ will be lower than that expected by chance when k is small. Therefore, the cumulative sum of $\left(\mathbb{E}\left[\ln p_{(k)}\right] - \ln p_{(k)}\right)$ over k will increase monotonically, pass through a

maximum and then decrease to an asymptotic value of zero. Hence, we defined the FR-score as,

$$q_{\text{fwd}} = \max_k \sum_{k=1}^{G} \left(\mathbb{E}\left[\ln p_{(k)}\right] - \ln p_{(k)}\right) \tag{5}$$

Intuitively, if the candidate SNP is not a trans-eQTL, $\sum_{k=1}^{G} \left(\mathbb{E}\left[\ln p_{(k)}\right] - \ln p_{(k)}\right)$ will fluctuate around zero. Hence, q_{fwd} will remain close to 0 for these SNPs. However, a trans-eQTL will be associated with many genes and the FR-score will be high ($q_{\text{fwd}} \gg 0$) because there will be many genes with a lower p-value than expected by chance. Of course, there will be other genes which are not associated with the trans-eQTL and the p-values for association with these genes will not contribute to the q_{fwd}. Therefore, it would be sufficient to calculate the q_{fwd} from the first K genes with lowest p-values, instead of all G genes:

$$\begin{aligned} q_{\text{fwd}} &= \max_k \sum_{k=1}^{K} \left(\mathbb{E}\left[\ln p_{(k)}\right] - \ln p_{(k)}\right) \\ &= \max_k \sum_{k=1}^{K} \left(\psi(k) - \psi(G+1) - \ln p_{(k)}\right) \end{aligned} \tag{6}$$

1.3 Null model and *p*-values for FR-score

We need to define a null distribution to evaluate the significance of any observed q_{fwd}. Let $q_{\text{fwd}}^{\text{null}}$ be an iid sample from the null distribution. Since it is analytically intractable to define a probability density of $q_{\text{fwd}}^{\text{null}}$, we define an empirical null distribution. The empirical cumulative distribution function (ECDF) of $q_{\text{fwd}}^{\text{null}}$ can be used to ascertain significance of any observed q_{fwd} to obtain a p-value, denoted as $p_{q_{\text{fwd}}}$,

$$p_{q_{\text{fwd}}} = 1 - \text{ECDF}\left(q_{\text{fwd}}\right) \tag{7}$$

One possible way to obtain a null model is by calculating $q_{\text{fwd}}^{\text{null}}$ for a large number of *null* SNPs assuming they are not trans-eQTLs. Following our hypothesis in Eq. (1), the p-values for association of each of these *null* SNPs with the genes can be sampled each time from $\text{Unif}(0,1)$ distribution. However, the iid assumption of p-values breaks down in real data because the gene expressions are strongly correlated. Therefore, the p-values for association between the SNP and the genes are strongly correlated and cannot be sampled from the $\text{Unif}(0,1)$ distribution.

To account for the correlation, we randomized the sample labels of $\mathbf{X}$ by permuting the columns of the real genotype matrix – thereby removing any association with the gene expression $\mathbf{Y}$ but retaining the correlation between the gene expression levels. We then calculated $q_{\text{fwd}}^{\text{null}}$ for all SNPs using the 'permuted' genotype and the gene expression. We used this empirical null distribution to calculate $p_{q_{\text{fwd}}}$ from Eq. 7 .

The estimate of $p_{q_{\text{fwd}}}$ gets increasingly noisy with higher values of q_{fwd} (low p-values) because of lack of sampling points in that region. In that regime it is better to rely on a parametric fit of the exponentially falling part of the cumulative distribution. We sorted the $q_{\text{fwd}}^{\text{null}}$ in an increasing order. We set the limit beyond which we will use the exponential extrapolation at $n^{\text{top}} = \min\{100, 0.1I\}$, where I is the total number of SNPs being used for the calibration of the null model. This ensures that the extrapolation is applied at most to the top 10% of points, and, if more data are available, only to the top 100. Let us denote the $q_{\text{fwd}}^{\text{null}}$ at this cut-off point as q'. For any observed $q_{\text{fwd}} \geq q'$, we use a maximum-likelihood estimate based on the n^{top} data points and is given by,

$$p_{q_{\text{fwd}}} = \frac{n_{\text{top}}}{I} \exp\left(-\frac{q_{\text{fwd}} - q'}{\lambda}\right), \tag{8}$$

with

$$\lambda := \frac{1}{n_{\text{top}}} \sum_{j=1}^{n_{\text{top}}} \left(q^{\text{null}}_{\text{jpa},j} - q' \right) . \tag{9}$$

where $q^{\text{null}}_{\text{jpa},j}$ is the jth value in the ordered sequence of $q^{\text{null}}_{\text{fwd}}$. Note that for $q_{\text{fwd}} = q'$ this equation yields $p_{q'} = n_{\text{top}}/I$, the same as the empirical value.

1.4 Comparison with CPMA

Brynedal *et al.*used a similar idea for finding trans-eQTLs by testing each SNP for enrichment of association $-\ln(p)$ values across all genes with a null hypothesis that $-\ln(p)$ should be exponentially distributed, with a decay parameter $\lambda = 1$. Under the joint alternative hypothesis, a subset of association statistics are non null and $\lambda \neq 1$. They compared the evidence for these hypotheses as a likelihood ratio test for CPMA, with the statistic S_{CPMA} defined as,

$$S_{\text{CPMA}} = -2 \ln \left(\frac{P\left(\text{data} \mid \lambda = 1\right)}{P\left(\text{data} \mid \lambda = \hat{\lambda}\right)} \right) \tag{10}$$

where $\hat{\lambda}$ is the observed exponential parameter from the data. They accounted for the extensive correlation among the gene expression levels by testing the significance empirically using a simulated gene expression matrix with the same covariance as the real gene expression. To define high-confidence trans-eQTLs, they combined empirical CPMA statistic from multiple data sets using sample size weighted meta-analysis.

FR measures the significance of the enrichment in the p-value distribution near zero, and CPMA measures the significance of the enrichment in the $-\ln(p)$ distribution. Theoretically, both should give similar ranking of SNPs for being trans-eQTLs. Since there are no currently available software for CPMA, we implemented FR-score in Tejaas and compared the performance with RR-score.

2 Reverse regression

In this section, we discuss the reverse regression (RR) model and introduce the RR-score, denoted as q_{rev} for finding the trans-eQTLs. We also describe a null model and explain how to obtain significance p-values for the q_{rev} of a candidate SNP, denoted as $p_{q_{\text{rev}}}$. We also discuss the numerical implementation of RR-score in Tejaas and several other options used in Tejaas.

2.1 Notation

We use the same notations from Sec. 1.1. The genotype vector $\mathbf{x}_i$ for every i^{th} SNP is centered but not normalized to allow for null model calculation. $\mathbf{Y}$ is centered and normalized.

2.2 Model description

We model $\mathbf{x}_i$ with a univariate normal distribution whose mean depends linearly on the gene expression through a column vector of regression coefficients $\boldsymbol{\beta}_i$ ($\in \mathbb{R}^G$),

$$P\left(\mathbf{x}_i \mid \mathbf{Y}, \boldsymbol{\beta}_i\right) \propto \mathcal{N}\left(\mathbf{x}_i \mid \boldsymbol{\beta}_i^{\mathsf{T}} \mathbf{Y}, \sigma_i^2 \mathbb{I}_N\right) . \tag{11}$$

where the variance of the ith SNP is given as σ_i^2. For the rest of the manuscript, we drop the subscript i for ease of reading although we note that all calculations are done for the ith SNP. The

log likelihood of this regression task is

$$\begin{aligned}\ln \mathscr{L}(\boldsymbol{\beta}) &= \ln P(\mathbf{x} \mid \mathrm{Y}, \boldsymbol{\beta}) \\ &= -\frac{1}{2\sigma^2}\sum_{n=1}^{N}\left(x_n - \boldsymbol{\beta}^{\mathsf{T}}\mathbf{y}_n\right)^2 + \text{const}\,, \end{aligned} \tag{12}$$

where x_n is the minor allele count of the ith SNP in n^{th} sample. The number of samples N will usually be on the order of a hundred to a few thousand, much smaller than the number of explanatory variables $G \approx 20\,000$. Therefore, simple maximization of the likelihood would lead to a dramatically overtrained $\boldsymbol{\beta}$ which would perfectly predict $\mathbf{x}$ on the training data but which would achieve very bad performance on unseen data. The solution is to define a prior on $\boldsymbol{\beta}$. We assume that every g^{th} element ($g \in \{1, \ldots, G\}$) of $\boldsymbol{\beta}$ is sampled from a normal distribution with variance γ^2,

$$\beta_g \sim \mathcal{N}\left(\beta_g \mid 0, \gamma^2\right) \tag{13}$$

and maximize the log posterior probability,

$$\begin{aligned}\ln P(\boldsymbol{\beta} \mid \mathbf{x}, \mathrm{Y}) &= \ln \frac{P(\mathbf{x} \mid \mathrm{Y}, \boldsymbol{\beta})\, P(\boldsymbol{\beta})}{P(\mathbf{x} \mid \mathrm{Y})} \\ &= -\frac{1}{2\sigma^2}\sum_{n=1}^{N}\left(x_n - \boldsymbol{\beta}^{\mathsf{T}}\mathbf{y}_n\right)^2 - \frac{1}{2\gamma^2}\sum_{g=1}^{G}\beta_g^2 + \text{const.}\end{aligned} \tag{14}$$

2.3 Bayesian model comparison

Let $\mathcal{H}_1$ be the trans-eQTL model which allows $\boldsymbol{\beta} \neq \mathbf{0}$ and $\mathcal{H}_0$ be the null model for which $\boldsymbol{\beta} = \mathbf{0}$. According to Bayes' theorem,

$$\begin{aligned}P(\mathcal{H}_1 \mid \mathbf{x}, \mathrm{Y}) &= \frac{P(\mathbf{x} \mid \mathrm{Y}, \mathcal{H}_1)\, P(\mathcal{H}_1)}{P(\mathbf{x} \mid \mathrm{Y}, \mathcal{H}_1)\, P(\mathcal{H}_1) + P(\mathbf{x} \mid \mathrm{Y}, \mathcal{H}_0)\, P(\mathcal{H}_0)} \\ &= \left(1 + \left(\frac{P(\mathbf{x} \mid \mathrm{Y}, \mathcal{H}_1)\, P(\mathcal{H}_1)}{P(\mathbf{x} \mid \mathrm{Y}, \mathcal{H}_0)\, P(\mathcal{H}_0)}\right)^{-1}\right)^{-1}\end{aligned} \tag{15}$$

The probability for the model $\mathcal{H}_1$ is a monotonically increasing function of the likelihood ratio,

$$\begin{aligned}\frac{P(\mathbf{x} \mid \mathrm{Y}, \mathcal{H}_1)}{P(\mathbf{x} \mid \mathrm{Y}, \mathcal{H}_0)} &= \frac{\int P(\mathbf{x}, \boldsymbol{\beta} \mid \mathrm{Y})\, d\boldsymbol{\beta}}{P(\mathbf{x} \mid \mathrm{Y}, \boldsymbol{\beta} = \mathbf{0})} \\ &= \int \frac{P(\mathbf{x} \mid \mathrm{Y}, \boldsymbol{\beta})\, P(\boldsymbol{\beta})}{P(\mathbf{x} \mid \mathrm{Y}, \boldsymbol{\beta} = \mathbf{0})}\, d\boldsymbol{\beta} \\ &= \int \frac{1}{(2\pi\gamma^2)^{G/2}} \exp\left(\frac{1}{\sigma^2}\boldsymbol{\beta}^{\mathsf{T}}\mathrm{Y}\mathbf{x} - \frac{1}{2\sigma^2}\boldsymbol{\beta}^{\mathsf{T}}\left(\mathrm{Y}\mathrm{Y}^{\mathsf{T}} + \frac{\sigma^2}{\gamma^2}\right)\boldsymbol{\beta}\right) d\boldsymbol{\beta}\,,\end{aligned} \tag{16}$$

where the second line is obtained by using the model defined in Eq. 11 and the prior for $\boldsymbol{\beta}$ defined in Eq. 13. We then defined $\boldsymbol{\Lambda} := \mathrm{Y}\mathrm{Y}^{\mathsf{T}} + \left(\sigma^2/\gamma^2\right)\mathbb{I}_G$ and used the technique of quadratic complementation to obtain,

$$\begin{aligned}&\frac{P(\mathbf{x} \mid \mathrm{Y}, \mathcal{H}_1)}{P(\mathbf{x} \mid \mathrm{Y}, \mathcal{H}_0)} \\ &= \int \frac{1}{(2\pi\gamma^2)^{G/2}} \exp\left(-\frac{1}{2\sigma^2}\left(\boldsymbol{\beta} - \boldsymbol{\Lambda}^{-1}\mathrm{Y}\mathbf{x}\right)^{\mathsf{T}}\boldsymbol{\Lambda}\left(\boldsymbol{\beta} - \boldsymbol{\Lambda}^{-1}\mathrm{Y}\mathbf{x}\right) + \frac{1}{2\sigma^2}\mathbf{x}^{\mathsf{T}}\mathrm{Y}^{\mathsf{T}}\boldsymbol{\Lambda}^{-1}\mathrm{Y}\mathbf{x}\right) d\boldsymbol{\beta}\end{aligned}$$

$$= \frac{1}{(2\pi\gamma^2)^{G/2} |\boldsymbol{\Lambda}|^{1/2}} \exp\left(\frac{1}{2\sigma^2}\mathbf{x}^\mathsf{T}\mathbf{Y}^\mathsf{T}\boldsymbol{\Lambda}^{-1}\mathbf{Y}\mathbf{x}\right). \tag{17}$$

Using Eqs. 15 and 17, we obtained the probability of the trans-eQTL model,

$$P(\mathcal{H}_1 \mid \mathbf{x}, \mathrm{Y}) = \left(1 + (2\pi\gamma^2)^{G/2} |\boldsymbol{\Lambda}|^{1/2} \exp\left(-\frac{1}{2\sigma^2}\mathbf{x}^\mathsf{T}\mathbf{Y}^\mathsf{T}\boldsymbol{\Lambda}^{-1}\mathbf{Y}\mathbf{x}\right) \frac{P(\mathcal{H}_0)}{P(\mathcal{H}_1)}\right)^{-1} \tag{18}$$

Thus, it is possible to obtain the probability of each SNP being a trans-eQTL but the calculation is computationally expensive and requires the prior probabilities $P(\mathcal{H}_0)$ and $P(\mathcal{H}_1)$. These prior probabilities can be set to a constant.

2.4 RR-score: Definition

Motivated by the probability obtained in Eq. 18, we defined our test statistic RR-score, denoted q_{rev}, as follows:

$$\begin{aligned} q_{\text{rev}} &= \frac{1}{\sigma^2}\mathbf{x}^\mathsf{T}\mathbf{Y}^\mathsf{T}\boldsymbol{\Lambda}^{-1}\mathbf{Y}\mathbf{x} \\ &= \mathbf{x}^\mathsf{T}\mathbf{W}\mathbf{x} \end{aligned} \tag{19}$$

$$\text{where, } \mathbf{W} := \frac{1}{\sigma^2}\mathbf{Y}^\mathsf{T}\left(\mathbf{Y}\mathbf{Y}^\mathsf{T} + \frac{\sigma^2}{\gamma^2}\mathbb{I}_G\right)^{-1}\mathbf{Y}. \tag{20}$$

Suppose we have obtained a score q_{rev} for the ith SNP and we would like to know how significant this score is. In order to rank it with respect to all the other SNPs in the genome, we need to calculate a null distribution of $q_{\text{rev}}^{\text{null}}$ and from it the p-value of our observed score q_{rev}.

Note that the statistic q_{rev} is not proportional to the probability given by Eq. 18 and hence cannot be directly used for ranking the SNPs. However, in Sec. 2.6, we derive a p-value to ascertain the significance of the q_{rev} of each SNP.

2.5 Numerical calculation

The RR-score defined in Eq. (19) involves computing the inverse of a $G \times G$ matrix $\mathbf{W}$. To reduce the complexity, we first perform a singular value decomposition of $\mathbf{Y}^\mathsf{T}$,

$$\mathbf{Y}^\mathsf{T} = \mathbf{U}^\mathsf{T}\mathbf{S}\mathbf{V} \tag{21}$$

with two orthogonal matrices, $\mathbf{U} \in \mathbb{R}^{N\times N}$ and $\mathbf{V} \in \mathbb{R}^{G\times G}$, and a diagonal matrix $\mathbf{S} \in \mathbb{R}^{N\times G}$ that whose all elements are zero except for the $K = \min\{G, N\}$ singular values s_k on its diagonal. Substituting the SVD into $\mathbf{W}$ gives

$$\mathbf{W} = \frac{1}{\sigma^2}\mathbf{U}^\mathsf{T}\mathbf{S}\left(\mathbf{S}^\mathsf{T}\mathbf{S} + \frac{\sigma^2}{\gamma^2}\mathbb{I}_G\right)^{-1}\mathbf{S}^\mathsf{T}\mathbf{U} \tag{22}$$

This allows us to expand the matrix $\mathbf{W}$ using the singular values s_k and the eigenvectors $\mathbf{u}_k$,

$$\mathbf{W} = \frac{1}{\sigma^2}\sum_{k=1}^{K}\frac{s_k^2}{s_k^2 + \sigma^2/\gamma^2}\mathbf{u}_k\mathbf{u}_k^\mathsf{T} \tag{23}$$

Therefore, we can write the RR-score as

$$q_{\text{rev}} = \frac{1}{\sigma^2} \sum_{k=1}^{K} \frac{s_k^2}{s_k^2 + \sigma^2/\gamma^2} \left(\mathbf{u}_k^{\mathsf{T}}\mathbf{x}\right)^2 . \tag{24}$$

2.6 Null model

To obtain a p-value for the q_{rev} score of SNP i with column $\mathbf{x}_i$ in the genotype matrix $\mathbf{X}$, we need a null distribution of q_{rev} scores. The p-value is then the probability mass of the null distribution with values higher than the actually obtained q_{rev} score. We can obtain such null distribution by permuting the genotype entries in $\mathbf{x}_i$ while keeping the rows of the gene expression matrix $\mathbf{Y}$ unpermuted. The distribution of the resulting $q_{\text{rev}}^{\text{null}}$ scores can differ between SNPs depending on their minor allele frequency (MAF) and the variance of the genotype (σ^2).

In principle we could derive the distribution of $q_{\text{rev}}^{\text{null}}$ empirically by permuting the elements of $\mathbf{x}_i$ a large number of times. However, to obtain p-values of 5×10^{-8} or below, corresponding to genome-wide significance, we would need to draw at least 2×10^7 permuted samples and compute their q_{rev} scores. This would be too time consuming. Instead, we derive in Appendix 1 analytical expressions for the expectation value $\mu_q := \langle q_{\text{rev}}^{\text{null}} \rangle$ and variance $\sigma_q^2 := \text{Var}\left[q_{\text{rev}}^{\text{null}}\right]$ of $q_{\text{rev}}^{\text{null}} = \mathbf{x}^{\mathsf{T}}\mathbf{W}\mathbf{x}$ under the permutation null model for any symmetric matrix $\mathbf{W}$ and any centered vector $\mathbf{x}$. In Fig. S1a and b, we show that our analytical calculation of μ_q and σ_q match those obtained from the empirical permutation of $\mathbf{x}$. With this empirical permutation, we could verify a p-value up to 1×10^{-12} is accurately approximated by our normal approximation. In our subsequent analyses of GTEx, we found that 2781 association tests (out of 49×8048655 association tests) were beyond the empirically verified range.

Normal approximation. Under the null model assumption that the SNP is not a trans-eQTL, the $\mathbf{u}_k^{\mathsf{T}}\mathbf{x}$ are distributed as the projections of a unit vector with random direction onto K Cartesian coordinates given by the eigenvectors $\mathbf{u}_k$. The $\mathbf{u}_k^{\mathsf{T}}\mathbf{x}$ will be normally distributed and $\left(\mathbf{u}_k^{\mathsf{T}}\mathbf{x}\right)^2$ will have a chi-square distribution. In the limit of $K \gg 1$, the sum over $\left(s_k^2/(s_k^2 + \sigma^2/\gamma^2)\right)\left(\mathbf{u}_k^{\mathsf{T}}\mathbf{x}\right)^2$ will have a normal distribution according to the central limit theorem. Therefore, we approximate $q_{\text{rev}}^{\text{null}}$ by $\mathcal{N}\left(\mu_q, \sigma_q^2\right)$. In Fig. S1c, we show the QQ plot which numerically validates the approximation.

Finally, the p-value of q_{rev} for a candidate SNP is

$$p \approx \Phi\left(\frac{q_{\text{rev}} - \mu_q}{\sigma_q}\right) , \tag{25}$$

where $\Phi(z)$ denotes the cumulative normal distribution for a random variable z.

Constraint on covariate correction. Generally, the gene expression matrix Y should have rank (Y) $= K = \min\{G, N\}$. This means that the SVD in Eq. (21) should have K singular values s_k. To obtain q_{rev}, the sum in Eq. (24) runs over K components of $\left(\mathbf{u}_k^{\mathsf{T}}\mathbf{x}\right)^2$. If C known covariates are now corrected from Y using linear regression (see CCLM below, Sec. 3.1), then C of these singular values become zero for the residual expression $\mathbf{Y}'$. This is equivalent to subtracting C components from the sum in Eq. (24). When the subtracted part is not normally distributed, as would happen if C is too small for the central limit theorem to be valid, then $q_{\text{rev}}^{\text{null}}$ is not normally distributed and the p-values are not accurate. If C is large, then the subtracted part and consequently, $q_{\text{rev}}^{\text{null}}$, remains normally distributed. In practical situations, only a few known covariates are used to correct the gene expression and in this limit of small C, the null model is not Gaussian.

In Fig. S2, we show that $q_{\text{rev}}^{\text{null}}$ is normally distributed for a given SNP and a gene expression matrix with $N = 581$ (from adipose subcutaneous tissue of GTEx). Setting the first seven singular

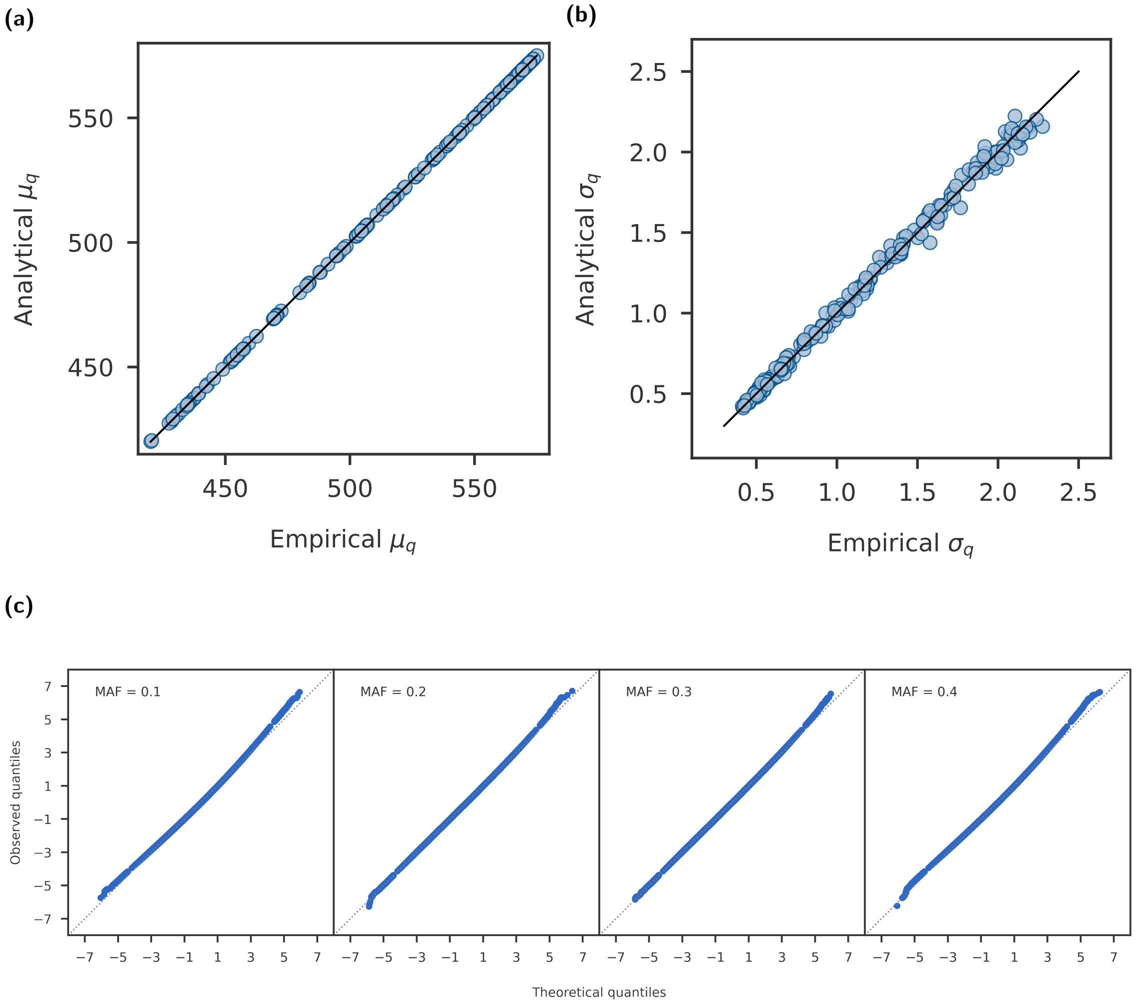

Fig. S1 | Validation of analytical calculation of mean and variance of RR-score null distribution. We compare the analytical calculation of **(a)** mean μ_q and **(b)** standard deviation σ_q of $q_{\text{rev}}^{\text{null}}$ with those obtained empirically for 200 randomly selected SNPs (GTEx data) with different minor allele frequencies. Each point represents a SNP. The empirical null distribution of q_{rev} for each SNP was created by permuting the genotype 500 times. **(c)** QQ plot of the empirical null distribution of q_{rev} for four representative SNPs with minor allele frequencies of 0.1, 0.2, 0.3 and 0.4. On the x-axis, we plot the theoretical quantiles of $\mathcal{N}(0, 1)$ and on the y-axis, we plot the observed quantiles of $(q_{\text{rev}} - \mu_q) / \sigma_q$. With a sampling size of 1e9 empirical permutations, the maximum observed quantiles correspond to p-values of 1.1e-10, 6.7e-11, 2.5e-12 and 2.7-11 respectively for the four SNPs. For all plots in this figure, we used the gene expression of adipose subcutaneous tissue from GTEx. For all q_{rev}, we used γ = 0.2.

values to zero (equivalent to correcting seven covariates) breaks the normal distribution of $q_{\text{rev}}^{\text{null}}$. However, if 200 singular values are set to zero, then the assumption of normal distribution of $q_{\text{rev}}^{\text{null}}$ becomes valid again. This illustrates the problem of using CCLM with few confounders in combination with Tejaas.

2.7 Comparison of RR-score with sequence kernel association test (SKAT)

The aggregation of weak signals from many covariates in the RR-score of Tejaas is reminiscent of methods developed for finding rare genetic variants y_i associated with a trait x that aggregate the effects of many rare variants in an entire locus or gene, such as the burden test [2] or the

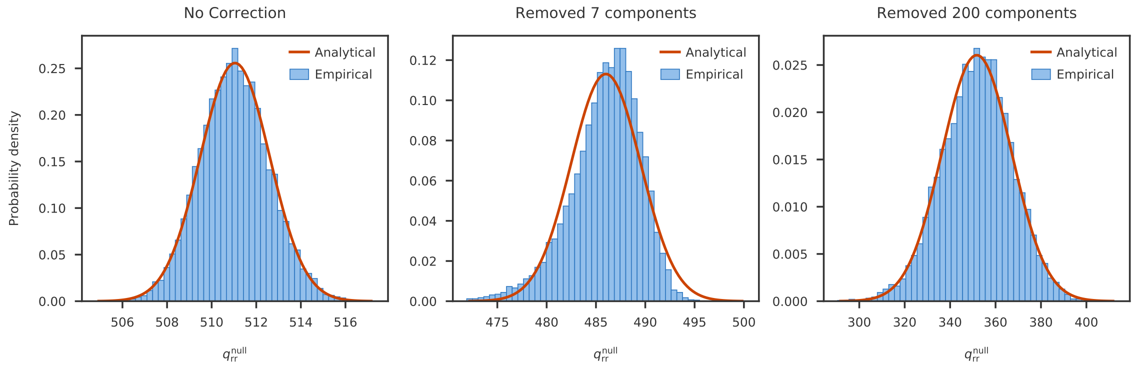

Fig. S2 | Effect of covariate correction on null model. We calculate the null model empirically for a SNP with minor allele frequency (MAF) 0.1 using the gene expression from adipose subcutaneous tissue (581 samples and 15763 genes) of GTEx. The blue histogram is the null distribution calculated empirically and the solid red line shows the analytically approximated normal distribution $\mathcal{N}(\mu_q, \sigma_q^2)$. Our analytical model is indeed valid without any covariate correction (left panel). When the gene expression is corrected for a few covariates, then the normal approximation breaks down as explained in the text (center panel). However, when a large number of singular values are removed, then the central limit theorem becomes valid again (right panel).

sequence kernel association test (SKAT) [3, 4].

Their test statistics take a similar form to that of Tejaas. All three can be written as

$$Q(\mathbf{x}) = \mathbf{x}^\mathsf{T} \mathbf{Y} \mathbf{R} \mathbf{Y}^\mathsf{T} \mathbf{x} \tag{26}$$

with $\mathbf{R} = (\sigma^2/\gamma^2\mathbb{I} + \mathbf{Y}^\mathsf{T}\mathbf{Y})^{-1}$ for Tejaas (Eq. (20)), $\mathbf{R} = \mathbf{1}\mathbf{1}^\mathsf{T}$ for the burden test and $\mathbf{R} = \mathbb{I}$ for SKAT – or, in the weighted version with diagonal weight matrix $\mathbf{\Pi} = \text{diag}(\pi_1, \ldots, \pi_P)$, $\mathbf{R} = \mathbf{\Pi}\,\mathbf{1}\mathbf{1}^\mathsf{T}\,\mathbf{\Pi}$ and $\mathbf{R} = \mathbf{\Pi}^2$, respectively (where $\mathbf{1}^\mathsf{T} := (1, \ldots, 1)$).

For $\gamma \longrightarrow 0$, RR-score of Tejaas tends towards the unweighted SKAT statistic. However, burden and SKAT are classical hypothesis tests, testing whether to reject the null hypothesis $\boldsymbol{\beta} = \mathbf{0}$. In contrast, Tejaas uses Bayesian model comparison, comparing the null model $\boldsymbol{\beta} = 0$ with the alternative trans-eQTL model $\boldsymbol{\beta} \neq \mathbf{0}$ (Eq. (15)) while integrating out the unknown effect strengths $\boldsymbol{\beta}$ (Eq. (17)). Since the trans-eQTL model includes the expected scale of effect sizes γ via its prior $p(\boldsymbol{\beta}) = \mathcal{N}\left(\boldsymbol{\beta} \mid 0, \gamma^2\right)$, the test statistic of Tejaas depends on this scale. As can be seen in Eq. (23), the multiplication with $\mathbf{R}$ leads to a saturation of the effects of principle components of $\mathbf{Y}$ with singular values larger than σ/γ.

Furthermore, whereas the burden test and SKAT commonly assume the response variable x under the null hypothesis to be identically and independently normally distributed, this assumption would be unsuitable for centered minor allele frequencies x_n. Instead, Tejaas assumes that patient indices n are randomly permuted under the null model (Sec. 2.6).

Whereas the SKAT statistic was *devised* because it was deemed useful to distinguish positives from negatives while being easy to compute, we used the Bayesian approach to *derive* a test statistic that should be reasonably good in distinguishing positives from negatives. Indeed, despite the similarity with the SKAT statistic, the equivalent TEJAAS kernel contains a correction $\mathbf{R}$ for the correlation between covariates, which does not appear in SKAT and related methods. This correction has its origin in the non-null model, for which regression coefficients deviating substantially from 0 are considered.

Once derived, we strove to make the TEJAAS statistic fast to compute by means of analytically calculating the first and second moments (Appendix 1). Its time complexity is dominated by the inversion of the matrix $\sigma^2/\gamma^2\mathbb{I} + \mathbf{Y}^\mathsf{T}\mathbf{Y}$, which takes $O(NG \min\{G, N\})$, while p-values are computed in $O(N^2)$ (Appendix 1). In comparison, SKAT scores are computed in $O(NG)$, while

the computation of the p-values require the inversion of an $N \times N$ matrix, taking time $O(N^3)$.

2.8 Choice of the regularizer

The standard deviation γ of the normal prior is not learned from the data, but is chosen empirically. It is important to choose γ such that it avoids overfitting and also restricting the regression coefficients too much..

In the limit of large γ, the normal approximation to q_{rev} will break down. To see this, we first observe that from Eq. (24)

$$q_{\text{rev}} < \frac{1}{\sigma^2} \sum_{k=1}^{K} (\mathbf{u}_k^\mathsf{T} \mathbf{x})^2 = \frac{\|\mathbf{x}\|^2}{\sigma^2} = 1. \tag{27}$$

Therefore, as σ^2/γ^2 becomes smaller than most squared singular values $\{s_k^2 : 1 \le k \le K\}$, q_{rev} will approach 1. The distribution of q_{rev} under the permutation null model will therefore become extremely skewed with an abrupt drop towards 1. Such a distribution will not be approximated well by a Gaussian, but luckily this situation corresponds to the irrelevant regime of extreme overfitting. This can be seen by noting that the prediction of $\mathbf{x}$ at the maximum likelihood solution $\hat{\boldsymbol{\beta}} = \boldsymbol{\Lambda}^{-1}\mathbf{Y}\mathbf{x} = (\mathbf{Y}\mathbf{Y}^\mathsf{T} + (\sigma^2/\gamma^2)\,\mathbb{I}_G)^{-1}\mathbf{Y}\mathbf{x}$ is $\mathbf{Y}^\mathsf{T}\hat{\boldsymbol{\beta}} \approx \mathbf{x}$, which means that the regression results in a perfect prediction on the training samples, in other words, it results in complete overfitting.

At the other extreme, when γ gets so *small* that $\sigma^2/\gamma^2 \gg \max\{s_k^2 : 1 \le k \le K\}$, we can approximate

$$\begin{aligned} q_{\text{rev}} &\approx \frac{\gamma^2}{\sigma^4} \sum_{k=1}^{K} s_k^2 \left(\mathbf{u}_k^\mathsf{T} \mathbf{x}\right)^2 \\ &= \frac{\gamma^2}{\sigma^4} \mathbf{x}^\mathsf{T} \left(\mathbf{Y}^\mathsf{T}\mathbf{Y}\right) \mathbf{x} \\ &= N \frac{\gamma^2}{\sigma^4} \mathbf{x}^\mathsf{T} \boldsymbol{\Sigma} \mathbf{x} \end{aligned} \tag{28}$$

where $\boldsymbol{\Sigma}$ is the $N \times N$ sample covariance matrix. Since our gene expression matrix $\mathbf{Y}$ is normalized, the diagonal elements of $\boldsymbol{\Sigma}$ should be equal to 1, whereas the off-diagonal elements will be more or less randomly distributed around 0. Intuitively, we expect that in this regime q_{rev} will be will be too restrictive for the model and lead to false signals even with chance correlations of a single gene with a genotype.

Non-Gaussian parameter. From Eq. (25), the $q_{\text{rev}}^{\text{null}}$ should have a normal distribution and the standardized $q_{\text{rev}}^{\text{null}}$ should have a standard normal distribution,

$$P\left(\frac{q_{\text{rev}}^{\text{null}} - \mu_q}{\sigma_q}\right) = \mathcal{N}(0, 1) \ . \tag{29}$$

The kurtosis of a standard normal distribution is 3. We use this property to define a non-Gaussian parameter (α) to measure the deviation of the distribution from a standard normal distribution,

$$\alpha(\gamma) = \left| \frac{\left\langle f(q;\gamma)^4 \right\rangle}{3} - 1 \right| , \tag{30}$$

where $f(q;\gamma) = \left(q_{\text{rev}}^{\text{null}} - \mu_q\right)/\sigma_q$. Given a gene expression matrix, we simulated genotypes for 5000 SNPs and calculated $\alpha(\gamma)$ for different values of γ. We recommend choosing γ_{opt} such that

$\gamma_{\text{opt}} \geq \arg\min_{\gamma} \alpha(\gamma)$. Ideally, we also want a high value for the standard deviation of σ_q to ensure a broad distribution of $q_{\text{rev}}^{\text{null}}$.

2.9 Target genes

There are two separate tasks in the analysis of trans-eQTLs: (i) identifying the trans-eQTL SNPs, and (ii) identifying their target genes. However, the second task is subordinate in the sense that if trans eQTLs cannot be predicted correctly there is no use in predicting target genes. Conversely, if the trans-eQTLs can be predicted correctly then some complementary method can be used for predicting their target genes.

Reverse Regression offers evidence for the presence of a trans-eQTL without identifying *which* genes are targeted. Hence, we do not get the set of target genes from Reverse Regression. In fact, multiple regression with L_2 penalty (ridge regression) is not optimal for variable selection.

Therefore, in Tejaas, we included a single SNP-gene pairwise regression to find a set of candidate genes as targets for each predicted trans-eQTL. Under the hood, our software performs the following tasks:

- perform reverse regression to rank all trans-eQTL SNPs and select the top trans-eQTLs below a cutoff (default 0.001), can be specified by `--psnpthres`.
- perform single SNP-gene linear regression for all preselected trans-eQTL SNPs and estimate the Benjamini-Hochberg (BH) false discovery rate for the association of all genes with the candidate trans-eQTL SNP.

The output of Tejaas contains both the trans-eQTL SNPs ranked by p-values and all SNP-gene pairs below a p-value cutoff (`--pgenethres`, default 0.01) with their corresponding BH adjusted p-values at 50% FDR (`--fdrgenethres`).

As discussed below (Sec. 3), reverse regression works better with KNN correction while pairwise regression (for target gene discovery) works better with conventional covariate-corrected gene expression. To get the best of both worlds, our software accepts two separate input options for gene expression files: (1) `--gx` to provide the raw gene expression file for KNN correction and trans-eQTL discovery, and (2) `--gxcorr` to provide the covariate-corrected gene expression file for single SNP-gene pair regression to discover target genes.

2.10 Cis-masking

In Tejaas, we are interested to find long-range SNP-gene interactions. However, the strong effect size of the cis-eQTLs can sometimes lead to high q_{rev}. To avoid wrongly identifying cis-eQTLs as trans-eQTLs, we introduced an option in our software to exclude all genes in the vicinity of each SNP. We call this procedure 'cis-masking'. It can be invoked with the flag `--cismask`. The width of the vicinity can be specified using the option `--window`.

To implement cis-masking, we calculated the SVD of the expression matrix (see Eq. (21)) after removing (masking out) the genes that occur within ±1Mb (or any distance specified by `--window`) from the SNP position. Doing this for every SNP would be computationally expensive as it would involve $\sim 10^7$ matrix inversions (once for each SVD). But since many SNPs generally have identical genes in the vicinity, we group them and calculate the SVD once for each group, which brings down the number of SVD calculations to $\sim 10^4$.

2.11 Computational requirements

To determine run times and memory requirements of Tejaas, we used the adipose subcutaneous tissue expression from GTEx, which contains a total of 15673 genes for 581 samples. We used the

GTEx genotype for chromosome 1. All tests were run on a node with 2× Intel Xeon CPU E5-2640 v3 @ 2.60GHz with 8 physical cores each and 128Gb of RAM. We subsampled the expression matrix as well as the number of SNPs used to run Tejaas to evaluate different possible scenarios.

The most expensive step for Tejaas is the SVD decomposition of the gene expression matrix. The number of times the SVD decomposition is performed depends directly on the number of SNPs selected. While using `--cis-masking` (Sec. 2.10), different SNPs will have different cis-genes that are required to be masked out and hence, a new SVD decomposition is required. Run times increase with the number of SNPs (Fig. S3a, left panel), since the number of cismasks needed (top x-axis) is proportional to the number of SNPs (bottom x-axis). Memory usage increases with the number of SNPs and samples, since the dosage matrix is being held in memory.

We implemented Tejaas with an option to run Tejaas on a multicore server using MPI parallelization. Fig. S3b shows how run times decrease with the number of cores used. Total memory usage, as expected, increases with the number of cores. In the designed parallelization scheme, each core will compute the SVD decomposition for a given cismask. With an increase in the number of cores, the expression matrix needs to be copied to each of them for processing and hence the memory usage scales proportionally.

3 Confounder correction

Gene expression measurements are notorious for being dominated by strong confounding effects that can arise from technical details of RNA recovery, conservation, and sequencing or from environmental and biological factors such as age, gender, diseases, nutrition and lifestyle, drug regimes etc. The subtle effects of trans-eQTLs are at risk of being drowned out by the strong systematic noise from confounding effects.

In this section, we first discuss the standard confounder correction method for eQTL analyses. Then, we introduce our K-nearest neighbor correction that is specially suitable for use with Tejaas.

3.1 Residuals from multiple linear regression

Given a matrix $\mathbf{C} \in \mathbb{R}^{C\times N}$ of C covariates and N samples, a multiple linear regression is performed for the gth gene expression $\mathbf{y}_g$,

$$\mathbf{y}_g = \boldsymbol{\alpha}_g \mathbf{C} + \boldsymbol{\xi}_g\,, \tag{31}$$

where $\boldsymbol{\alpha}_g \in \mathbb{R}^C$ is a vector of effect sizes of the covariates for gene g. The estimate $\widehat{\boldsymbol{\alpha}_g}$ of the effect sizes from the multiple linear regression is then used to calculate the expression residuals $\mathbf{y}'_g = \mathbf{y}_g - \widehat{\boldsymbol{\alpha}_g}\mathbf{C}$ which are linearly uncorrelated with the covariates. The residual $\mathbf{y}'_g$ is used as the corrected gene expression for downstream analyses. In this manuscript, we denote this correction as 'CCLM'.

This is a simple and effective method to correct gene expression confounders and is routinely used to discover biologically significant eQTLs. CCLM can be used for known covariates such as age, gender, etc. as well as other hidden covariates inferred using PEER [5] or other similar methods. However, if $\operatorname{rank}(\mathbf{Y}) = N$, then it can be shown that for the residual matrix, $\operatorname{rank}(\mathbf{Y}') \leq N - C$. Therefore, as noted in Sec. 2.6, this may compromise the normality of the distribution of $q_{\text{rev}}^{\text{null}}$. Hence, the CCLM correction is problematic to use with Tejaas.

3.2 K-nearest neighbor (KNN) correction

For the KNN correction, we assume that confounding effects dominate the gene expression. We expect that the expression levels of the genes of K nearest neighbors (NN_n^K) of each sample n will be affected by the same dominant confounder variables. In other words, if the samples are close to

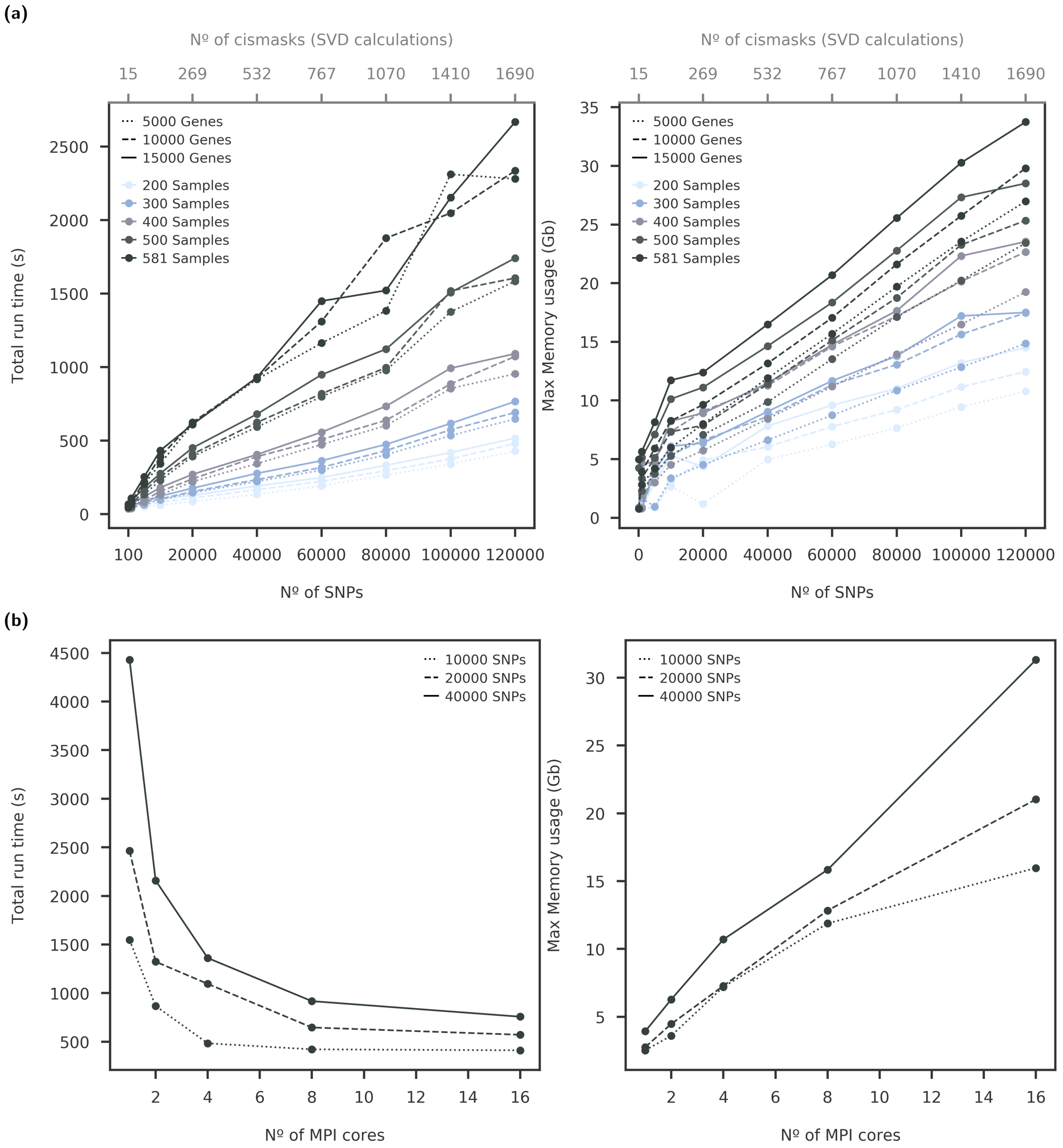

Fig. S3 | Computational time and memory requirements of Tejaas. (a) Total run time and maximum memory usage as a function of SNPs are shown on the left and right panels, respectively. All runs of Tejaas used 8 CPU cores for parallelization. The top x-axis reflects how many cismasks are required in chromosome 1 for a given number of SNPs. **(b)** Total run time and maximum memory usage as a function of the number of CPU cores. All runs of Tejaas used 15673 genes for 581 samples.

one another in the expression space, we expect them to be close to one another in the confounder space. Hence, we can correct at least a good part of the confounding effects by centering the expression $\mathbf{y}_n$ and genotype $\mathbf{x}_n$ of sample n using the K samples whose gene expressions are the most similar to that of sample n,

$$\mathbf{y}_n \leftarrow \mathbf{y}_n - \frac{1}{K} \sum_{m \in \mathrm{NN}_n^K} \mathbf{y}_m \tag{32}$$

$$\mathbf{x}_n \leftarrow \mathbf{x}_n - \frac{1}{K} \sum_{m \in \mathrm{NN}_n^K} \mathbf{x}_m \,. \tag{33}$$

The nearest neighbors NN_n^K are calculated using the denoised Euclidean distances between gene expression vectors. To reduce noise in the distance calculation, we remove all but the leading M principle components of the singular value decomposition of the expression matrix $\mathbf{Y}$. After subtracting the mean gene expressions and genotype vectors of the K nearest neighbors, we center the resulting corrected gene expression and genotype vectors again. Since KNN does not reduce the rank of the gene expression matrix, it works well with Tejaas. As shown in the QQ plots of Fig. S4, the empirical null distribution indeed follows the normal distribution.

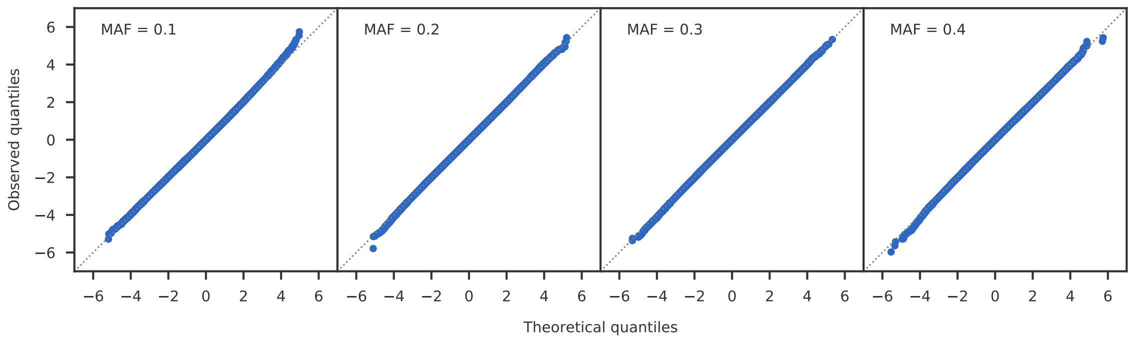

Fig. S4 | Normal approximation of permuted q_{rev} remains valid after KNN correction. Using the gene expression of adipose subcutaneous tissue from GTEx, we applied the KNN correction with $K = 30$. Here, we show that the empirical null distribution of q_{rev} follows the normal distribution for four representative SNPs with minor allele frequencies (MAF) of 0.1, 0.2, 0.3 and 0.4. In each panel, we show the theoretical quantiles of $\mathcal{N}(0, 1)$ on the x-axis and the observed quantiles of $(q_{\mathrm{rev}} - \mu_q) / \sigma_q$ on the y-axis. The maximum quantiles correspond to p-values of 4.6e-9, 2.8e-8, 4.7e-8 and 2.9e-8 for the four SNPs respectively. For all plots, q_{rev} was calculated with $\gamma = 0.2$.

The choice of K should be such that it captures the locally varying effects of the confounders. As shown later in Fig. S10, too small a value of K would lead to excessive statistical noise, while increasing K too much will remove too little of the confounding effects as these start to average out within the K neighbors of each data point.

In Fig. S5, we show how KNN correction removes the strong dissimilarity between samples in the data. Before KNN correction (Fig. S5a), the samples are strongly dissimilar. We could observe several sample groups which are neighbors in the expression space, probably due to external confounders. Although the samples were clustered in the expression space, we observed distinctive patches in the genotype space. This happens because several confounders (such as population substructure) affect both genotype and gene expression, leading to unwanted correlation between the first principal component of the genotype and the expression levels. We could correct for these confounders by centering both $\mathbf{x}_n$ and $\mathbf{y}_n$ in the KNN method (Fig. S5b).

The KNN correction has several benefits in comparison to CCLM and other linear corrections. First, it can correct out non-linear confounding effects, so, in contrast to the CCLM correction, it should also work when the confounding effects are not well approximated by linear, additive effects. Second, since it is non-parametric (except for K), overfitting does not typically occur [6, 7]. Third and most importantly, it does not require the confounders to be known. Therefore, it can be used for removing hidden confounders, which is often the case with real data.

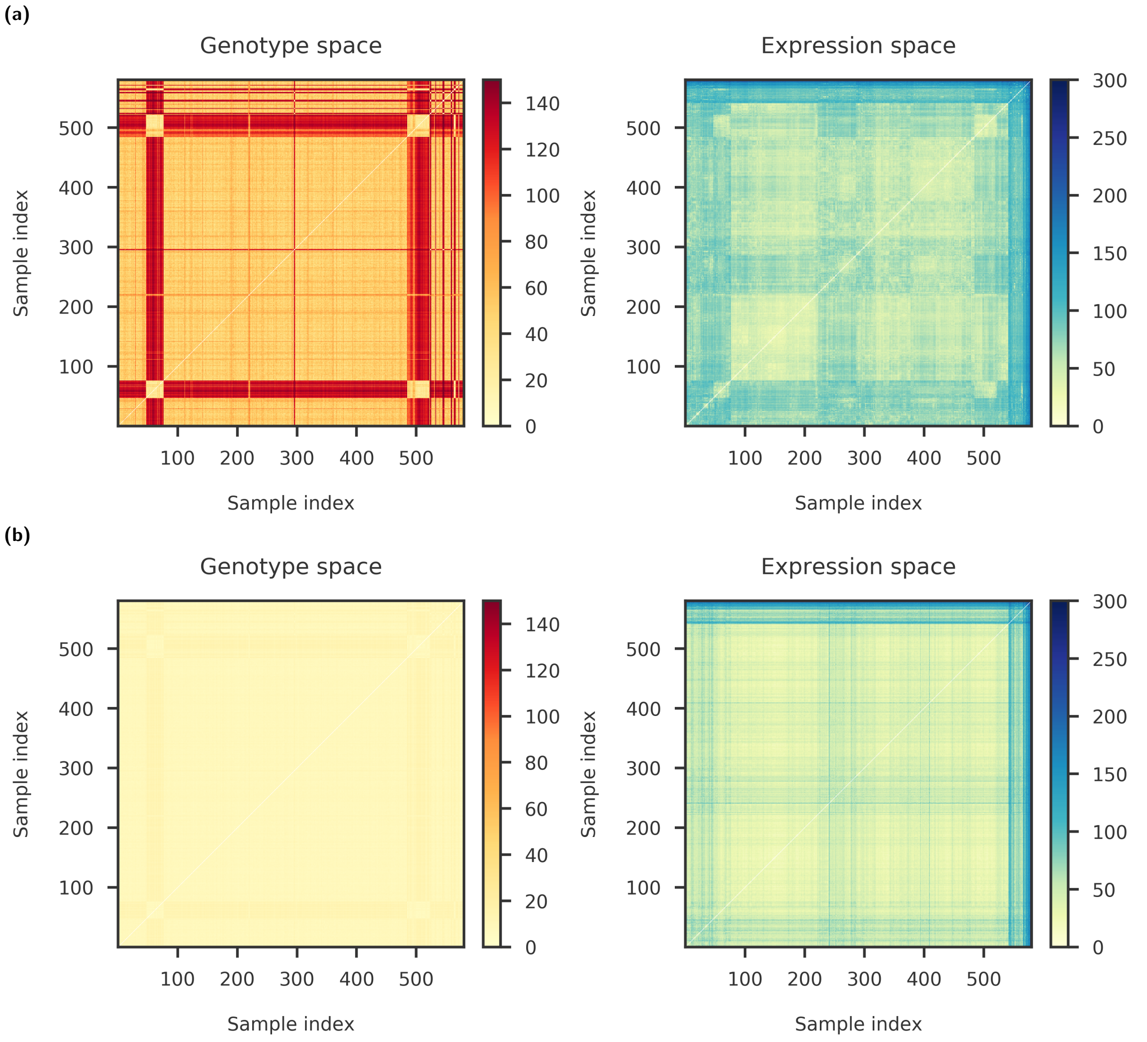

Fig. S5 | Distance between samples before and after KNN correction. Heatmaps with color indicating the distance between samples (N = 581) in both the genotype space (using LD pruned genotype from GTEx) and the expression space (using adipose subcutaneous tissue from GTEx): **(a)** Before KNN correction, and **(b)** after KNN correction. The samples are clustered using their distance in the expression space before KNN correction. For the example shown here, we reduced the expression matrix to 30 dimensions (M = 30) and considered 30 nearest neighbors (K = 30) for centering the genotype and expression.

4 Trans-eQTL simulation

This section describes details of a simulation study that evaluates the power of our method to find *trans* effects in gene expression data. We followed the strategy of Hore *et al.* for the simulation, as described in the Supplementary section of their article [8]. We discuss their simulation details below, partly verbatim. Any difference with their strategy is explicitly noted. We also compare the

4.1 Data simulation

Simulated data consisted of genotype and gene expression for $N = 450$ individuals to closely resemble the sample size of the Genotype Tissue Expression (GTEx) project [9–11]. We simulated the gene expression data for $G = 12\,639$ genes, containing non-genetic signals (background correlation estimated from the GTEx tissues and confounding factors) and genetic signals (*cis*

and *trans* effects). Following the strategy of Hore *et al.* [8], we assumed that each gene contained only one SNP; this simplified case is equivalent to assuming that there is at most one cis-eQTL for each gene.

4.1.1 Genotypes

We used the real genotype data from the GTEx individuals, while Hore *et al.*used simulated genotypes. After pre-filtering of the GTEx genotype, we randomly sampled $I = 12\,639$ SNPs (corresponding to $G = 12\,639$ genes) from the genotype data of 450 individuals. From the I SNPs sampled, we randomly selected $I_{\text{cis}} = 800$ SNPs to be cis-eQTLs. From these cis-eQTLs, we selected a subset $I_{\text{trans}} = 30$ SNPs to be trans-eQTLs. These trans-eQTLs were originally cis-eQTLs associated with a nearby gene, which in turn regulated the expression of other distant genes. All SNPs were sampled independently from the genotype. Let $\mathbf{X} \in \mathbb{R}^{I \times N}$ be the sampled matrix of genotypes.

4.1.2 Gene expression

Let $\mathbf{Y} \in \mathbb{R}^{G \times N}$ be the matrix of simulated gene expression data. The data was simulated in stages. First, we generated the non-genetic component of all genes using background correlation (noise) and confounding factors. Second, we added the cis effects and finally, we added the *trans* component on the target genes of the trans-eQTLs.

Noise. Hore *et al.*used heteroscedastic background noise. However, to replicate the underlying correlation of the gene expressions in the GTEx samples, we created a Gaussian noise with a covariance matrix obtained from the gene expressions in the artery aorta tissue of GTEx. Let $\mathbf{Y}_{\text{as}}$ be the observed gene expression in GTEx samples. We decomposed the covariance matrix of $\mathbf{Y}_{\text{as}}$ such that

$$\text{Cov}\left(\mathbf{Y}_{\text{as}}\right) = \mathbf{Q}\boldsymbol{\Lambda}\mathbf{Q}^{\mathsf{T}} \tag{34}$$

We defined the noise as

$$\mathbf{Y}^{\text{noise}} = \mathbf{Q}\sqrt{\boldsymbol{\Lambda}}\mathbf{Z} \tag{35}$$

where $\mathbf{Z}$ is a $G \times N$ matrix, with every row sampled from a normal distribution with zero mean and unit variance. Note that $\text{Cov}\left(\mathbf{Y}_{\text{noise}}\right) = \text{Cov}\left(\mathbf{Y}_{\text{as}}\right)$ and therefore $\mathbf{Y}^{\text{noise}}$ retains the background correlation structure of the adipose subcutaneous tissue.

Confounding factors. We generated $C = 10$ confounding factors, of which $C_{\text{pop}} = 3$ were considered to be due to population substructure. Let $\mathbf{W}$ be the $C \times N$ matrix of confounding factors. The three population substructure confounders ($\mathbf{W}^{\text{pop}}$) were set to be the first, second and third principal components of the corresponding genotype matrix. The remaining $C_{\text{ind}} = C - C_{\text{pop}}$ independent confounders ($\mathbf{W}^{\text{ind}}$) were sampled from a standard normal distribution,

$$\mathbf{W}_i^{\text{ind}} \sim \mathcal{N}\left(0, 1\right) \tag{36}$$

Each confounding factor was assumed to be affecting only a few target genes. Hence, the effect size β_{cg} for the cth confounding factor on the gth gene was sampled with a sparsity α_c,

$$\beta_{cg} \sim \alpha_c \, \mathcal{N}\left(0, \sigma_c^2\right) + \left(1 - \alpha_c\right)\delta_0, \;\; \alpha_c \sim \text{Beta}\left(2, 5\right) \tag{37}$$

to give us the coefficient matrix $\boldsymbol{\beta}$ of size $C \times G$. We used $\sigma_c = 1.0$ unless otherwise mentioned. Finally, we obtained the effect of confounding factors on the gene expression using an additive

model,

$$\mathbf{Y}^{\text{cf}} = \boldsymbol{\beta}^{\intercal}\mathbf{W} \tag{38}$$

Cis effects. In this simulation framework, every gene spatially overlaps with a corresponding SNP in the genotype, *i.e.*, we have I (= G) SNP-gene pairs. If the SNP in the ith position has a cis effect ($i \in I_{\text{cis}}$), then the expression of the gene i is modified with a strength α_i,

$$\mathbf{Y}_i^{\text{cis}} = \alpha_i \mathbf{X}_i \tag{39}$$

where the direction of the effect size is random, *i.e.*, $\text{sgn}(\alpha_i)$ is assigned a value of -1 or $+1$ randomly. The strength of the cis effect, *i.e.*, $|\alpha_i|$ depends on whether the SNP also has a trans effect or not and is given by

$$|\alpha_i| = \begin{cases} 0.6, & \text{if SNP } i \text{ also acts as a trans-eQTL } (\in I_{\text{trans}}) \\ \text{Gamma}(4, 0.1), & \text{otherwise.} \end{cases} \tag{40}$$

For all the remaining SNP-gene pairs ($i \notin I_{\text{cis}}$) with no cis effects, $\mathbf{Y}_i^{\text{cis}} = \mathbf{0}$.

Combining noise, confounding factors and cis effects. We obtained a temporary set of expression levels for all genes by additively combining the noise, the effect of confounding factors and the effect of cis-eQTLs,

$$\mathbf{Y}^{\text{tmp}} = \mathbf{Y}^{\text{noise}} + \mathbf{Y}^{\text{cf}} + \mathbf{Y}^{\text{cis}} \tag{41}$$

Note that the $\mathbf{Y}^{\text{cis}}$ has non-zero values only for the targets of $I_{\text{cis}} = 800$ cis-eQTLs, and has zero values for all remaining genes.

Trans effects. For simulating the trans effects, Hore *et al.*assumed that the trans-eQTLs regulated a nearby gene (via cis effect) and this cis target gene was a transcription factor (TF) and regulated multiple target genes downstream (excluding other TF genes; could be any other gene including other cis target genes). This ensured that the trans-eQTLs were indirectly associated with the target genes with practically low effect sizes.

Let M_{trans} be the number of target genes regulated by the TF and the target genes of every TF were selected at random. Let S_g be the set of TFs which regulate the expression of the gth gene and the trans effect on this gene was simulated as,

$$\mathbf{Y}_g^{\text{trans}} = \sum_{j \in S_g} \beta_{gj} \mathbf{Y}_j^{\text{tmp}}, \quad \text{where} \tag{42}$$

$$\left|\beta_{gj}\right| \sim \text{Gamma}\left(\psi^{\text{trans}}, 0.02\right). \tag{43}$$

β_{gj} was the relative effect of TF j on the gene g. If a gene was not a target for any of the $I_{\text{trans}} = 30$ TFs, then $\mathbf{Y}_g^{\text{trans}} = \mathbf{0}$.

Combining all of the data. Finally, the contributions from the trans-eQTLs were incorporated to create a final set of simulated expression levels,

$$\mathbf{Y} = \mathbf{Y}^{\text{tmp}} + \mathbf{Y}^{\text{trans}} \tag{44}$$

and was used for subsequent analyses.

4.1.3 Choice of simulation parameters

As described above, there are several parameters which can be tuned in the simulation to generate a wide range of confounding effects, cis effects and trans effects. Three parameters determine the strength of a simulated trans-effect:

1. Effect of the cis-eQTL on the TF (α_i for $i \in I_{\text{cis}}$).
2. Effect of the TF on the target genes. This is determined by ψ^{trans} and the mean effect of the TFs on the target genes is given by $\left\langle \left|\beta_{gj}\right| \right\rangle = 0.02\psi^{\text{trans}}$
3. The number of target genes (M_{trans}).

Hore *et al.* [8] selected these parameters with reference to the KLF14 trans signal [12] so that the signal strengths were similar to the real data. For our simulation, we chose the same values for these parameters, *i.e.*, $\alpha_i = 0.6$ for $i \in I_{\text{cis}}$, $\psi^{\text{trans}} = 20$ and $M_{\text{trans}} = 150$. We also explored several other choices of parameters by reducing the strength of trans effects, particularly by tuning ψ^{trans} (= 5, 10 and 20) and M_{trans} (= 50 and 100) as shown in Fig. 2 of the main manuscript. In order to get an idea of how realistic the simulations are, we looked at the effect sizes of the trans-eQTLs and the estimated expression heritabilities contributed by the trans-eQTLs.

Effect sizes. The expression of the gth gene is given by

$$\begin{aligned} \mathbf{Y}_g &= \mathbf{Y}_g^{\text{noise}} + \mathbf{Y}_g^{\text{cf}} + \mathbf{Y}_g^{\text{cis}} + \sum_{j \in S_g} \beta_{gj} \left(\mathbf{Y}_j^{\text{noise}} + \mathbf{Y}_j^{\text{cf}} + \mathbf{Y}_j^{\text{cis}} \right) \\ &= \mathbf{Y}_g^{\text{noise}} + \mathbf{Y}_g^{\text{cf}} + \mathbf{Y}_g^{\text{cis}} + \sum_{j \in S_g} \beta_{gj} \left(\mathbf{Y}_j^{\text{noise}} + \mathbf{Y}_j^{\text{cf}} \right) + \widetilde{\mathbf{Y}}_g^{\text{trans}} \end{aligned} \tag{45}$$

where we defined

$$\widetilde{\mathbf{Y}}_g^{\text{trans}} := \sum_{j \in S_g} \beta_{gj} \mathbf{Y}_j^{\text{cis}} = \sum_{j \in S_g} \beta_{gj} \alpha_j \mathbf{X}_j \tag{46}$$

Hence the effect of an individual SNP j on gene g in an additive model,

$$\mathbf{Y}_g = \gamma_{gj} \mathbf{X}_j + e_g \tag{47}$$

will be given by $\gamma_{gj} = \beta_{gj}\alpha_j$ and all other terms will be included in the error term e_g. Simple linear regression (methods like MatrixEQTL) calculates an estimate $\widehat{\gamma}_{gj}$ for the coefficient. The standard error for the estimated coefficient will be given by

$$\text{se}\left(\widehat{\gamma}_{gj}\right) = \sqrt{\widehat{\sigma}_{eg}^2 \left(\mathbf{X}_j^{\mathsf{T}} \mathbf{X}_j \right)^{-1}}, \tag{48}$$

where the residual can be obtained as $\widehat{\sigma}_{eg}^2 = \text{Var}\left(\mathbf{Y}_g\right) - \widehat{\gamma}_{gj}^2 \text{Var}\left(\mathbf{X}_j\right)$. For comparison across multiple SNPs and studies the effect size $\widehat{\gamma}_{gj}$ is scaled by the standard error to obtain the Z score,

$$Z = \frac{\widehat{\gamma}}{\text{se}\left(\widehat{\gamma}\right)} \tag{49}$$

Since we planted the trans-eQTLs in simulations, we know the "true" values of the coefficients, *i.e.*, $\widehat{\gamma}_{gj} = \gamma_{gj}$, and can calculate the Z scores for all SNP-gene pairs where $\gamma_{gj} > 0$. We show the distribution of the $|Z|$ scores in Fig. S6a and compare it with the $|Z|$ scores of the most significant eQTLs predicted by GTEx [13]. It should be noted that the predicted cis-eQTLs and trans-eQTLs from GTEx do not represent the background distribution because there could be many other

cis or trans-eQTLs which might have not been predicted. However, the distributions give an idea of the effect sizes that are required to be considered significant by simple regression. The distribution of cis-eQTL $|Z|$ scores overlap with that of predicted cis-eQTLs in GTEx, indicating that the cis-eQTL effect sizes are realistic and are expected to be predicted by standard eQTL mapping. The effect size of the simulated trans-eQTLs are much weaker compared to the 156 significant trans-eQTL and target gene pairs discovered by the GTEx consortium. Consistent with our expectations, (i) the trans-eQTLs have weaker effect sizes compared to cis-eQTLs and (ii) they cannot be discovered by standard eQTL mapping with the given sample size. However, it remains unknown how much weaker the trans-eQTLs really are.

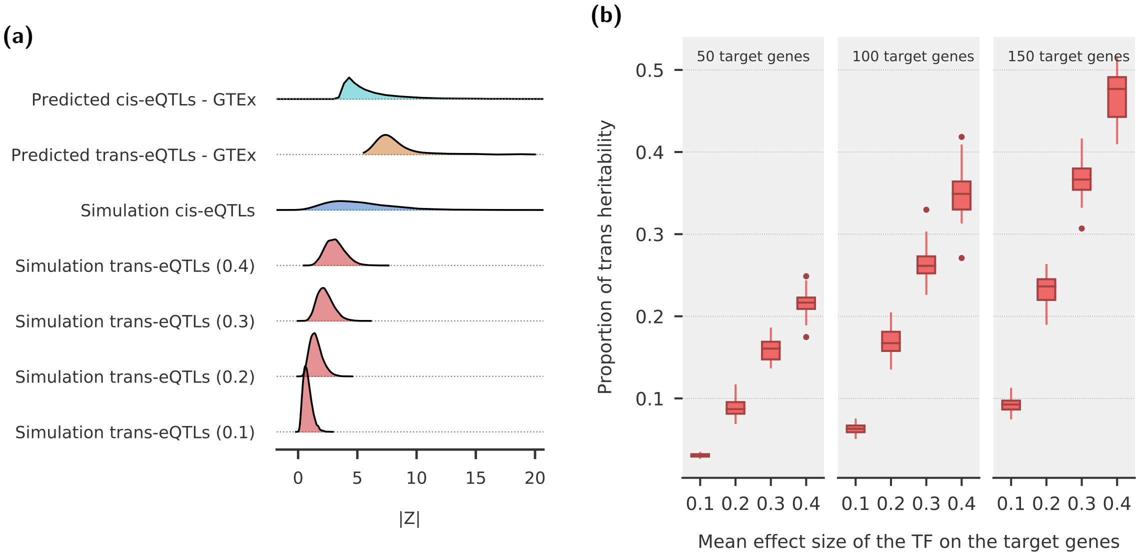

Fig. S6 | Calibration of the choice of simulation parameters. (a) We show the normalized distribution of |Z| scores for the planted trans-eQTLs and cis-eQTLs in simulation (legends on y-axis). We performed simulations with four different effect size distributions of the trans-eQTLs, characterized by the mean effect size of the TFs on the target genes (0.1, 0.2, 0.3 and 0.4 respectively, shown in parentheses of the legends). We compare these distributions with the |Z| scores of genome-wide significant cis-eQTLs and trans-eQTLs predicted from the GTEx data. Consistent with our expectations, the simulated trans-eQTLs have much weaker effect sizes compared to cis-eQTLs and are too weak to be significant using standard trans-eQTL mapping. **(b)** The proportion of expression heritability contributed by trans-eQTLs in the simulations with different sets of parameters.

Heritability. The expression heritability (narrow-sense) and their cis and trans components for the gth gene can be calculated as

$$h_g^2 = \frac{\text{Var}\left(\mathbf{Y}_g^{\text{cis}} + \widetilde{\mathbf{Y}}_g^{\text{trans}}\right)}{\text{Var}\left(\mathbf{Y}_g\right)} \tag{50}$$

$$= \frac{\text{Var}\left(\mathbf{Y}_g^{\text{cis}}\right) + \text{Var}\left(\widetilde{\mathbf{Y}}_g^{\text{trans}}\right) + 2\,\text{Cov}\left(\mathbf{Y}_g^{\text{cis}}, \widetilde{\mathbf{Y}}_g^{\text{trans}}\right)}{\text{Var}\left(\mathbf{Y}_g\right)} \tag{51}$$

$$h_{g,\text{cis}}^2 = \frac{\text{Var}\left(\mathbf{Y}_g^{\text{cis}}\right)}{\text{Var}\left(\mathbf{Y}_g\right)} = \frac{\alpha_g^2\,\text{Var}\left(\mathbf{X}_g\right)}{\text{Var}\left(\mathbf{Y}_g\right)} \tag{52}$$

$$h^2_{g,\text{trans}} = \frac{\operatorname{Var}\left(\widetilde{\mathbf{Y}}^{\text{trans}}_g\right)}{\operatorname{Var}\left(\mathbf{Y}_g\right)} = \frac{\sum_{j\in S_g} \beta^2_{gj}\alpha^2_j \operatorname{Var}\left(\mathbf{X}_j\right) + 2\sum_{j\in S_g}\sum_{k=1}^{j-1}\beta_{gj}\beta_{gk}\alpha_j\alpha_k \operatorname{Cov}\left(\mathbf{X}_j,\mathbf{X}_k\right)}{\operatorname{Var}\left(\mathbf{Y}_g\right)}\,, \tag{53}$$

where the denominator $\operatorname{Var}\left(\mathbf{Y}_g\right)$ depends non-trivially on the correlated noise and other confounding effects:

$$\begin{aligned}\operatorname{Var}\left(\mathbf{Y}_g\right) = &\operatorname{Var}\left(\mathbf{Y}^{\text{noise}}_g\right) + \operatorname{Var}\left(\mathbf{Y}^{\text{cf}}_g\right) + \alpha^2_g \operatorname{Var}\left(\mathbf{X}_g\right)\\ &+ \sum_{j\in S_g}\beta^2_{gj}\operatorname{Var}\left(\mathbf{Y}^{\text{noise}}_j + \mathbf{Y}^{\text{cf}}_j\right) + \operatorname{Var}\left(\widetilde{\mathbf{Y}}^{\text{trans}}_g\right)\\ &+ 2\operatorname{Cov}\left(\mathbf{Y}^{\text{noise}}_g, \sum_{j\in S_g}\beta_{gj}\mathbf{Y}^{\text{noise}}_j\right) + 2\operatorname{Cov}\left(\mathbf{Y}^{\text{cf}}_g, \sum_{j\in S_g}\beta_{gj}\mathbf{Y}^{\text{cf}}_j\right)\\ &+ 2\operatorname{Cov}\left(\mathbf{Y}^{\text{cis}}_g, \widetilde{\mathbf{Y}}^{\text{trans}}_g\right)\,. \end{aligned} \tag{54}$$

where we have used

$$\operatorname{Cov}\left(\mathbf{Y}^{\text{noise}}_g, \mathbf{Y}^{\text{cf}}_{g'}\right) = 0 \tag{55}$$

$$\operatorname{Cov}\left(\mathbf{Y}^{\text{noise}}_g, \mathbf{Y}^{\text{cis}}_{g'}\right) = 0 \tag{56}$$

$$\operatorname{Cov}\left(\mathbf{Y}^{\text{cis}}_g, \mathbf{Y}^{\text{cf}}_{g'}\right) = 0\,. \tag{57}$$

We can assume that the last term $\operatorname{Cov}\left(\mathbf{Y}^{\text{cis}}_g, \widetilde{\mathbf{Y}}^{\text{trans}}_g\right) = 0$ because if gene g is targeted by both a cis-eQTL ($\mathbf{X}_g$) and a trans-eQTL ($\mathbf{X}_j, j \in S_g$), then the trans-eQTL must be distally separated from the cis-eQTL and therefore, probably not correlated so that $\operatorname{Cov}\left(\mathbf{X}_g, \mathbf{X}_j\right) = 0$. It follows that the last term in the numerator of Eq. (53) can also be assumed to be zero.

To obtain the cis and trans heritability, we empirically calculated $\operatorname{Var}\left(\mathbf{Y}_g\right)$ from the simulated expression data and calculated the numerators in Eq. (52) and (53) from the input effect sizes and sampled genotypes for the simulation. The mean cis and trans heritability over all genes can be obtained as,

$$h^2_{\text{cis}} = \frac{1}{G}\sum_{g\in G_{\text{cis}}} h^2_{g,\text{cis}} \tag{58}$$

$$h^2_{\text{trans}} = \frac{1}{G}\sum_{g\in G_{\text{trans}}} h^2_{g,\text{trans}} \tag{59}$$

where G_{cis} are the genes targeted by the cis-eQTLs and G_{trans} are the genes targeted by the trans-eQTLs. The proportion of trans-heritability is given by

$$\pi_{\text{trans}} = \frac{h^2_{\text{trans}}}{h^2_{\text{cis}} + h^2_{\text{trans}}} \tag{60}$$

The mean cis heritability across all simulations was found to be $h^2_{\text{cis}} = 3.8 \times 10^{-4}$. The distribution of π_{trans} over the 20 simulation replicates for each set of simulation parameters used in Fig. 2 (main text) is shown in Fig. S6b. In real data, π_{trans} is expected to be ≥ 0.7 from recent estimates [14] and the proportion of trans-heritability in our simulations is lower than these recent estimates. Since the trans effect sizes used in the simulations are close to our expectation in real data, the

number of TFs and / or target genes used in our simulation are possibly lower than what would be expected in real data. With fewer target genes it is harder to predict the trans-eQTLs using FR and RR of Tejaas, while simple regression methods like MatrixEQTL does not depend on the number of target genes.

Judging by the effect sizes and the heritability estimates, the parameters in our simulations represent a conservative estimate of our expectations in reality.

4.2 ROC pAUC analysis

We ranked the trans-eQTL predictions from each tool by descending score and computed the cumulative number of true and false positives (TP, FP) up to each score. We obtained the true

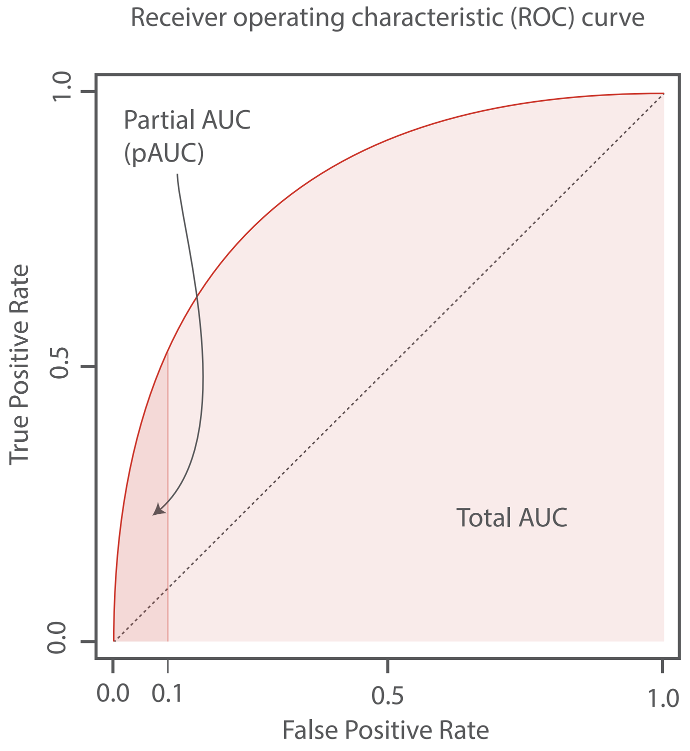

Fig. S7 | Definition of pAUC. The ROC curve shows the true positive rate (TPR) against the false positive rate (FPR) at various threshold settings. We measure predictive performance of all methods using the partial area under the ROC curve (pAUC) up to a FPR of 0.1.

positive rate (TPR) as the proportion of positives (with respect to total ground truth positives) correctly identified at that threshold; and the false positive rate (FPR) as the proportion of false positives (with respect to total ground truth negatives) identified at that threshold. This is often summarized as,

$$\mathrm{TPR} = \frac{\mathrm{TP}}{\mathrm{TP} + \mathrm{FN}} \tag{61}$$

$$\mathrm{FPR} = \frac{\mathrm{FP}}{\mathrm{FP} + \mathrm{TN}}, \tag{62}$$

where FN and TN denotes the false negative and the true negative respectively. The Receiver Operating Characteristic (ROC) curve shows the TPR against FPR at various thresholds (Fig. S7). Traditionally, ranking performance is measured by the full area under the ROC curve (AUC). However, AUC summarizes the entire ROC curve, including regions that are not relevant to

predicting trans-eQTLs (e.g., regions with low levels of specificity). In order to alleviate this deficiency while benefiting from some of the advantageous properties of the AUC, we used a partial area under the ROC curve (pAUC) which summarizes a portion of the curve over the pre-specified range of interest, FPR ≤ 0.1 (Fig. S7). Specifying the range of interest as FPR ≤ 0.1 is arbitrary.

To give a concrete example, we have 12639 SNPs of which 30 are trans-eQTLs. FPR = 0.1 corresponds to a threshold at which K top SNPs are selected such that there are 1261 false positives (10% of total 12609 true negatives) among those K selected SNPs. For the three methods compared here, suppose the number of predicted trans-eQTLs are K_{Tejaas}, K_{FR} and K_{MEQTL} for FPR=0.1. The higher the number of true positives at that specificity (= 1 - FPR = 0.9), the better is the method. However, the threshold p-value or FDR required for choosing K_{Tejaas}, K_{FR} and K_{MEQTL} are different.

4.3 Methods compared

We compared Tejaas RR-score with two methods: (1) MatrixEQTL [15] as a representative for current standard eQTL pipelines, and (2) Tejaas FR-score, as an alternative for CPMA [1] which uses the same underlying property of trans-eQTLs that they target multiple genes simultaneously.

MatrixEQTL. We used the R package for MatrixEQTL with default options. The covariate correction was done separately but used the same linear model correction implemented in MatrixEQTL. Since we were not interested in the cis-eQTLs, we used a cis-window of 0Kb.

FR (~CPMA). Since CPMA is not implemented as a software, we implemented the FR-score (q_{fwd}) within Tejaas as an alternative. Internally, the method runs over the data twice: (1) First, create an empirical null model from the data and (2) then calculate q_{rev} as well as an empirical p-value using the previously generated null model. This is the same procedure as CPMA, as described in their manuscript [1].

4.4 Supplementary simulation results

Selecting the regularizer. We plotted the non-Gaussian parameter $\alpha(\gamma)$ and the standard deviation of σ_q at different values of γ in Fig. S8a. As noted above in Sec. 2.8, the best choice of γ should be greater than $\arg\min_{\gamma} \alpha(\gamma)$. Ideally, we also want a high value for the standard deviation of σ_q to ensure a broad distribution of $q_{\text{rev}}^{\text{null}}$. Therefore, we chose $\gamma = 0.2$.

To validate our choice, we looked at the ranking of the trans-eQTLs at different values of γ in Fig. S8b. Each ROC curve was obtained by averaging over 20 simulations using default simulation parameters (see above) and KNN correction with 30 neighbors. In the inset, we show the partial areas under the ROC curves (pAUC) where the false positive rate (FPR) ≤ 0.1. We found that Tejaas works best at this choice of γ. The rest of the simulations were performed using $\gamma = 0.2$.

Note on inverse normal transform. Current GTEx pipeline uses a two-step approach for normalizing the read counts of the genes: (1) read counts are normalized using TMM (Trimmed Mean of M-values [16]) and filtered for thresholds, (2) expression values for each gene are then inverse normal transformed across samples. The gene expression generated in our simulations are not read counts, but equivalent to the TMM values. Hence we skipped the first step. We did perform the second step of inverse normal transformation before the confounder corrections.

As expected, we found that CCLM correction (Sec. 3.1) benefits significantly by using the inverse normal transformation (not shown). For the KNN correction (Sec. 3.2), however, we found that the inverse normal transformation reduces the accuracy of Tejaas (Fig. S9). It might be because the

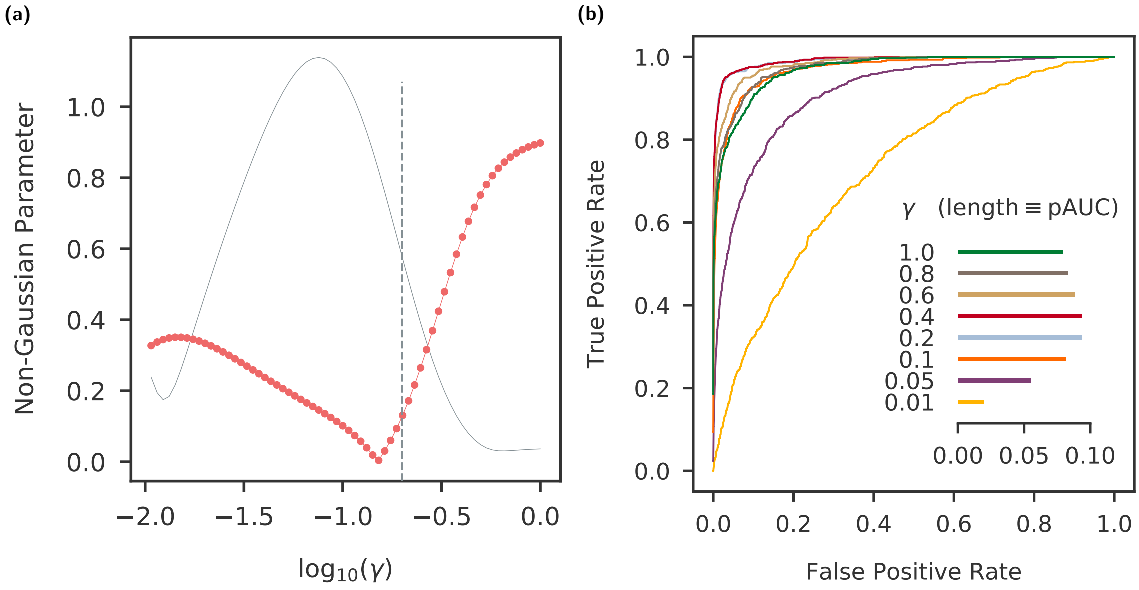

Fig. S8 | Selection of standard deviation of the regularizer in simulations. (a) To choose γ, we plotted the non-Gaussian parameter (red) at different values of γ. The solid gray line shows the standard deviation of σ_q. We chose γ = 0.2 shown by the dotted line. **(b)** We validated our choice by checking the ROC curve averaged over 20 simulations at different values of γ. In the inset we show the partial area under the ROC curve (pAUC) up to a false positive rate (FPR) of 0.1.

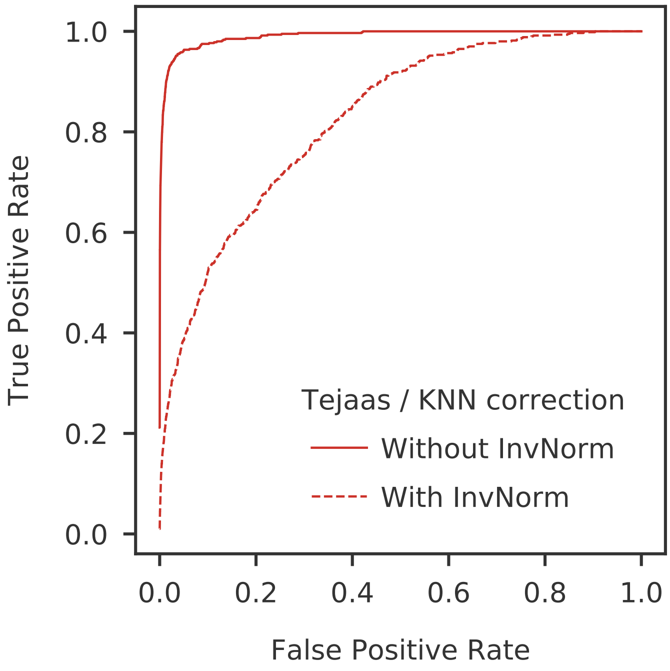

Fig. S9 | Inverse normal transform reduces power of Tejaas. We plot the ROC curves averaged over 20 simulations by applying KNN with 30 nearest neighbors with and without inverse normal transformation (InvNorm) of the gene expression data.

neighbor information gets skewed with this transformation. Hence, we performed KNN correction without the inverse normal transformation both for the simulations and the GTEx application.

Number of neighbors for KNN correction. Another important aspect of Tejaas is to choose the number of neighbors for the unsupervised KNN correction. To ascertain the robustness of the KNN correction, we performed simulations with different number of confounding factors, $C = 10, 20$ and 30. We then compared the ranking of Tejaas using different number of nearest neighbors, as shown in Fig. S10. The rankings were compared using the pAUC where the false positive rate (FPR) ≤ 0.1. We found that the choice of KNN neighbors does not depend on the number of confounding factors, and we obtained the best accuracy with 30 nearest neighbors.

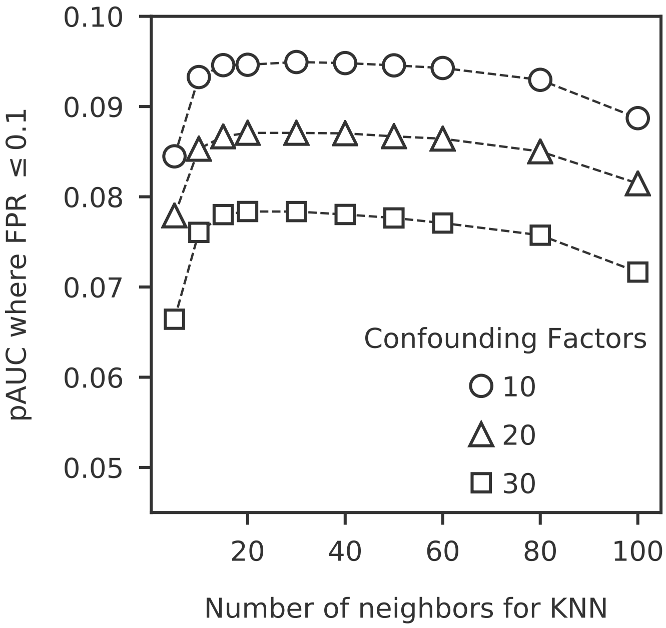

Fig. S10 | Optimization of number of neighbors used for KNN correction. We performed simulations with different number of confounding factors, $C = 10$, 20 and 30. The ranking of Tejaas using different number of nearest neighbors for KNN correction was measured using the pAUC where the false positive rate (FPR) ≤ 0.1, averaged over 20 simulation replicates.

Dependence on confounding factors. In Fig. S11, we compared different methods for discovering trans-eQTLs at different levels of confounding. The varying confounding effects were simulated by tuning: (1) the number of covariates, $C = 10, 20$ and 30, and (2) the effect size of the covariates on the gene expression obtained by varying the standard deviation ($\sigma_c = 0.4, 0.6, 0.8$ and 1.0) of the normal distribution (see Eq. (37)) from which the effect size is sampled. For discovering trans-eQTLs, Tejaas used KNN correction directly on the gene expression and q_{rev} with $\gamma = 0.2$. For MatrixEQTL and FR (~CPMA), all the known covariates were corrected using CCLM on the inverse normal transformed gene expression. We compared the accuracy of the methods using the partial area under the ROC curve (pAUC) where the false positive rate (FPR) ≤ 0.1.

Ranking of trans-eQTLs. Complementary to the partial area under ROC curves (Fig. 2 of main text), we also show the proportion of true positives (on average) included in the top ranked trans-eQTL SNPs in Fig. S12.

Target gene discovery. We investigated whether the increased accuracy of Tejaas to detect trans-eQTL SNPs also increases the accuracy of predicting trans target genes. As discussed in Sec. 2.9, Tejaas calculates single SNP-gene pairwise regression to find the set of candidate target genes. However, unlike MatrixEQTL, the trans-eQTL SNPs are preselected using reverse regression.

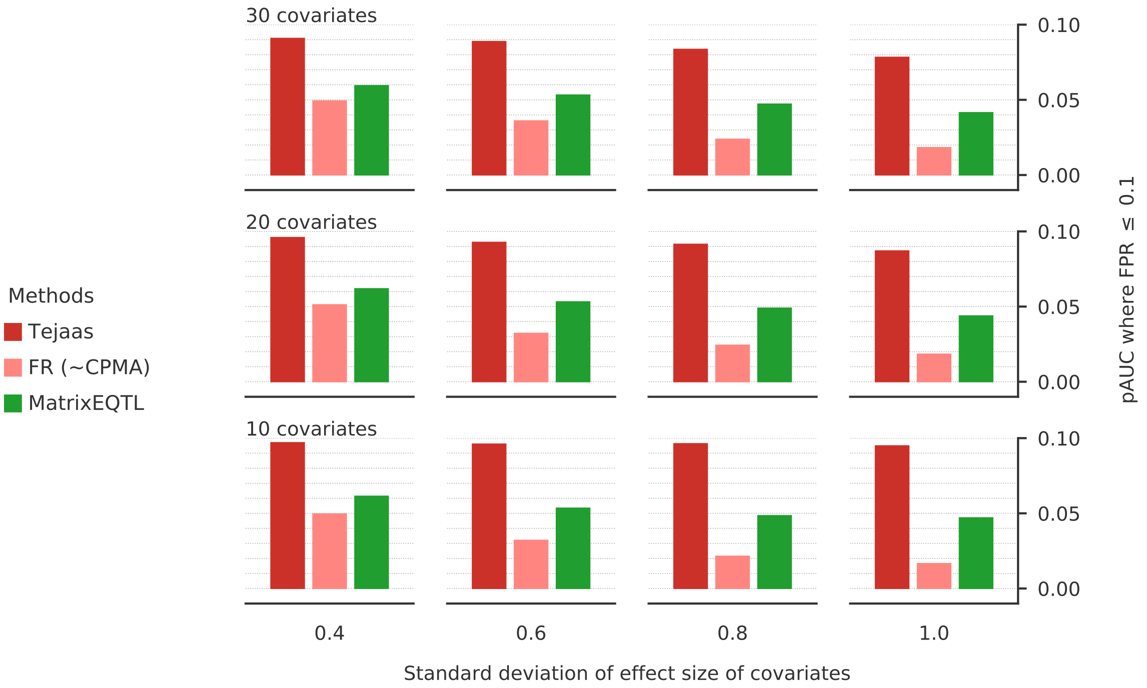

Fig. S11 | Effect of confounding factors in finding trans-eQTLs. We compare the ranking performance of Tejaas reverse regression, forward regression (FR) and MatrixEQTL in finding trans-eQTLs under different simulation settings with varying confounding effects. The variation in confounding effects were tuned by: (1) the number of covariates, $C = 10, 20$ and 30, and (2) the effect size of the covariates on the gene expression obtained by varying the standard deviation (σ_c = 0.4, 0.6, 0.8 and 1.0) of the normal distribution from which the effect size is sampled. For discovering trans-eQTLs, Tejaas used KNN correction directly on the gene expression and q_{rev} with $\gamma = 0.2$. For MatrixEQTL and FR (∼CPMA), all the known covariates were corrected using CCLM on the inverse normal transformed gene expression. Ranking performance of the methods are summarized using the partial area under the ROC curve (pAUC) where the false positive rate (FPR) ≤ 0.1.

The simulations provide us the ground truth to compare the performance of the different methods in predicting correct SNP-gene pairs.

The SNP-gene pairs reported by Tejaas, FR and MatrixEQTL were ranked by their p-values. Each true positive is a correctly predicted pair of trans-eQTL SNP and target gene, each false positive is a wrongly predicted pair. From the ROC curve, we calculated the pAUC up to a FPR of 0.1. The mean pAUC averaged over 20 simulations for each method and different simulation settings are shown in Fig. S13. For Tejaas and FR, we can limit the number of SNP-gene pairs to test by selecting trans-eQTL SNPs with $p_{\mathrm{RR\ or\ FR}} \leq p_{\mathrm{cutoff}}$. We tested three increasingly significant p-value cutoffs, namely 1×10^{-2}, 1×10^{-3} and 1×10^{-4}, and evaluated the sensitivity and specificity to discover the simulated true SNP-gene pairs. MatrixEQTL only tests single SNP-gene pair associations and therefore, we cannot perform the pre-filtering step.

Tejaas achieves higher accuracy for finding true SNP-gene pairs compared to FR and MatrixEQTL over a wide range of simulation settings. All methods use the same association test for predicting SNP-gene pairs and the only difference between the three methods is the preselection of trans-eQTL SNPs. Hence, the improvement of Tejaas over MatrixEQTL must be due to accurate preselection of trans-eQTL SNPs (see Fig. 2 of main text). MatrixEQTL needs to test a large number of comparisons ($I \times G$), and cannot benefit from the preselection step. FR performs poorly due to its poor sensitivity to predict trans-eQTLs in the preselection step (see Fig. 2 of main text). This analysis quantitatively shows that an increased accuracy to detect trans-eQTL SNPs can improve the prediction of trans-eQTL target genes, even when using simple regression methods.

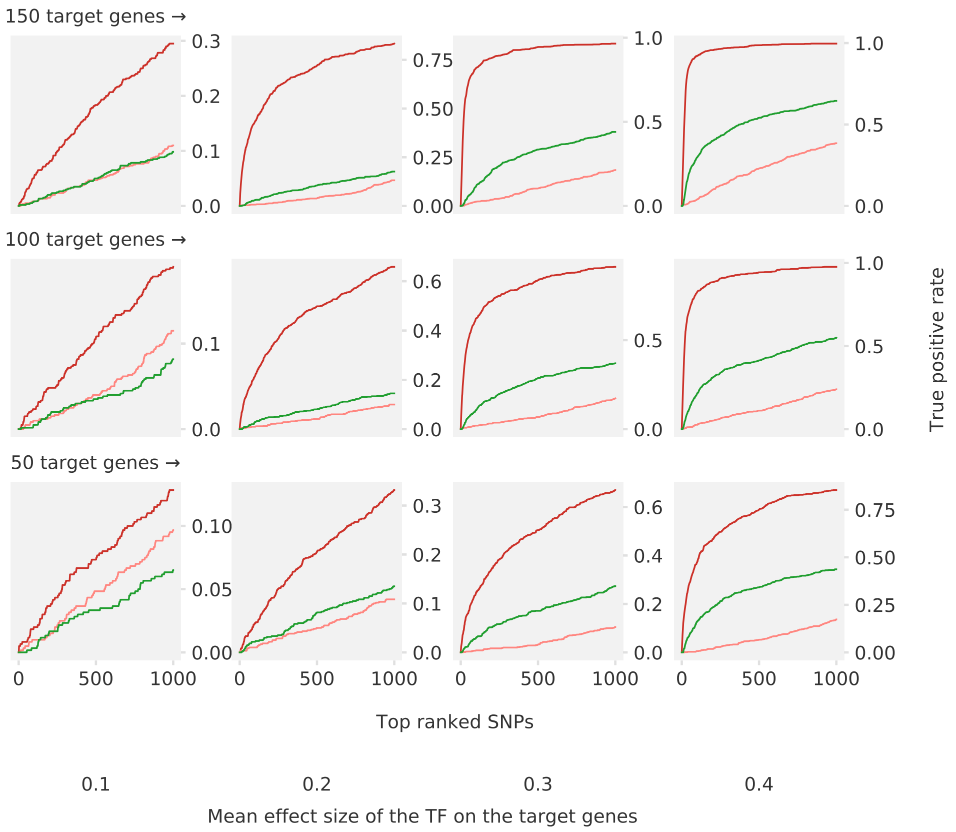

Fig. S12 | Sensitivity of different methods in finding trans-eQTLs. We compare the ranking performance of Tejaas reverse regression, forward regression (FR) (similar to CPMA) and MatrixEQTL, by the proportion of true positives (averaged over 20 simulations) included among the top ranked SNPs. Out of 12639 SNPs, we show the top 1000 predicted trans-eQTLs by the different methods. The y-axis shows the fraction of true positives out of the 30 planted trans-eQTLs in our simulations. Different panels correspond to different simulation settings (same as in Fig. 2 of main text): Each row has different numbers of target genes for the cis transcription factor (TF) mediating the trans-eQTLs, and each column has different mean effect sizes of the TF on the target genes. In each panel, the performance of the three methods are compared (colors as in Fig. 2 of main text, also in Fig. S11: Tejaas red, FR pink, MatrixEQTL green). Note that the y-axis scales differ between plots.

5 GTEx analysis

To illustrate Tejaas in a real data set, we analyzed trans-eQTLs across 49 human tissues using data from the Genotype Tissue Expression version 8 (GTEx v8) project [9–11]. In this section, we explain the preprocessing steps used for the GTEx analysis and report supporting results related to our analysis.

5.1 Genotype data

We downloaded the genotype files from the dbGaP portal (accession phs000424.v8.p2). The obtained genotype was quality filtered by the GTEx consortium [13]. The file downloaded from dbGaP (filename: `GTEx_Analysis_2017-06-05_v8_WholeGenomeSeq_838Indiv_Analysis_Freeze.SHAPEIT2_phased.vcf.gz`) contained 46 562 292 variants for 838 individuals. Genotype was split in chromosomes, variants with missing values were filtered out and sex chromosomes were removed. Finally, we retained only variants with minor allele frequency (MAF) ≥ 0.01 for further analysis (8 048 655 variants).

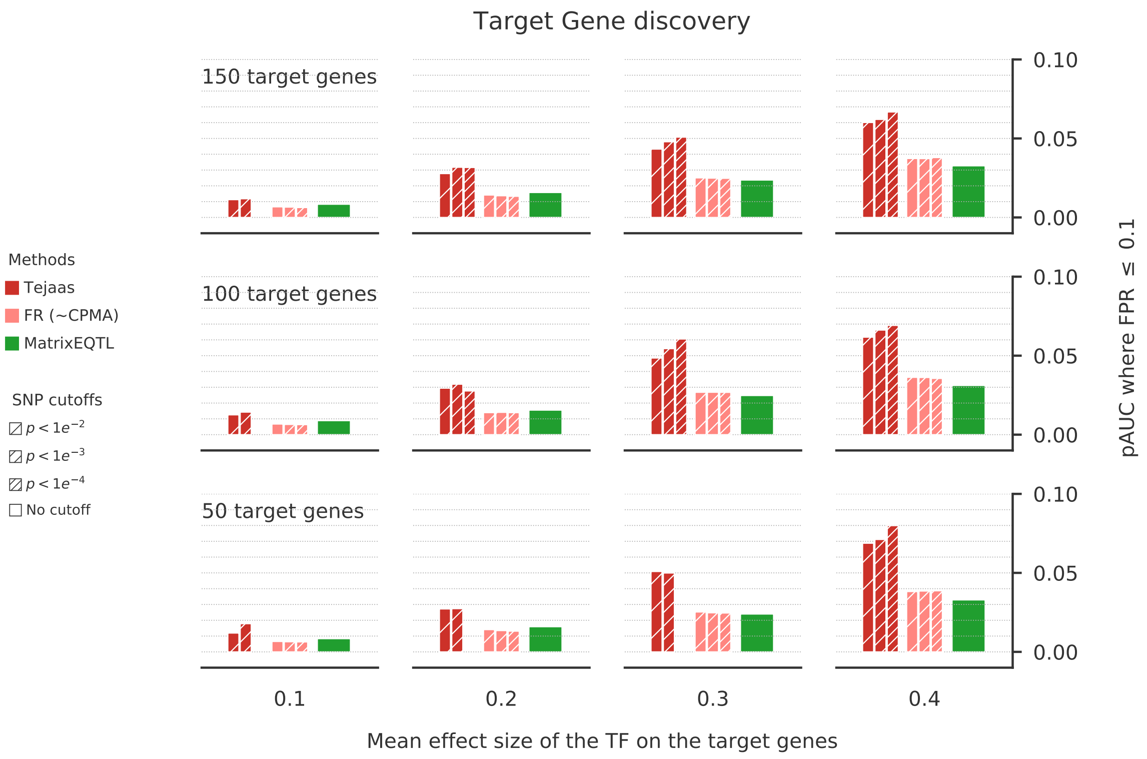

Fig. S13 | Comparison of ranking performance of different methods in finding target genes of trans-eQTLs. We evaluated the performance of Tejaas, FR and MatrixEQTL (color legends) for predicting correct SNP-gene pairs, ranked by their association p-values. Each bar is the mean partial area under the ROC curve (pAUC) up to a false positive rate (FPR) of 0.1, averaged over 20 simulations. Each panel represent a different simulation setting, with different numbers of target genes for the cis transcription factor (TF) mediating the trans-eQTL (from top to bottom) and with different mean effect sizes of the TF on the target genes (from left to right). For Tejaas and FR, trans-eQTLs were preselected using a p-value cutoff (pattern legend). MatrixEQTL cannot provide any preselection. (Also see caption of Fig. 2 in the main text.)

5.2 RNA-Seq expression data

We downloaded the phased RNA-seq read count expression matrix from the dbGaP portal (filename: `phASER_GTEx_v8_matrix.txt.gz`). The phASER processed expression uses read-backed mapping for each RNA-seq sample, using the genotype from the same individual as reference to map and phase RNA-seq reads. For each gene, two read count values are reported, one for each expressed allele. We added up both count values and calculated TPMs (Transcripts Per Million). For quality control, we retained genes with expression values > 0.1 and more than 6 mapped reads in at least 20% of the samples.

5.3 Covariate correction

Tejaas uses the TPMs (or TMMs) for KNN correction to remove confounders and subsequent trans-eQTL discovery. However, as discussed in Sec. 2.9, the explicit covariate-corrected gene expression is required for finding target genes of the trans-eQTLs. We downloaded the covariate files from the GTEx portal [17] (filename: `GTEx_Analysis_v8_eQTL_covariates.tar.gz`). The covariates include the first 5 principal components of the genotype (see Supplementary Material of [13] for details), donor sex, WGS sequencing platform (HiSeq 2000 or HiSeq X) and WGS library construction protocol (PCR-based or PCR-free). Additionally, from phenotype files available in dbGaP, we included donor age and post mortem interval in minutes ('TRISCHD') as covariates.

We inverse normal transformed the TPMs (or TMMs) and used CCLM (Sec. 3.1) to remove the contribution of all the covariates and used the residuals for target gene discovery.

5.4 Efficacy of KNN correction

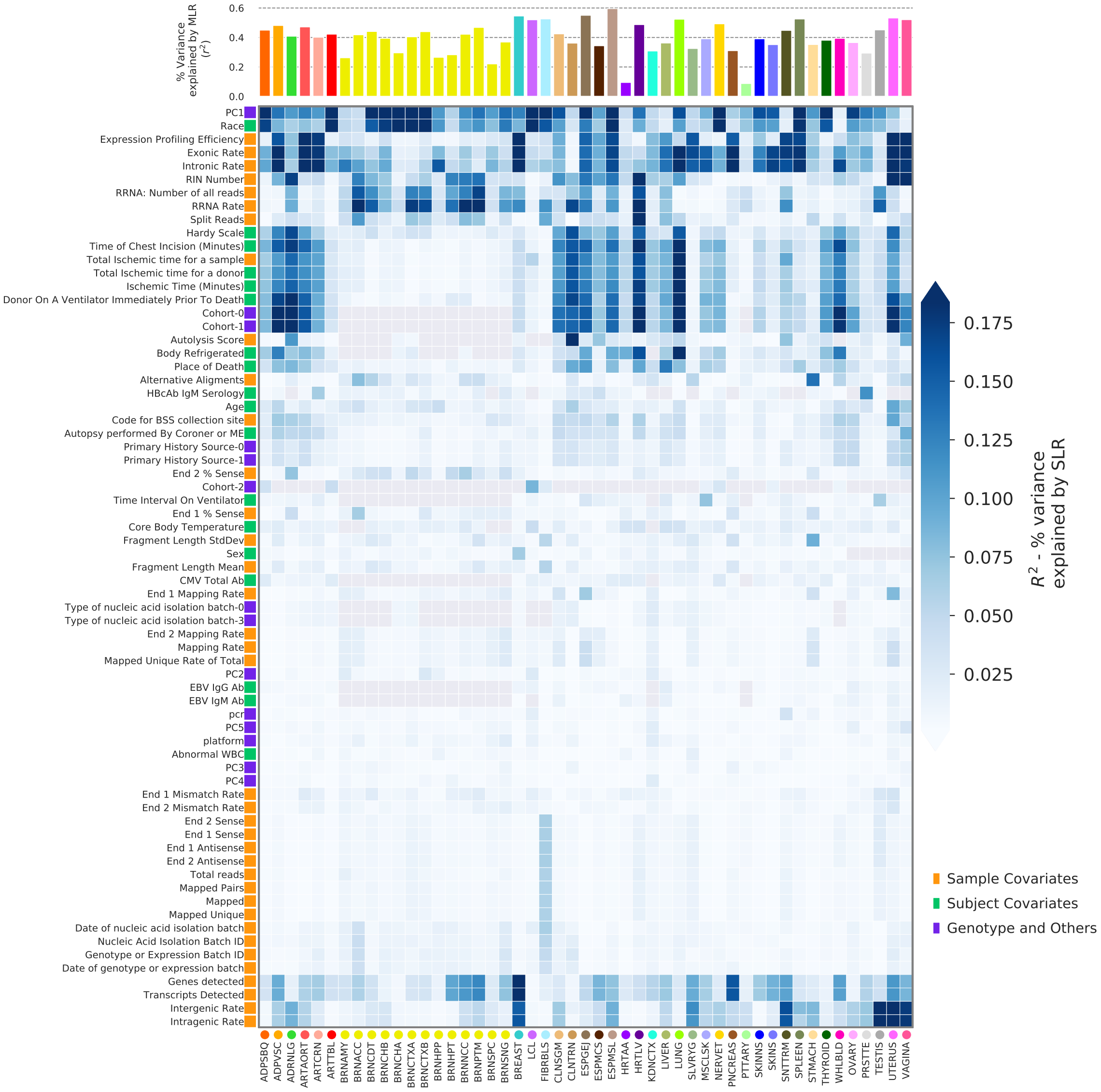

Fig. S14 | Known covariates of GTEx can explain a significant proportion of KNN correction. The proportion of variance of the KNN correction that can be explained by the known technical and biological covariates from GTEx can help us to understand the efficacy of KNN correction. In the bottom panel, each cell shows the coefficient of determination r^2 for a linear regression of the KNN confounder with the corresponding known covariate (y-axis) for each tissue (x-axis). On the top bar plot, we show the proportion of variance of the KNN confounder explained jointly by all known covariates with $r^2 > 0.05$.

One possible way to check the efficacy of the unsupervised KNN correction is to check whether it can be explained by the confounding effects of known covariates and if so, to what extent. To test this, we checked the correlation of the 'KNN confounder' with known technical and biological covariates of GTEx samples and subjects. For each tissue t, the KNN confounder ($\mathbf{C}_t^{\text{knn}} \in \mathbb{R}^{G \times N}$)

is simply the term we subtracted from the expression values of all the G genes in Eq. (32),

$$\mathbf{C}_t^{\text{knn}} = \frac{1}{K} \sum_{m \in \text{NN}_n^K} \mathbf{Y}_{mt} \,. \tag{63}$$

For every tissue t, we also obtained a list of C known covariates ($\mathbf{C}_{jt}^{\text{gtex}} \in \mathbb{R}^{G \times N}$ where $j \in \{1, \ldots, C\}$) by combining those obtained from the GTEx portal with a subset of sample and subject covariates from dbGaP (from filenames `phs000424.v8.pht002742.v8.GTEx_Subject_Phenotypes.data_dict.xml` and `phs000424.v8.pht002743.v8.GTEx_Sample_Attributes.data_dict.xml`). Categorical covariates with ≤ 4 categories were binarized or converted to integers otherwise. Values with 'Not Reported' or 'Unknown' status were considered as missing values and were not considered in the analysis. For each covariate, we selected only those with at least 50 observations in any given tissue.

We then performed a simple linear regression (SLR) to explain $\mathbf{C}_t^{\text{knn}}$ with $\mathbf{C}_{jt}^{\text{gtex}}$ as predictors using Python's `sklearn.linear_model.LinearRegression`,

$$\mathbf{C}_t^{\text{knn}} = \boldsymbol{\alpha}_{jt} \mathbf{C}_{jt}^{\text{gtex}} + \boldsymbol{\epsilon}_{jt} \,. \tag{64}$$

From the fitted models, we obtained the coefficient of determination r_{jt}^2 for every covariate j in tissue t. In other words, the r_{jt}^2 corresponds to the proportion of the variance of $\mathbf{C}_t^{\text{knn}}$ that can be explained by the jth known covariate in tissue t. The resulting r_{jt}^2 values are reported in Fig. S14 (bottom panel). Indeed, the variance of KNN correction are explained to different extents by several known covariates. For example, the first principal component of the genotype (PC1) and the sample's reported race both explain a significant proportion of the variance of $\mathbf{C}^{\text{knn}}$. The next four principal components, PC2 to PC5 do not contribute to explaining the variance of $\mathbf{C}^{\text{knn}}$. Interestingly, sample covariates related to library size and quality as well as various rates for mapped reads explain different proportions of the variance of $\mathbf{C}_t^{\text{knn}}$ in different tissues, indicating technical confounders in many GTEx tissues.

Next, we wanted to check the total proportion of the variance of $\mathbf{C}_t^{\text{knn}}$ explained by known covariates, which cannot be done using the SLR set up described above. For each tissue t, we selected those covariates with $r_{jt}^2 \geq 0.05$ in the SLR set up. We then performed a multiple linear regression (MLR),

$$\mathbf{C}_t^{\text{knn}} = \sum_{j=1}^{C_t'} \boldsymbol{\alpha}_{jt} \mathbf{C}_{jt}^{\text{gtex}} + \boldsymbol{\epsilon} \,, \tag{65}$$

where C_t' is the number of covariates retained in tissue t. The percentage of total explained variance of $\mathbf{C}_t^{\text{knn}}$ for each tissue is shown in the top panel of Fig. S14. On average across all tissues, the known covariates that we used explained 39.9% of the variance of KNN correction.

5.5 Tejaas regularizer selection for GTEx

As in simulations (Sec. 4.4), we selected the regularizer for the GTEx tissues using the non-Gaussian parameter (Sec. 2.8). From Fig. S15, we found that we could use $\gamma = 0.1$ for most of the tissues in GTEx v8. However, there were four tissues for which $\alpha(\gamma)$ at $\gamma = 0.1$ deviated strongly from the minimum. Hence for these four tissues, namely heart atrial appendage, pancreas, spleen and whole blood, we chose $\gamma = 0.006$.

(a)

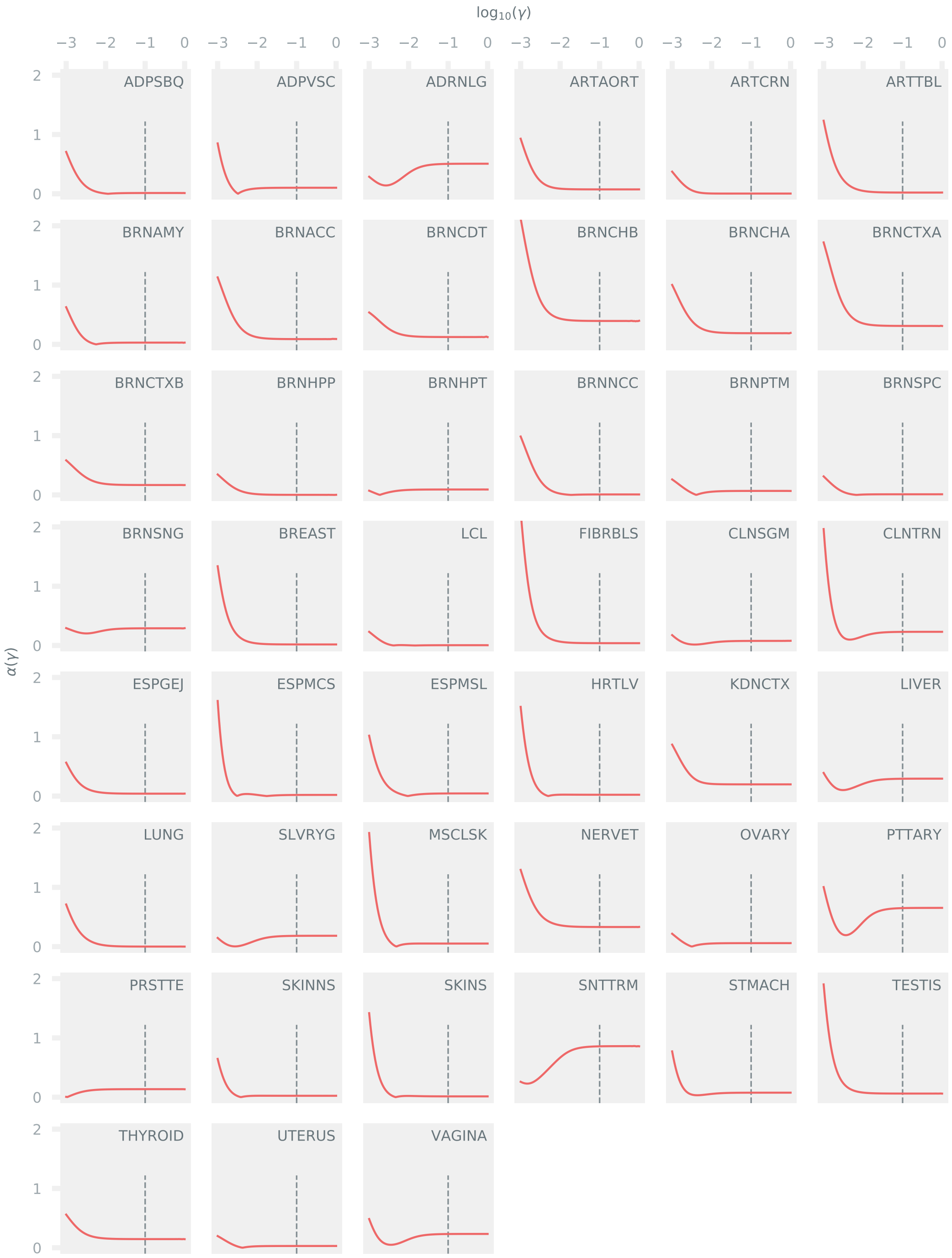

(b)

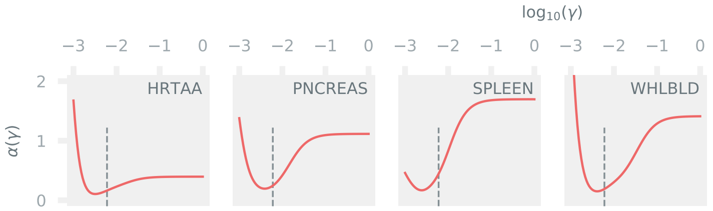

Fig. S15 | Non-Gaussian parameters for all GTEx tissues. We calculated the non-Gaussian parameters at different values of γ for all tissues in GTEx. We used the preprocessed gene expression from the GTEx v8 data and used simulated genotype to calculate the $\alpha(\gamma)$ for each tissue. The solid red line in each panel shows the variation of $\alpha(\gamma)$ for a given tissue with $-\log_{10}(\gamma)$. We divided the tissues into two groups: **(a)** for these tissues, we chose γ = 0.1, and **(b)** for these tissues, we chose γ = 0.006.

5.6 Tejaas results on GTEx

5.7 Method for calculating feature enrichments

We calculated whether the trans-eQTLs are enriched in several functional genomic features. For every such feature, we picked 5000 random SNPs from the GTEx genotype. and calculated the background fraction of SNPs expected by chance to have that feature,

$$f_{\text{bg}} = \frac{\text{number of SNPs with feature}}{\text{number of SNPs chosen randomly}} \tag{66}$$

This is repeated 50 times and the mean $\langle f_{\text{bg}} \rangle$ is considered as the background for that feature. Next, we calculated the fraction of trans-eQTLs with that feature,

$$f_{\text{trans-eqtl}} = \frac{\text{number of trans-eQTLs with feature}}{\text{number of trans-eQTLs in the tissue}} \tag{67}$$

Finally, we calculated the feature enrichment of trans-eQTLs in that tissue,

$$\rho_{ft} = \frac{f_{\text{trans-eqtl}}}{\langle f_{\text{bg}} \rangle} \tag{68}$$

We used a binomial test to calculate the p-values for the enrichment ρ_{ft}. If T is the number of trans-eQTLs in the tissue, then the probability of finding k of them with a feature is,

$$P(x = k) = \text{Binomial}\left(T, k, \langle f_{\text{bg}} \rangle\right) . \tag{69}$$

and $P(x > k)$ gives us the p-value for the tissue-GWAS pair.

5.8 Regulatory element enrichment

To better understand the possible molecular mechanisms that drive trans-eQTL effects, we calculated enrichments of the trans-eQTLs at functional annotations in the genome. Annotations were obtained from GTEx Portal (`WGS_Feature_overlap_collapsed_VEP_short_4torus.MAF01.txt.gz`). For our analysis, we considered three sets of trans-eQTLs: (i) the set of all trans-eQTLs, (ii) trans-eQTLs that are only found in one tissue and (iii) trans-eQTLs that are found in two or more tissues (Fig. S16 'All', 'N1' and 'N2+', respectively). We found that trans-eQTLs are significantly enriched in enhancers, open chromatin regions, promoter flanking regions and transcription factor binding sites ($p < 2.42 \times 10^{-05}$, $p < 4.4 \times 10^{-05}$, $p < 2.42 \times 10^{-16}$ and $p < 9.9 \times 10^{-04}$ respectively). On the other hand, trans-eQTLs are only slightly enriched in promoter regions ($\rho = 1.10$, $p = 0.08$) and depleted on CTCF binding sites ($\rho = 0.86$).

5.9 Effect of cis-masking

We applied Tejaas on 38 GTEx tissues (except brain tissues and bladder) with and without cis-masking (Sec. 2.10) to check the effect of cis-masking option of Tejaas for discovering trans-eQTLs in the GTEx data. We found that $\sim 97\%$ of the trans-eQTLs discovered with cis-masking (shown with green in Fig. S17a) are also discovered without cis-masking (shown with red in Fig. S17a). However, if no cis-masking is used, Tejaas discovers $\sim 11\%$ more trans-eQTLs compared to that with cis-masking. This increase in significant trans-eQTLs might be due to strong cis-eQTLs, as envisioned in Sec. 2.10.

We can understand the effect of cis-masking by looking at how many of our trans-eQTLs are also reported as cis-eQTLs by the GTEx Consortium [13]. We defined the proportion of cis-eQTLs

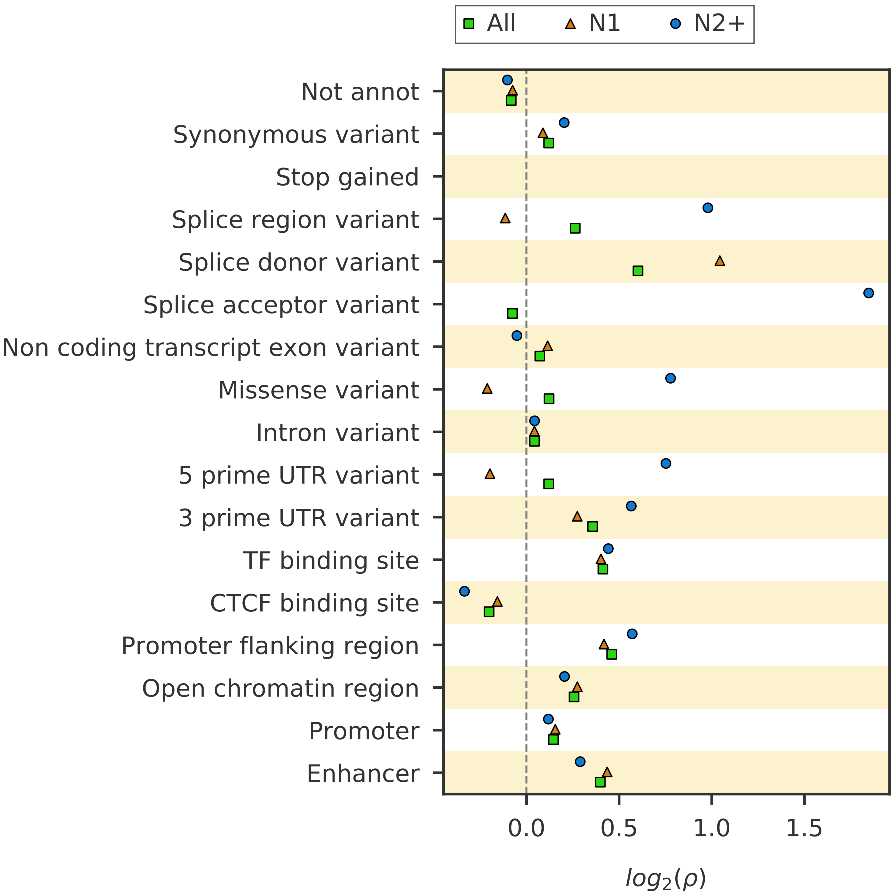

Fig. S16 | Trans-eQTLs are enriched in known regulatory elements. Enrichment in genomic functional annotations can give us insights into how trans-eQTLs affect gene expression. We considered three sets of trans-eQTLs: (i) the set of all trans-eQTLs ('All'), (ii) trans-eQTLs that are found in only one tissue ('N1') and (iii) trans-eQTLs that are found in two or more tissues ('N2+'). Genomic functional annotations were obtained from the GTEx portal [17].

among the trans-eQTLs compared to the proportion expected by chance as cis-eQTL enrichment ($\rho_{\text{cis-eQTL}}$). In Fig. S17b, we compare the $\log_2(\rho_{\text{cis-eQTL}})$ for trans-eQTLs discovered with and without cis-masking in all the 38 tissues. The mean $\log_2$ enrichment of cis-eQTLs across all tissues (shown in inset of Fig. S17b) is 0.10 with cis-masking and increases to 0.77 without cis-masking. This increase comes from the extra $\sim 11\%$ SNPs discovered without cis-masking, indicating that cis-masking is crucial to avoid strong cis-eQTLs being falsely reported as trans-eQTLs by Tejaas.

5.10 Effect of cross-mappable genes

False trans-eQTL signals could arise from multi-mapped reads within genes with high sequence similarity, where reads from a given gene are mapped to one or more other genes elsewhere in the genome and showing an association resembling a long range regulatory effect. This problem was explored by Saha and Battle [18] and can be mitigated using cross-mappability scores. We obtained pre-computed cross-mappability scores from [18], with settings of k-mer length 75 for exons and 36 for UTRs. In Tejaas we extended the cis-masking approach to exclude all genes that cross-map (cross-mappability score > 1) with any of the cis-genes listed in a given cis-masking group and we call this the cross-mappability filter.

We applied our trans-eQTL discovery pipeline on all non-brain tissues of GTEx with the cross-

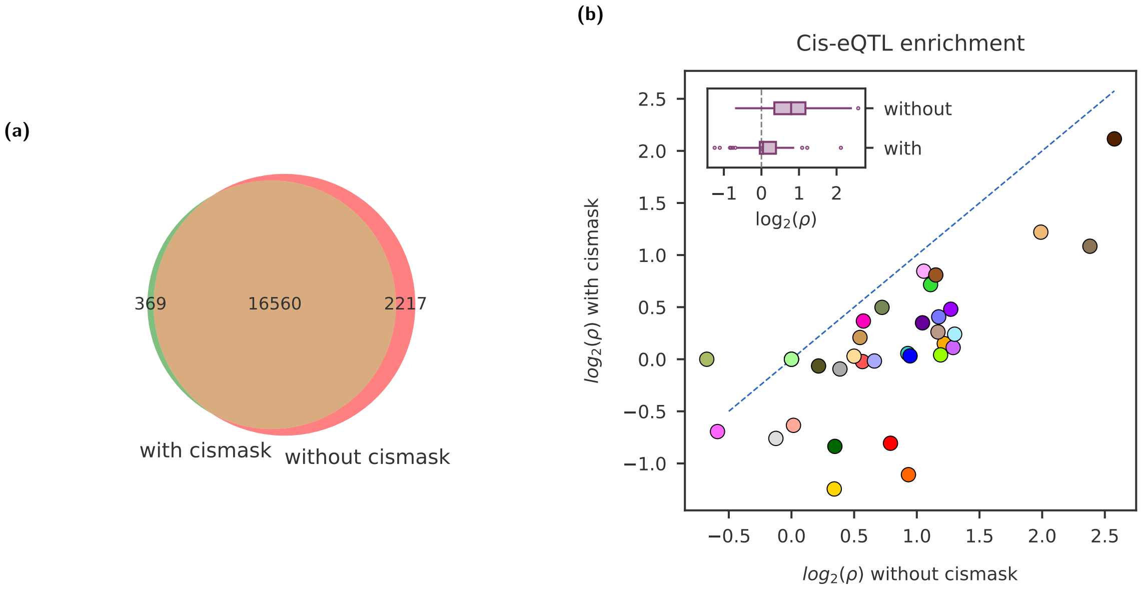

Fig. S17 | Cis-masking controls falsely identifying cis-eQTLs as trans-eQTLs. Cis-eQTLs with strong association with cis-eGenes may lead them to be discovered as trans-eQTLs by Tejaas. Exclusion of cis-genes greatly reduces the discovered cis-eQTLs while having minimal impact on the discovery of trans-eQTLs. **(a)** Overlap of trans-eQTLs with and without cis-masking. 97% of trans-eQTLs, which are found with cis-masking are also discovered without cismasking. **(b)** Comparison of cis-eQTL enrichment with and without cis-masking. Cis-eQTL enrichment reduces significantly in all tissues when cis-masking is enabled, indicating that the extra trans-eQTLs discovered without cis-masking are mostly cis-eQTLs.

Tissues	γ	Before crossmap	After crossmap	
		Reported	Filtered out	Brought in
Set 1	0.1	16920 (11161)	5856 (4996)	1360 (1276)
Set 2	0.006	7866 (7518)	607 (603)	6481 (6419)
Set 2	0.008	–	3081 (3031)	732 (731)

Table S1 | Summary of number of trans-eQTLs before and after cross-mappability filter. We report the two sets of tissues, Set 1 contains 32 non-brain tissues for which we used $\gamma = 0.1$ in our analysis, and Set 2 contains 4 tissues for which we used $\gamma = 0.006$. We report the total number of lead trans-eQTLs after LD pruning for the different analyses. Numbers in bracket are the number of unique trans-eQTLs in that given set.

mappability filter. The cross-mappability filter masks out a large number of genes, often more than thousands. For example, the average number of cis-genes masked in whole blood tissue is ~ 9, while the average number of cross-mapped genes masked for whole blood tissue becomes ~ 3508.

About 34% of trans-eQTLs were filtered out for tissues with $\gamma = 0.1$ (Table S1 and Fig. S18a), indicating that they might be false signals from cross-mapped genes. However, we unexpectedly observed an increase in the number of predicted trans-eQTLs in the set of four tissues (whole blood, heart atrial appendage, spleen and pancreas) where we used $\gamma = 0.006$. This is because removing the large number of cross-mapped genes breaks down the normality assumption of the null model for these tissues, which are very sensitive to the choice of γ, as can be seen from their non-Gaussian parameter (Fig. S15). For the other tissues, the choice of γ is relatively stable and removing the cross-mapped genes does not affect the choice. For our assumptions to hold, we need to optimize γ for every SNP in these tissues, to consider every set of masked genes when using the cross-mappability filter. This optimization is time consuming. However, with an

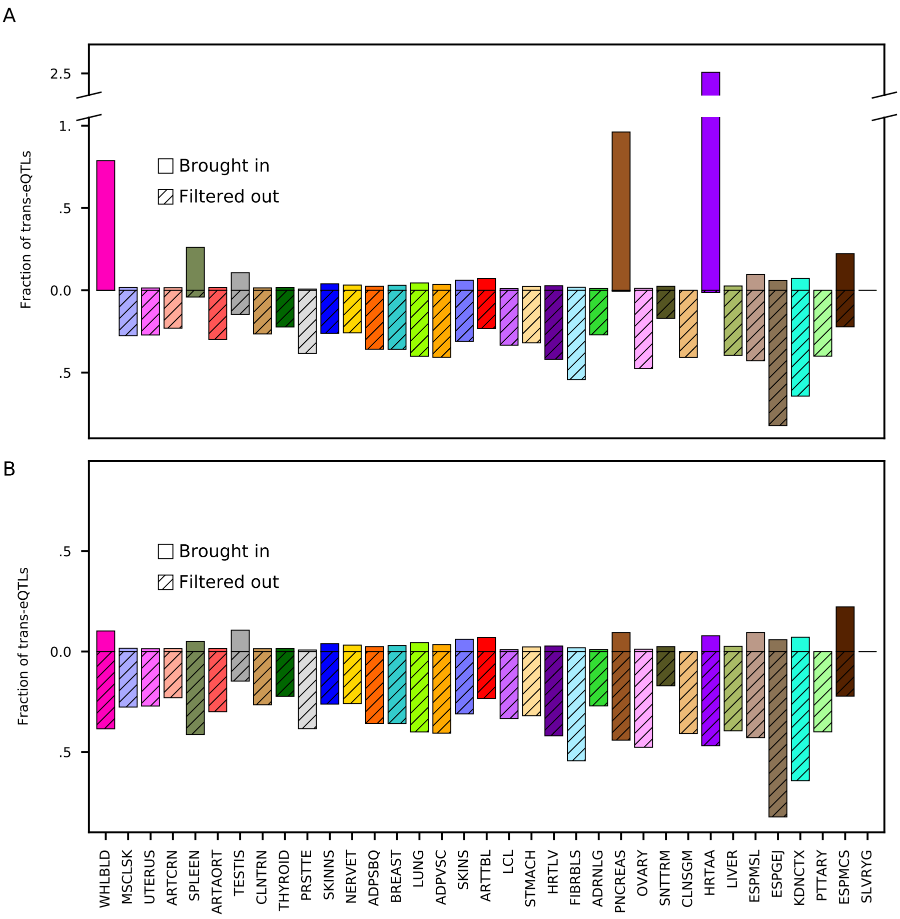

Fig. S18 | Effect of cross-mappable genes on trans-eQTL discovery. For each tissue, we show the fraction of significant and non-significant SNPs (brought in and filtered out, respectively) after cross-mappability filter with respect to the number of significant SNPs before the filter was applied. **A** Results using optimization of γ before cross-mappability filter (no optimization after cross-mappability filtering). **B** Results using optimization of γ before and after cross-mappability filter.

approximate choice of $\gamma = 0.008$ obtained by optimizing $\alpha(\gamma)$ after removing a random set of 3000 genes from the expression data, the results are comparable to those found in other tissues (Table S1 and Fig. S18b).

We compared the enrichment in functional regions and cis-eQTLs before and after cross-mappability filter. Enrichment of trans-eQTLs in DHS regions and cis-eQTLs does not change significantly ($p > 0.1$) with and without the cross-mappability filter (Fig. S19). Despite this, we do observe a slight decrease in the enrichment of cis-eQTLs for many tissues, consistent with the false positive trans-signal that the cross-mappability filter is meant to remove.

5.11 Effect of GTEx populations in predicted trans-eQTLs

To assess the effect of population differentiation in GTEx, we calculated fixation index values (F_{ST} values) as in [19] on GTEx genotype data. We compared the distribution of F_{ST} values for all GTEx SNPs with the set of trans-eQTLs predicted with Tejaas (Fig. S20, left panel). The average F_{ST} value for the predicted trans-eQTLs in each tissue is shown on the right panel of Fig. S20. High F_{ST} values are indicative of allele frequency differences between subpopulations present in the data. Population substructure could give spurious indications of eQTL associations [20]. To rule

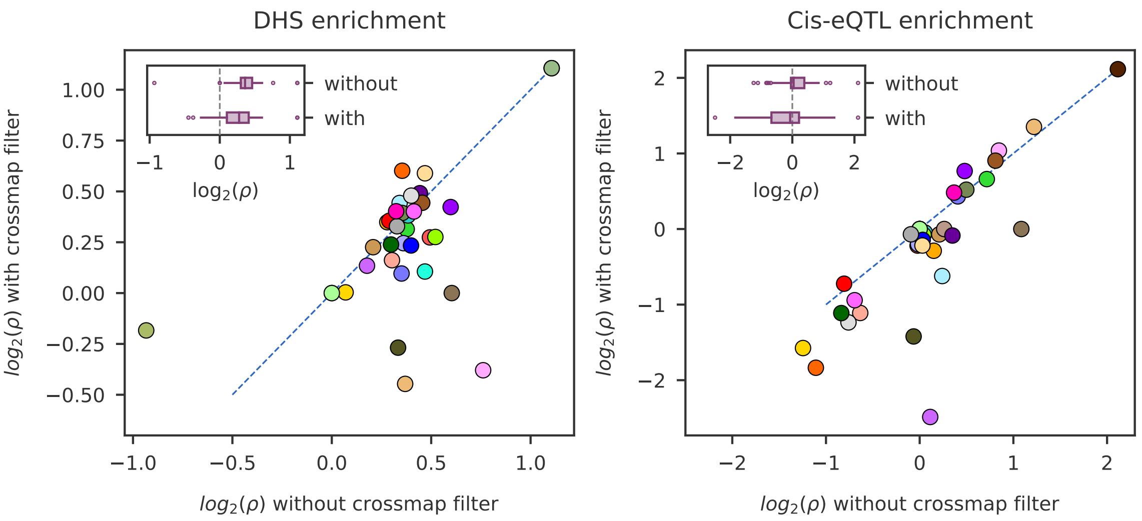

Fig. S19 | Effect of cross-mappable genes on functional enrichment of trans-eQTLs. Comparison of DHS enrichment and cis-eQTL enrichment with and without cross-mappable genes show that there is no significant difference. A number of GTEx tissues show lower enrichments when the cross-mappable filter is applied.

out confounding effects of population differentiation from our enrichment analysis, we sampled null sets with the same F_{ST} distribution as that of the predicted trans-eQTLs. We re-calculated DHS functional enrichments (Fig. S21) and GWAS enrichemnts (data not shown). However, we did not observe any significant deviation from the previously calculated enrichments.

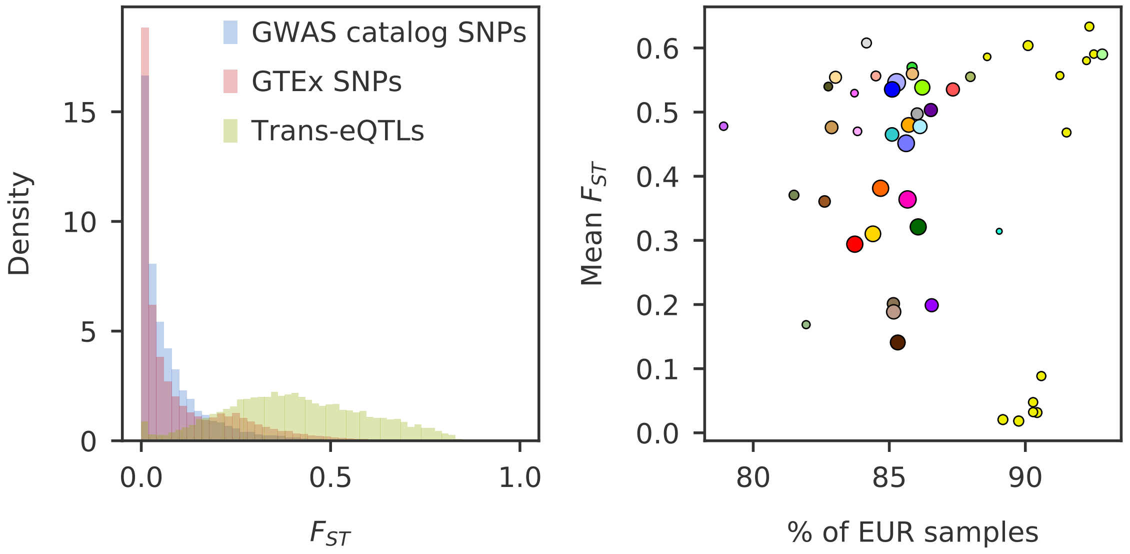

Fig. S20 | Distribution of F_{ST} values in GTEx and predicted trans-eQTLs. Left panel: Distribution of F_{ST} values for all the GTEx SNPs analyzed (red), all SNPs present in GTEx and in the GWAS catalog (blue) and the trans-eQTLs predicted with Tejaas (green). Area under the curve is normalized to 1. Right panel: Mean F_{ST} values of predicted trans-eQTLs in each tissue, colored in GTEx colors. Size of the dot indicates number of samples.

5.12 Regulatory element enrichment in brain tissues

Brain tissues in GTEx have generally lower number of RNA-seq samples compared to other tissues. This is one of the reasons why they display lower number of predicted trans-eQTLs with Tejaas.

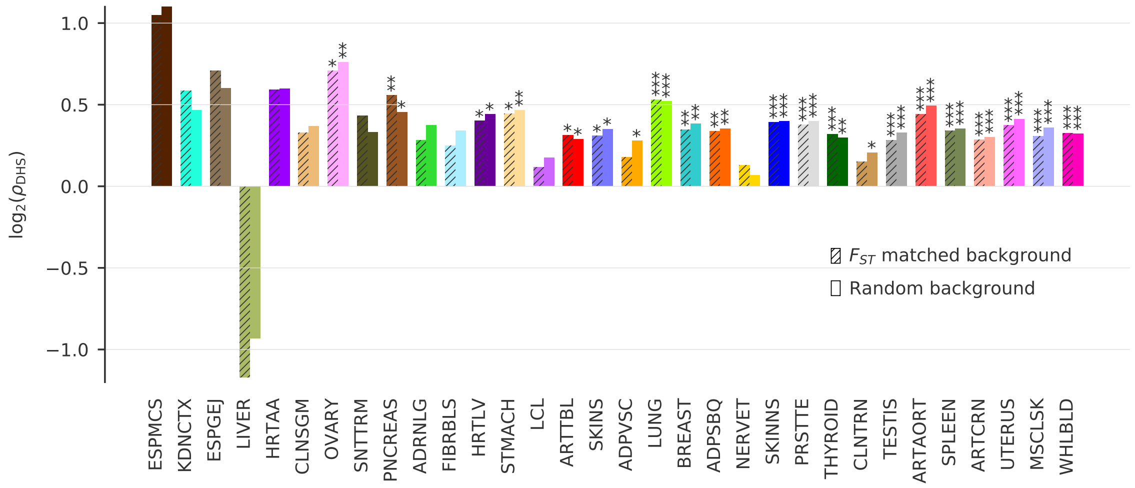

Fig. S21 | DHS enrichment of trans-eQTLs after adjusting background for F_{ST}. We account for the enrichment of predicted trans-eQTLs in F_{ST} values by adjusting the background enrichment to have the same F_{ST} distribution as the one for predicted trans-eQTLs in each tissue.

Other unknown confounders in the expression measurements could also affect Tejaas predictions, as it has been reported that brain tissues have markedly distinct expression signatures in GTEx that separates them from the rest of the tissues. This could be due to the post mortem nature of the samples. We report DHS, raQTL and functional region enrichments for trans-eQTLs predicted in brain tissues in Fig. S22. Due to the low number of predictions, the reported enrichments are not reliable.

5.13 Performance of Tejaas on null data

On null data, Tejaas does not show any spurious association (Fig. S23). We used the same gene expression as used for trans-eQTL discovery to check if the correlation of the gene expression can lead to false discovery. We removed any possible trans-eQTL signal by permuting the sample labels of the genotype. We found no significant association in any GTEx tissue.

5.14 Replication in eQTLGen

We downloaded the list of significant trans-eQTLs discovered in the eQTLGen study from `https://www.eqtlgen.org/trans-eqtls.html` (version from 2018-09-04). It contains 59 786 SNP-gene pairs and a total of 3 853 unique trans-eQTLs in whole blood. In Appendix 3, we report the overlap between trans-eQTL from eQTLGen with the lead trans-eQTLs discovered with Tejaas in all GTEx tissues. Enrichments were calculated as in Sec. 5.7.

Replication across all GTEx tissues is low, with whole blood showing the highest number of replicated variants. Although we don't expect to replicate other studies that used single SNP-gene pairs association methods, it is reassuring to see that Tejaas can replicate trans-eQTLs discovered in the same tissue type. Nonetheless, we must highlight that the eQTLGen meta-analysis study includes GTEx whole blood expression.

6 GWAS analysis

We investigated the overlap of the novel trans-eQTLs discovered by Tejaas with GWAS variants of complex traits to find transcriptional regulatory mechanisms through which SNPs affect complex

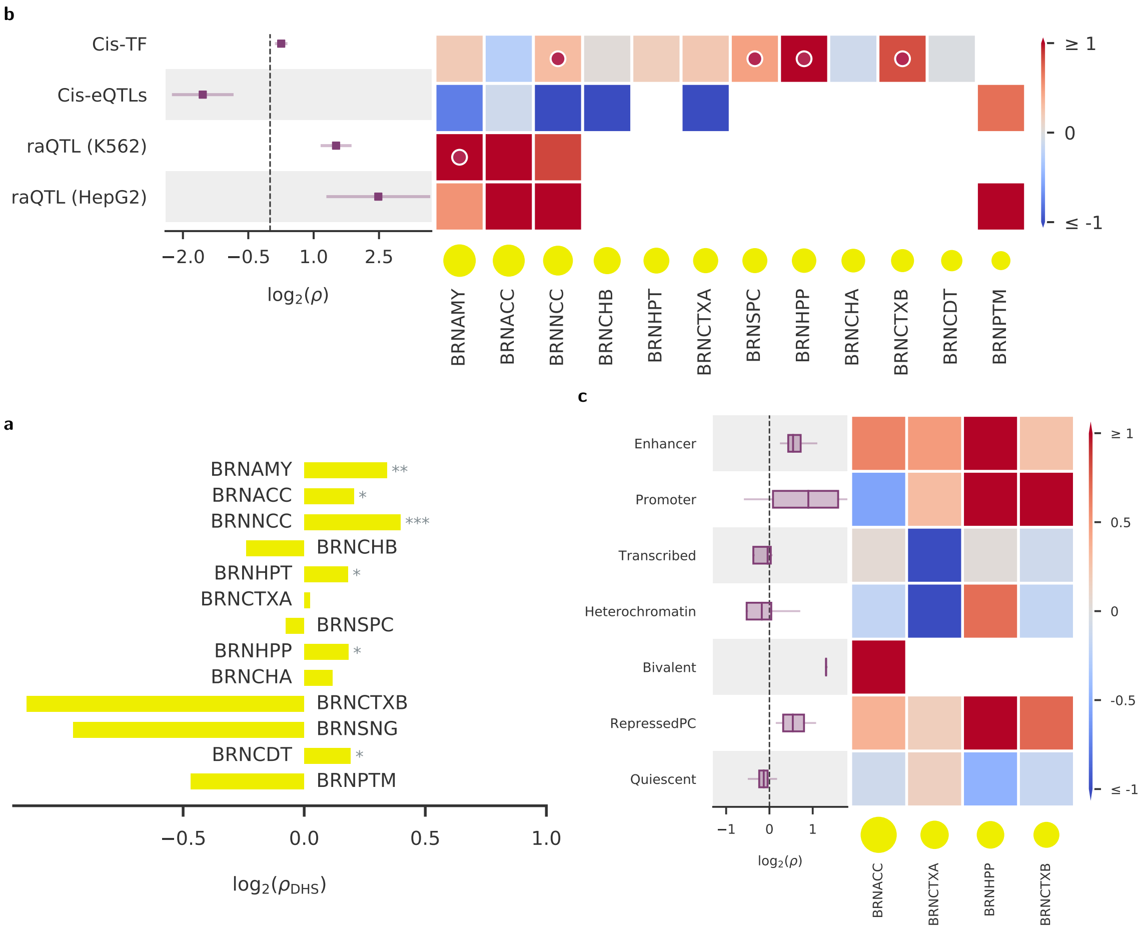

Fig. S22 | Functional enrichments in brain tissues. a, Log_2 enrichments (x-axis) within accessible chromatin regions from [21]. **b**, Log_2 enrichments near known eQTLs, Transcription Factors and reporter assay QTLs (raQTLs). **c**, Log_2 enrichments within tissue-specific regulatory regions. For more details, see caption of Fig. 04 in main text.

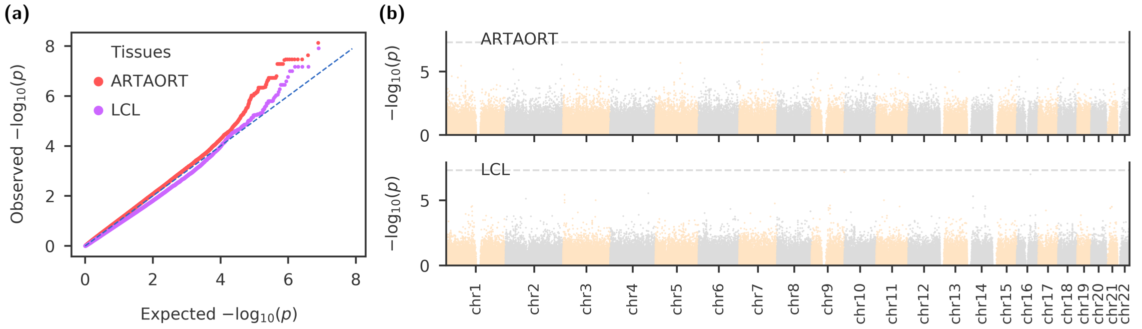

Fig. S23 | Performance of Tejaas on null data. We removed possible trans-eQTL signal from the GTEx data by permuting the sample labels of the genotype. We did not permute the gene expression of the tissues to retain the inherent correlation among the genes. **(a)** Representative example of quantile-quantile plot from null data in two tissues: artery aorta (ARTAORT) and EBV-transformed lymphocytes (LCL). **(b)** Corresponding Manhattan plot for the above two tissues, showing the $-\log_{10}(p)$ values for genome-wide variants.

diseases. In this section, we discuss the details of our analyses and report supplementary results.

6.1 Data source for GWAS summary statistics

We used two different libraries of GWAS-associated SNPs:

1. **GWAS Catalog.** Data was obtained from the EBI website [22]. We used version 'e98_r2020-03-08' which contains 4493 studies with a total of 179 364 associations [23, 24].

2. **Complex trait GWAS.** A set of 87 GWAS compiled by by Barbeira *et al.* [25]. Summary statistics from these GWAS were harmonized and imputed to GTEx v8 variants with MAF > 0.01 using only European samples. Of these, 86 traits had at least one genome-wide significant SNP ($p < 5 \times 10^{-8}$) among the GTEx v8 variants, which were tested for trans-eQTLs. These 86 traits were broadly classified into 11 disease categories.

6.2 Calculation of GWAS enrichment

We used two different strategies for calculating the enrichment of trans-eQTL SNPs in GWAS for the two different datasets.

GWAS catalog. We used 5000 randomly chosen SNPs from the GTEx genotype to calculate the fraction of SNPs that overlap with the GWAS catalog SNPs expected by chance,

$$f_{\text{bg}} = \frac{\text{number of random SNPs overlapping with GWAS SNPs}}{5000} \tag{70}$$

This is repeated 300 times and the mean $\langle f_{\text{bg}} \rangle$ is considered as the background for that GWAS. We then calculated the fraction of lead trans-eQTLs observed in the GWAS,

$$f_{\text{trans-eqtl}} = \frac{\text{number of lead trans-eQTLs overlapping with GWAS SNPs}}{\text{number of lead trans-eQTLs in the tissue}} \tag{71}$$

and the GWAS enrichment of trans-eQTLs in that tissue,

$$\rho_{\text{GWAS}} = \frac{f_{\text{trans-eqtl}}}{\langle f_{\text{bg}} \rangle} \tag{72}$$

Complex trait GWAS. In this case, we wanted to compare the GWAS enrichment of trans-eQTLs with that of GTEx cis-eQTLs with varying cutoff of GWAS p-values. Hence, we plotted the fraction of cis-eQTLs, the fraction of trans-eQTLs and the fraction of total tested SNPs that overlap with SNPs associated with a complex disease (or disease category), as shown in the representative example of Fig. S24a. We used different cutoff p-values for selecting the GWAS associated SNPs. Each disease category is a collection of several disease phenotypes. To obtain category-wise p-values for each SNP, we assigned the minimum GWAS p-value from the phenotypes that constituted the category. Finally, we calculated the GWAS enrichment of eQTLs (cis and trans respectively),

$$\rho_{\text{GWAS}} = \frac{\text{fraction of eQTLs overlapping with GWAS SNPs}}{\text{fraction of tested SNPs overlapping with GWAS SNPs}} \tag{73}$$

This is shown in the representative example of Fig. S24b. We used all reported cis-eQTLs and all predicted trans-eQTLs without pruning for LD regions to calculate the overlaps and enrichments.

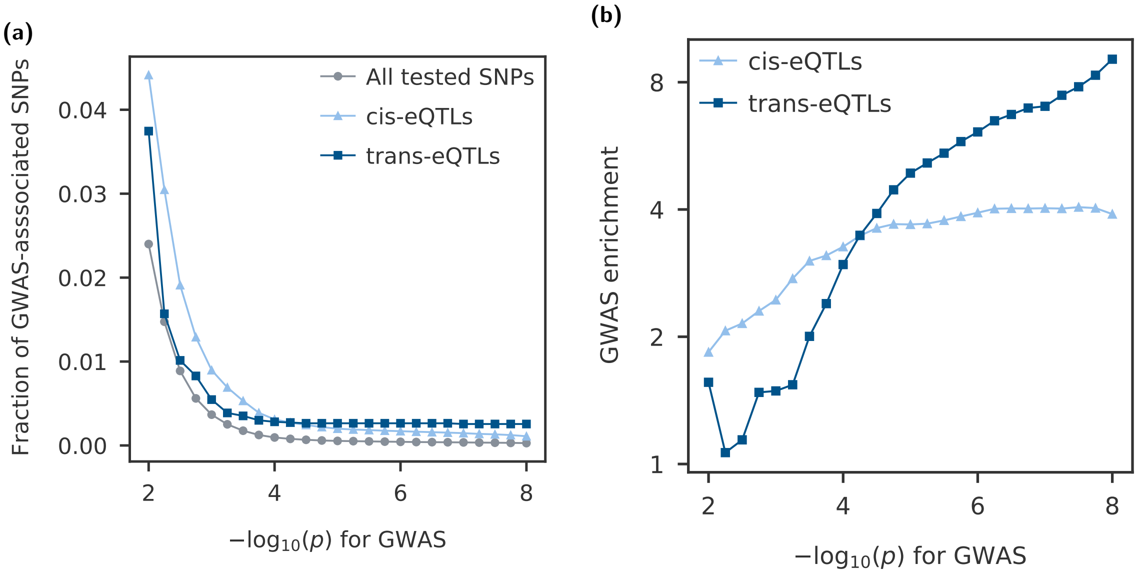

Fig. S24 | Calculation of GWAS enrichment. Representative example of GWAS enrichment for muscle skeletal tissue and disease category of skeletal muscle diseases at different GWAS *p*-value cutoffs (x-axis). **(a)** The proportion of cis-eQTLs (light blue, triangles) and trans-eQTLs (dark blue, squares) that overlap with GWAS-associated SNPs. The proportion of all tested variants which are associated with the disease category is shown in gray circles. **(b)** GWAS enrichment of cis-eQTLs and trans-eQTLs are calculated from the fractions observed in (a) using Eq. (73).

P-value calculation. We used a binomial test to calculate the p-values for the enrichment of each tissue-GWAS pair. If T is the number of trans-eQTLs in the tissue, then the probability of finding k of them in a GWAS is given by,

$$P(x = k) = \text{Binomial}\left(T, k, \langle f_{\text{bg}} \rangle\right) . \tag{74}$$

and $P(x > k)$ gives us the p-value for the tissue-GWAS pair.

Table S2 | GWAS enrichment of trans-eQTLs for different tissues and disease categories. (See Fig. 5b of main text.) All pairs of tissues and disease categories which showed significant enrichment ($p < 0.05$) for trans-eQTLs to overlap with GWAS-associated SNPs, selected at a nominal cutoff of $p < 1 \times 10^{-6}$

Disease category	Tissue	Enrichment	Enrichment p-value
Aging	LCL	41.57	0.0011202
Allergy	SKINS	11.15	3.75855e-30
Allergy	ADPVSC	9.51	1.24242e-25
Allergy	CLNSGM	7.32	0.00020217
Allergy	OVARY	7.24	0.00160797
Allergy	LUNG	7.04	1.49904e-18
Allergy	PNCREAS	6.32	6.18969e-06
Allergy	SKINNS	6.12	1.78606e-21
Allergy	FIBRBLS	4.71	0.000164823
Allergy	UTERUS	3.37	2.03157e-18
Allergy	PRSTTE	3.28	2.80764e-09
Allergy	ARTAORT	3.20	1.45783e-11

Disease category	Tissue	Enrichment	Enrichment p-value
Allergy	CLNTRN	2.73	1.575e-07
Allergy	BREAST	2.50	0.0056074
Allergy	MSCLSK	2.28	4.15921e-09
Allergy	SPLEEN	2.17	8.54041e-05
Allergy	ARTCRN	2.16	5.76719e-06
Allergy	BRNNCC	2.06	0.0446224
Anthropometric	ADRNLG	8.68	4.23834e-140
Anthropometric	BRNCTXB	3.69	0.00277057
Anthropometric	LIVER	3.53	3.678e-07
Anthropometric	CLNTRN	2.94	7.06009e-94
Anthropometric	ADPVSC	2.50	8.97789e-21
Anthropometric	SPLEEN	2.32	3.75228e-53
Anthropometric	WHLBLD	2.08	3.15448e-212
Anthropometric	LCL	2.05	3.55949e-09
Anthropometric	PNCREAS	2.03	2.78854e-05
Anthropometric	FIBRBLS	1.68	0.00125341
Anthropometric	SKINS	1.66	1.11094e-05
Anthropometric	ARTAORT	1.13	0.046682
Blood cell counts	BRNPTM	6.61	0.0357726
Blood cell counts	OVARY	3.72	3.0591e-08
Blood cell counts	PNCREAS	3.12	5.70102e-10
Blood cell counts	SKINS	3.03	1.20921e-20
Blood cell counts	CLNTRN	2.56	1.37593e-42
Blood cell counts	SPLEEN	2.08	3.98442e-25
Blood cell counts	FIBRBLS	2.06	0.000136569
Blood cell counts	LUNG	1.98	1.73542e-08
Blood cell counts	WHLBLD	1.96	1.04667e-109
Blood cell counts	SKINNS	1.70	2.55627e-07
Blood cell counts	HRTLV	1.59	0.00937432
Blood cell counts	STMACH	1.52	0.0205172
Blood cell counts	ADPVSC	1.35	0.029395
Breast cancer	BREAST	7.00	2.61877e-06
Breast cancer	STMACH	4.08	0.0384571
Breast cancer	CLNTRN	2.57	0.00465748
Breast cancer	TESTIS	2.21	0.0414983
Breast cancer	SPLEEN	2.15	0.0206049
Cardiometabolic	HRTAA	35.12	6.67643e-57
Cardiometabolic	ADRNLG	22.95	2.64047e-77
Cardiometabolic	OVARY	14.11	4.08608e-16
Cardiometabolic	LCL	9.39	1.05097e-29
Cardiometabolic	FIBRBLS	2.91	0.00454517
Cardiometabolic	WHLBLD	2.20	1.72301e-33
Cardiometabolic	ARTAORT	2.19	8.11519e-07
Cardiometabolic	CLNTRN	2.10	5.37462e-06
Cardiometabolic	SPLEEN	1.60	0.00480028
Endocrine system	THYROID	10.39	1.15429e-98
Endocrine system	SKINS	9.07	8.719e-30
Endocrine system	LIVER	7.01	0.000821877
Endocrine system	SKINNS	3.45	9.4903e-10

Disease category	Tissue	Enrichment	Enrichment p-value
Endocrine system	LCL	3.40	0.000200097
Endocrine system	PNCREAS	3.10	0.00846422
Endocrine system	CLNTRN	3.03	9.57363e-13
Endocrine system	BREAST	2.55	0.0007877
Endocrine system	ARTAORT	2.38	5.05314e-08
Endocrine system	BRNAMY	1.86	0.000643228
Endocrine system	SPLEEN	1.56	0.0176028
Hair morphology	STMACH	7.90	3.15107e-13
Hair morphology	ADPVSC	5.78	3.49335e-13
Hair morphology	WHLBLD	3.04	1.99356e-62
Hair morphology	CLNTRN	2.95	1.2905e-10
Immune	SKINS	7.62	8.3393e-48
Immune	BRNCHA	6.48	0.00111776
Immune	SKINNS	3.70	2.64663e-23
Immune	ADPVSC	3.46	1.65802e-11
Immune	CLNSGM	2.83	0.0131453
Immune	FIBRBLS	2.42	0.00251463
Immune	LUNG	2.33	6.95791e-06
Immune	PNCREAS	2.31	0.0095724
Immune	LCL	1.94	0.00704572
Immune	SPLEEN	1.69	2.12534e-05
Immune	ARTAORT	1.53	0.000588448
Immune	WHLBLD	1.29	8.22126e-06
Immune	UTERUS	1.24	0.0405764
Psychiatric-neurologic	SLVRYG	29.02	0.000332976
Psychiatric-neurologic	HRTLV	3.73	3.40141e-09
Psychiatric-neurologic	SKINNS	2.47	1.509e-09
Psychiatric-neurologic	WHLBLD	2.38	4.68601e-83
Psychiatric-neurologic	SPLEEN	1.95	6.73196e-09
Psychiatric-neurologic	LCL	1.80	0.0137359
Psychiatric-neurologic	SKINS	1.69	0.0292002
Skeletal system	SPLEEN	7.61	2.66551e-11
Skeletal system	MSCLSK	6.10	1.7921e-14

6.3 Supplementary results

Complementary to the Fig. 5b in our main text, we report all pairs of tissues and disease categories, which showed significant enrichment ($p < 0.05$) for trans-eQTLs to overlap with GWAS-associated SNPs, selected at a nominal cutoff of $p < 1 \times 10^{-6}$ (Table S2).

In Fig. S25, we report the GWAS enrichment for every tissue-study pair for the 87 disease phenotypes, at a nominal GWAS cutoff of $p \leq 1 \times 10^{-6}$. Traits without any significant GWAS-associated SNPs are not shown. Similar to disease categories in Fig. 5 in the main text, the GWAS enrichments of trans-eQTLs are specific to certain tissue-disease pairs. Some of these pairs suggest biological relationship, such as the enrichment of thyroid trans-eQTLs in hypothyroidism or the enrichment of adrenal gland trans-eQTLs in hypertension. There are many other such interesting physiological links, which merits a thorough analysis in the future.

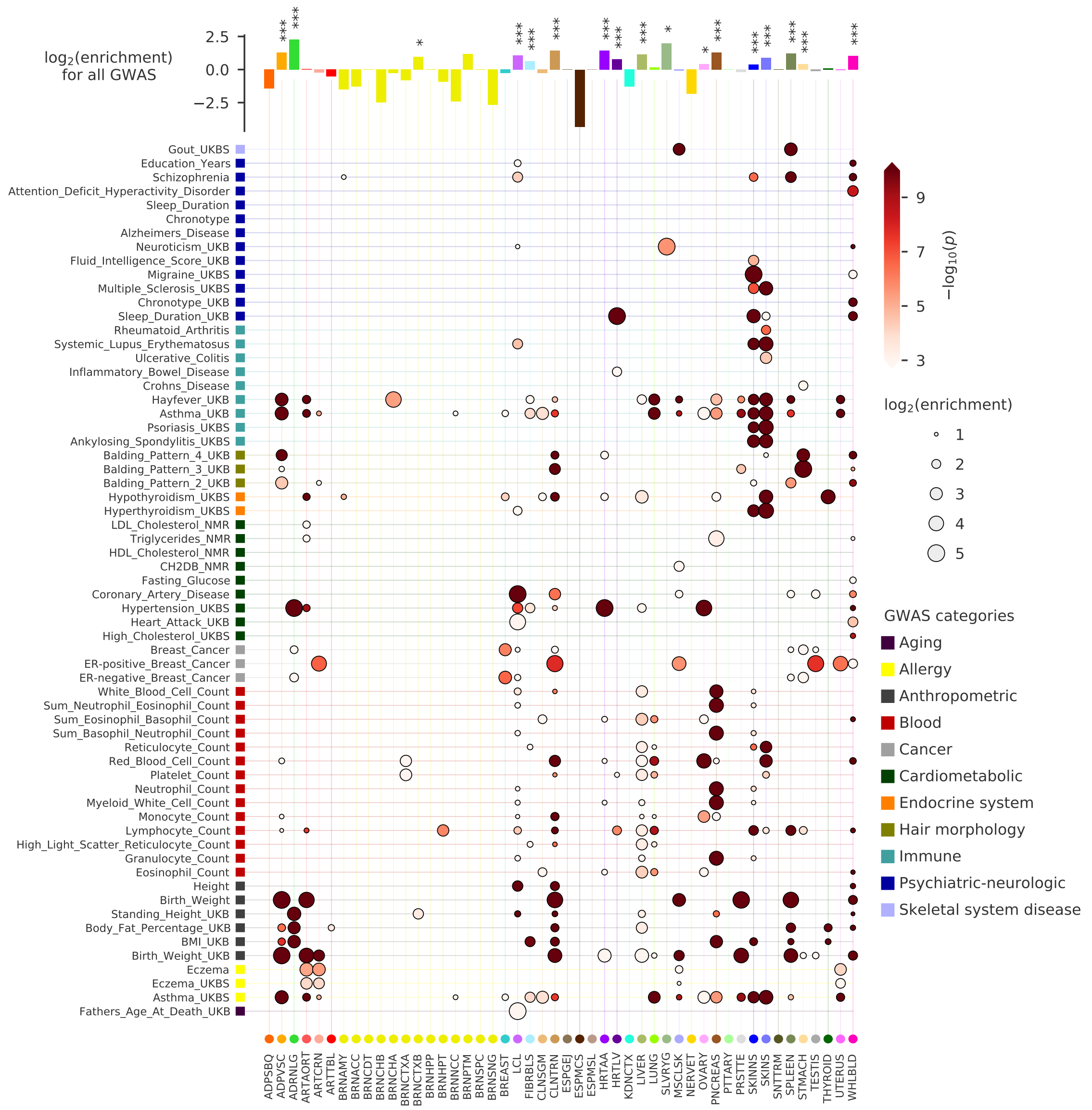

Fig. S25 | Overlap with GWAS-associated SNPs suggests tissue-specific regulation mechanism of trans-eQTLs. We calculated the enrichment of all trans-eQTLs predicted by Tejaas across all tissues (x-axis) in GWAS hits of 87 GWAS (y-axis). Each point in the plot represents the enrichment of trans-eQTLs of a tissue in a GWAS, the size of the point scales with the $\log_2$ enrichment, and the color scales with the significance ($-\log_{10}(p)$) of the enrichment. In the top panel, we show the GWAS enrichment for all studies combined.

However, in contrast to the GWAS enrichment of trans-eQTLs in GWAS catalog SNPs (Fig. 5a of main text), only a limited number of tissues show enrichment globally over all diseases combined (top panel of Fig. S25) in the dataset of complex trait GWAS. This is expected from this dataset as the constituent disease phenotypes are tissue-specific.

Appendix 1. Expectation and variance of RR-score under the null hypothesis

Here we show the derivation of the expectation value and the variance of q_{rev} (Eq. 19) under the permutation null model for any symmetric matrix $\mathbf{W}$ (Eq. 20) and any centered vector $\mathbf{x}$. We

rewrite q_{rev} as,

$$q_{\text{rev}} = \sum_{n,m=1}^{N} W_{nm}\, x_n x_m \,. \tag{75}$$

Expectation value of RR-score

We denote the permutation function of the indices between 1 and N as $\pi(\cdot)$ and we write $\mathbb{E}_\pi\left[\ldots\right]$ for the expectation value over multiple independent permutations. For example $\mathbb{E}_\pi\left[x_{\pi(n)}\right] = \sum_m x_m/N = 0$ where $x_{\pi(n)}$ is the nth entry of the vector $\mathbf{x}$ under permutation $\pi(\mathbf{x})$. For the expectation value of q_{rev} under random permutations we get

$$\mathbb{E}_\pi\left[\sum_{n,m=1}^{N} W_{nm}\, x_{\pi(n)} x_{\pi(m)}\right] = \sum_{n,m=1}^{N} W_{nm}\, \mathbb{E}_\pi\left[x_{\pi(n)} x_{\pi(m)}\right] \tag{76}$$

Their are only two values adopted by the expectation value $\mathbb{E}_\pi\left[x_{\pi(n)} x_{\pi(m)}\right]$, one in the case $n = m$ and the other in the case $n \neq m$. The first value is just $\mathbb{E}_\pi\left[x^2_{\pi(n)}\right] = (1/N)\sum_{n=1}^{N} x_n^2$, which is easily computed from $\mathbf{x}$. The value for the case $n \neq m$ is more tricky:

$$\begin{aligned}
\mathbb{E}_\pi\left[x_{\pi(n)} x_{\pi(m)}\right] &= \mathbb{E}_{n\neq m}\left[x_n x_m\right] \\
&= \frac{1}{N(N-1)} \sum_{n,m:n\neq m}^{N} x_n x_m \\
&= \frac{1}{N(N-1)} \left(\sum_{n,m}^{N} x_n x_m - \sum_{n=1}^{N} x_n^2\right) \\
&= \frac{1}{N(N-1)} \left(\left(\cancel{\sum_{n=1}^{N} x_n}\right)^2 - \sum_{n=1}^{N} x_n^2\right),
\end{aligned} \tag{77}$$

where the sum over x_n cancels out due to the centering of $\mathbf{x}$. We define the following moments of $\mathbf{x}$:

$$\begin{aligned}
\mu_2 &:= \frac{1}{N} \sum_{n=1}^{N} x_n^2 \\
\mu_4 &:= \frac{1}{N} \sum_{n=1}^{N} x_n^4
\end{aligned} \tag{78}$$

with which the expectation value for $n \neq m$ becomes

$$\mathbb{E}_\pi\left[x_{\pi(n)} x_{\pi(m)}\right] = -\frac{\mu_2}{N-1} \tag{79}$$

Inserting this into equation Eq. (76) yields

$$\mathbb{E}_\pi\left[\sum_{n,m=1}^{N} W_{nm}\, x_{\pi(n)} x_{\pi(m)}\right] = -\frac{\mu_2}{N-1} \sum_{n,m:n\neq m}^{N} W_{nm} + \mu_2 \sum_{n=1}^{N} W_{nn} \tag{80}$$

The first sum over W_{nm} can be expressed in terms of simpler sums,

$$\sum_{n,m:n\neq m}^{N} W_{nm} = \sum_{n,m=1}^{N} W_{nm} - \sum_{n=1}^{N} W_{nn} = w_{11} - w_2, \tag{81}$$

where we have defined the following moments of $\mathbf{W}$,

$$w_{11} := \sum_{n,m=1}^{N} W_{nm} \tag{82}$$

$$w_2 := \sum_{n=1}^{N} W_{nn}. \tag{83}$$

Inserting this into Eq. (80) yields

$$\langle q_{\mathrm{rev}} \rangle = \mathbb{E}_{\pi}\left[\sum_{n,m=1}^{N} W_{nm}\, x_{\pi(n)} x_{\pi(m)}\right] = \frac{\mu_2}{N-1}\left(N w_2 - w_{11}\right) . \tag{84}$$

Variance of RR-score

We will determine the variance with the equation

$$\mathrm{Var}\left[q_{\mathrm{rev}}\right] = \mathbb{E}_{\pi}\left[\left(\sum_{n,m=1}^{N} W_{nm}\, x_{\pi(n)} x_{\pi(m)}\right)^2\right] - \mathbb{E}_{\pi}\left[\sum_{n,m=1}^{N} W_{nm}\, x_{\pi(n)} x_{\pi(m)}\right]^2 , \tag{85}$$

and we therefore need to calculate the first term on the right hand side:

$$\mathbb{E}_{\pi}\left[\left(\sum_{n,m=1}^{N} W_{nm}\, x_{\pi(n)} x_{\pi(m)}\right)^2\right] = \sum_{n,m,n',m'=1}^{N} W_{nm} W_{n'm'}\, \mathbb{E}_{\pi}\left[x_{\pi(n)} x_{\pi(m)} x_{\pi(n')} x_{\pi(m')}\right] . \tag{86}$$

The term $\mathbb{E}_{\pi}\left[x_{\pi(n)} x_{\pi(m)} x_{\pi(n')} x_{\pi(m')}\right]$ can only take five different values, depending on how many of the four indices are identical to each other. To simplify the following, quite long derivation of the variance of q_{rev}, we will introduce some notation. First, to simplify writing down sums over two, three or four mutually different index variables, we define sets

$$\begin{aligned}
\mathcal{N}_2 &:= \{(n,m) : 1 \leq n,m \leq N, n \neq m\} \\
\mathcal{N}_3 &:= \{(n,m,n') : 1 \leq n,m,n' \leq N, n \neq m, n \neq n', m \neq m'\} \\
\mathcal{N}_4 &:= \{(n,m,n',m') : 1 \leq n,m,n',m' \leq N, n \neq m, n \neq n', \ldots, n' \neq m'\} .
\end{aligned} \tag{87}$$

We denote by $\mathbb{E}_{(n.m)\in\mathcal{N}_2}[\cdot]$ or simply $\mathbb{E}_{\mathcal{N}_2}[\cdot]$ the expectation value over a random variable $(n,m) \in \mathcal{N}_2$, and analogously for $\mathbb{E}_{\mathcal{N}_3}[\cdot]$ and $\mathbb{E}_{\mathcal{N}_4}[\cdot]$. We further abbreviate

$$\begin{aligned}
v_{1111} &= \mathbb{E}_{\mathcal{N}_4}\left[x_n x_m x_{n'} x_{m'}\right] \\
v_{211} &= \mathbb{E}_{\mathcal{N}_3}\left[x_n^2 x_m x_{n'}\right] \\
v_{22} &= \mathbb{E}_{\mathcal{N}_2}\left[x_n^2 x_m^2\right] \\
v_{31} &= \mathbb{E}_{\mathcal{N}_2}\left[x_n^3 x_m\right] .
\end{aligned} \tag{88}$$

With these definitions, Eq. (86) becomes

$$
\begin{aligned}
&\mathbb{E}_\pi \left[\left(\sum_{n,m=1}^{N} W_{nm}\, x_{\pi(n)} x_{\pi(m)} \right)^2 \right] \\
&\quad = \nu_{1111} \sum_{(n,m,n',m') \in \mathcal{N}_4} W_{nm} W_{n'm'} \\
&\quad + \nu_{211} \sum_{(n,m,n') \in \mathcal{N}_3} \left(W_{nn} W_{mn'} + W_{nm} W_{n'n'} + W_{nm} W_{nn'} + W_{nm} W_{n'n} + W_{nm} W_{mn'} + W_{nm} W_{n'm} \right) \\
&\quad + \nu_{22} \sum_{(n,m) \in \mathcal{N}_2} \left(W_{nn} W_{mm} + W_{nm} W_{nm} + W_{nm} W_{mn} \right) \\
&\quad + \nu_{31} \sum_{(n,m) \in \mathcal{N}_2} \left(W_{nn} W_{nm} + W_{nn} W_{mn} + W_{nm} W_{nn} + W_{nm} W_{mm} \right) \\
&\quad + \mu_4 \sum_{n=1}^{N} W_{nn}^2
\end{aligned}
\tag{89}
$$

and, using the symmetry of $\mathbf{W}$,

$$
\begin{aligned}
\mathbb{E}_\pi \left[\left(\sum_{n,m=1}^{N} W_{nm}\, x_{\pi(n)} x_{\pi(m)} \right)^2 \right] &= \nu_{1111} \sum_{(n,m,n',m') \in \mathcal{N}_4} W_{nm} W_{n'm'} \\
&+ \nu_{211} \sum_{(n,m,n') \in \mathcal{N}_3} \left(2 W_{nn} W_{mn'} + 4 W_{nm} W_{nn'} \right) \\
&+ \nu_{22} \sum_{(n,m) \in \mathcal{N}_2} \left(W_{nn} W_{mm} + 2 W_{nm} W_{nm} \right) \\
&+ \nu_{31} \sum_{(n,m) \in \mathcal{N}_2} 4 W_{nn} W_{nm} + \mu_4 \sum_{n=1}^{N} W_{nn}^2
\end{aligned}
\tag{90}
$$

We will first derive expressions for the ν terms and then for the sums of elements of $\mathbf{W}$.

$$
\begin{aligned}
\nu_{31} &= \mathbb{E}_{\mathcal{N}_2} \left[x_n^3 x_m \right] \\
&= \frac{1}{N(N-1)} \sum_{(n,m) \in \mathcal{N}_2} x_n^3 x_m \\
&= \frac{1}{N(N-1)} \left(\cancel{\sum_{n,m=1}^{N} x_n^3 x_m} - \sum_{n=1}^{N} x_n^4 \right) \\
&= -\frac{1}{N-1} \mu_4
\end{aligned}
\tag{91}
$$

$$
\begin{aligned}
\nu_{22} &= \mathbb{E}_{\mathcal{N}_2} \left[x_n^2 x_m^2 \right] \\
&= \frac{1}{N(N-1)} \sum_{(n,m) \in \mathcal{N}_2} x_n^2 x_m^2 \\
&= \frac{1}{N(N-1)} \left(\sum_{n,m=1}^{N} x_n^2 x_m^2 - \sum_{n=1}^{N} x_n^4 \right) \\
&= \frac{N}{N-1} \mu_2^2 - \frac{1}{N-1} \mu_4
\end{aligned}
\tag{92}
$$

$$
\begin{aligned}
\nu_{211} &= \mathbb{E}_{\mathcal{N}_3}\left[x_n^2 x_m x_{n'}\right] \\
&= \frac{1}{N(N-1)(N-2)} \sum_{(n,m,n')\in\mathcal{N}_3} x_n^2 x_m x_{n'} \\
&= \frac{1}{N(N-1)(N-2)} \sum_{(n,m)\in\mathcal{N}_2} \left(\cancel{\sum_{n'=1}^{N} x_n^2 x_m x_{n'}} - x_n^3 x_m - x_n^2 x_m^2 \right) \\
&= -\frac{1}{N-2}\left(\nu_{31} + \nu_{22}\right) \\
&= -\frac{1}{(N-1)(N-2)}\left(N\mu_2^2 - 2\mu_4\right)
\end{aligned}
\tag{93}
$$

$$
\begin{aligned}
\nu_{1111} &= \mathbb{E}_{\mathcal{N}_3}\left[x_n x_m x_{n'} x_{m'}\right] \\
&= \frac{1}{N(N-1)(N-2)(N-3)} \sum_{(n,m,n',m')\in\mathcal{N}_4} x_n x_m x_{n'} x_{m'} \\
&= \frac{1}{N(N-1)(N-2)(N-3)} \sum_{(n,m,n')\in\mathcal{N}_3} \left(\cancel{\sum_{m'=1}^{N} x_n x_m x_{n'} x_{m'}} - x_n^2 x_m x_{n'} - x_n x_m^2 x_{n'} - x_n x_m x_{n'}^2 \right) \\
&= -\frac{3}{N-3}\nu_{211} \\
&= \frac{3}{(N-1)(N-2)(N-3)}\left(N\mu_2^2 - 2\mu_4\right)
\end{aligned}
\tag{94}
$$

and, in summary,

$$
\begin{aligned}
\nu_{31} &= -\frac{1}{N-1}\mu_4 \\
\nu_{22} &= \frac{N}{N-1}\mu_2^2 + \nu_{31} \\
\nu_{211} &= -\frac{1}{N-2}\left(\nu_{31} + \nu_{22}\right) \\
\nu_{1111} &= -\frac{3}{N-3}\nu_{211}\,.
\end{aligned}
\tag{95}
$$

We will now derive expressions for the sums of elements of $\mathbf{W}$ in equation (90):

$$
\sum_{(n,m)\in\mathcal{N}_2} W_{nn} W_{nm} = \sum_{n,m=1}^{N} W_{nn} W_{nm} - \sum_{n=1}^{N} W_{nn}^2 = w_{31} - w_4 \tag{96}
$$

where

$$
\begin{aligned}
w_{31} &:= \sum_{n,m=1}^{N} W_{nn} W_{nm} = \sum_{n=1}^{N} W_{nn} W_{n}. \\
w_4 &:= \sum_{n=1}^{N} W_{nn}^2 \\
w_{22} &:= \sum_{n,m=1}^{N} W_{nm}^2
\end{aligned}
$$

$$w_{211} := \sum_{n,m,n'=1}^{N} W_{nm}W_{nn'} = \sum_{n=1}^{N}\left(\sum_{m=1}^{N} W_{nm}\right)^2 = \sum_{n=1}^{N} W_{n\cdot}^2. \tag{97}$$

Also

$$\sum_{(n,m)\in\mathcal{N}_2} W_{nn}W_{mm} = \sum_{n,m=1}^{N} W_{nn}W_{mm} - \sum_{n=1}^{N} W_{nn}^2 = w_2^2 - w_4 \tag{98}$$

$$\sum_{(n,m)\in\mathcal{N}_2} W_{nm}W_{nm} = \sum_{n,m=1}^{N} W_{nm}W_{nm} - \sum_{n=1}^{N} W_{nn}^2 = w_{22} - w_4 \tag{99}$$

$$\begin{aligned}
\sum_{(n,m,n')\in\mathcal{N}_3} W_{nn}W_{mn'} &= \sum_{(n,m)\in\mathcal{N}_2}\sum_{n'=1}^{N} W_{nn}W_{mn'} - \sum_{(n,m)\in\mathcal{N}_2}(W_{nn}W_{mn} + W_{nn}W_{mm}) \\
&= \sum_{(n,m)\in\mathcal{N}_2}\sum_{n'=1}^{N} W_{nn}W_{mn'} - \sum_{n,m=1}^{N}(W_{nn}W_{mn} + W_{nn}W_{mm}) + 2w_4 \\
&= \sum_{n,m,n'=1}^{N} W_{nn}W_{mn'} - \sum_{n,n'=1}^{N} W_{nn}W_{nn'} - w_{31} - \sum_{n=1}^{N} W_{nn}\sum_{m=1}^{N} W_{mm} + 2w_4 \\
&= \sum_{n=1}^{N} W_{nn}\sum_{m,n'=1}^{N} W_{mn'} - w_{31} - w_{31} - w_2^2 + 2w_4 \\
&= w_2\, w_{11} - 2w_{31} - w_2^2 + 2w_4
\end{aligned} \tag{100}$$

$$\begin{aligned}
\sum_{(n,m,n')\in\mathcal{N}_3} W_{nm}W_{nn'} &= \sum_{(n,m)\in\mathcal{N}_2}\sum_{n'=1}^{N} W_{nm}W_{nn'} - \sum_{n,m=1}^{N}(W_{nm}W_{nm} + W_{nm}W_{nn}) + 2w_4 \\
&= \sum_{n,m=1}^{N}\sum_{n'=1}^{N} W_{nm}W_{nn'} - \sum_{n=1}^{N}\sum_{n'=1}^{N} W_{nn}W_{nn'} - w_{22} - w_{31} + 2w_4 \\
&= (w_{211} - w_{31}) - w_{22} - w_{31} + 2w_4 \\
&= w_{211} - 2w_{31} - w_{22} + 2w_4
\end{aligned} \tag{101}$$

$$\begin{aligned}
\sum_{(n,m,n',m')\in\mathcal{N}_4} W_{nm}W_{n'm'} &= \sum_{(n,m,n')\in\mathcal{N}_3}\sum_{m'=1}^{N} W_{nm}W_{n'm'} - \sum_{(n,m,n')\in\mathcal{N}_3}(W_{nm}W_{n'n} + W_{nm}W_{n'm} + W_{nm}W_{n'n'}) \\
&= \sum_{(n,m,n')\in\mathcal{N}_3}\sum_{m'=1}^{N} W_{nm}W_{n'm'} - 2\sum_{(n,m,n')\in\mathcal{N}_3} W_{nm}W_{nn'} - \sum_{(n,m,n')\in\mathcal{N}_3} W_{nn}W_{mn'} \\
&\overset{(101)(100)}{=} \sum_{(n,m)\in\mathcal{N}_2}\left(\sum_{n',m'=1}^{N} W_{nm}W_{n'm'} - \sum_{m'=1}^{N}(W_{nm}W_{nm'} + W_{nm}W_{mm'})\right) \\
&\quad - 2(w_{211} - 2w_{31} - w_{22} + 2w_4) - (w_2\, w_{11} - 2w_{31} - w_2^2 + 2w_4) \\
&\overset{(81)}{=} (w_{11} - w_2)\, w_{11} - 2\sum_{(n,m)\in\mathcal{N}_2}\sum_{m'=1}^{N} W_{nm}W_{nm'} \\
&\quad - 2(w_{211} - 2w_{31} - w_{22} + 2w_4) - (w_2\, w_{11} - 2w_{31} - w_2^2 + 2w_4) \\
&\overset{(101)}{=} (w_{11} - w_2)\, w_{11} - 2(w_{211} - w_{31}) \\
&\quad - 2(w_{211} - 2w_{31} - w_{22} + 2w_4) - (w_2\, w_{11} - 2w_{31} - w_2^2 + 2w_4) \\
&= w_{11}^2 - 2w_2\, w_{11} - 4w_{211} + 8w_{31} + 2w_{22} + w_2^2 - 6w_4
\end{aligned} \tag{102}$$

We can check that the last term is correct by checking if the number of $W_{nm}W_{n'm'}$ terms in the last line is equal to $N(N-1)(N-2)(N-3) = N^4 - 6N^3 + 11N^2 - 6N$. To do that, we check that each of the w terms sums up N^k terms, where k is the number of digits in its subscript. For example w_4 and w_2 sum up N terms, w_{11} and w_{22} and w_{31} sum up N^2, and w_{211} sums up N^3. The last line of the previous equation therefore sums up $N^4-2NN^2-4N^3+8N^2+2N^2+N^2-6N = N^4-6N^3+11N^2-6N$ terms, which gives exactly what it should. Doing that with the other terms also checks. We insert the expressions for the sums over $\mathbf{W}$ terms into Eq. (90) and obtain

$$\begin{aligned}\mathbb{E}_\pi\left[\left(\sum_{n,m=1}^{N} W_{nm}\, x_{\pi(n)} x_{\pi(m)}\right)^2\right] &= \nu_{1111}\left(w_{11}^2 - 2w_2\, w_{11} - 4w_{211} + 8w_{31} + 2w_{22} + w_2^2 - 6w_4\right) \\ &+ \nu_{211}\left(2(w_2\, w_{11} - 2w_{31} - w_2^2 + 2w_4) + 4(w_{211} - 2w_{31} - w_{22} + 2w_4)\right) \\ &+ \nu_{22}\left((w_2^2 - w_4) + 2(w_{22} - w_4)\right) \\ &+ 4\,\nu_{31}\left(w_{31} - w_4\right) + \mu_4 w_2 \end{aligned} \tag{103}$$

and, by adding up terms with the same w moments, finally

$$\begin{aligned}\mathbb{E}_\pi\left[\left(\sum_{n,m=1}^{N} W_{nm}\, x_{\pi(n)} x_{\pi(m)}\right)^2\right] &= \nu_{1111}\left(w_{11}^2 - 2w_2\, w_{11} - 4w_{211} + 8w_{31} + 2w_{22} + w_2^2 - 6w_4\right) \\ &+ 2\,\nu_{211}\left(w_2\, w_{11} + 2w_{211} - 6w_{31} - 2w_{22} - w_2^2 + 6w_4\right) \\ &+ \nu_{22}\left(w_2^2 + 2w_{22} - 3w_4\right) + 4\,\nu_{31}\left(w_{31} - w_4\right) + \mu_4 w_4 \end{aligned} \tag{104}$$

Appendix 2. Abbreviation of GTEx tissues

Tissue	Abbreviation
Adipose - Subcutaneous	ADPSBQ
Adipose - Visceral (Omentum)	ADPVSC
Adrenal Gland	ADRNLG
Artery - Aorta	ARTAORT
Artery - Coronary	ARTCRN
Artery - Tibial	ARTTBL
Brain - Amygdala	BRNAMY
Brain - Anterior cingulate cortex (BA24)	BRNACC
Brain - Caudate (basal ganglia)	BRNCDT
Brain - Cerebellar Hemisphere	BRNCHB
Brain - Cerebellum	BRNCHA
Brain - Cortex	BRNCTXA
Brain - Frontal Cortex (BA9)	BRNCTXB
Brain - Hippocampus	BRNHPP
Brain - Hypothalamus	BRNHPT
Brain - Nucleus accumbens (basal ganglia)	BRNNCC
Brain - Putamen (basal ganglia)	BRNPTM
Brain - Spinal cord (cervical c-1)	BRNSPC
Brain - Substantia nigra	BRNSNG
Breast - Mammary Tissue	BREAST
Cells - EBV-transformed lymphocytes	LCL
Colon - Sigmoid	CLNSGM
Colon - Transverse	CLNTRN
Esophagus - Gastroesophageal Junction	ESPGEJ
Esophagus - Mucosa	ESPMCS
Esophagus - Muscularis	ESPMSL
Heart - Atrial Appendage	HRTAA
Heart - Left Ventricle	HRTLV
Kidney - Cortex	KDNCTX
Liver	LIVER
Lung	LUNG
Minor Salivary Gland	SLVRYG
Muscle - Skeletal	MSCLSK
Nerve - Tibial	NERVET
Ovary	OVARY
Pancreas	PNCREAS
Pituitary	PTTARY
Prostate	PRSTTE
Skin - Not Sun Exposed (Suprapubic)	SKINNS
Skin - Sun Exposed (Lower leg)	SKINS
Small Intestine - Terminal Ileum	SNTTRM
Spleen	SPLEEN
Stomach	STMACH
Testis	TESTIS
Thyroid	THYROID
Uterus	UTERUS
Vagina	VAGINA
Whole Blood	WHLBLD

Appendix 3. eQTLGen Replication

Tissue	Replicated trans-eQTLs	Enrichment
**Whole Blood**	**37 (0.96%)**	**3.16 ($p = 2.99012e^{-09}$)**
Adipose Subcutaneous	0 (0.00%)	-
Adipose Visceral Omentum	1 (0.03%)	1.45 (p = 0.498496)
Adrenal Gland	3 (0.08%)	8.33 (p = 0.00593454)
Artery Aorta	2 (0.05%)	1.03 (p = 0.577555)
Artery Coronary	3 (0.08%)	1.08 (p = 0.5258)
Artery Tibial	0 (0.00%)	-
Brain Amygdala	0 (0.00%)	-
Brain Anterior cingulate cortex BA24	2 (0.05%)	1.41 (p = 0.415078)
Brain Caudate basal ganglia	0 (0.00%)	-
Brain Cerebellar Hemisphere	0 (0.00%)	-
Brain Cerebellum	0 (0.00%)	-
Brain Cortex	0 (0.00%)	-
Brain Frontal Cortex BA9	0 (0.00%)	-
Brain Hippocampus	0 (0.00%)	-
Brain Hypothalamus	0 (0.00%)	-
Brain Nucleus accumbens basal ganglia	0 (0.00%)	-
Brain Putamen basal ganglia	0 (0.00%)	-
Brain Spinal cord cervical c-1	0 (0.00%)	-
Brain Substantia nigra	0 (0.00%)	-
Breast Mammary Tissue	3 (0.08%)	4.55 (p = 0.0294283)
Cells EBV-transformed lymphocytes	2 (0.05%)	5.00 (p = 0.061526)
Cells Cultured fibroblasts	1 (0.03%)	5.00 (p = 0.181291)
Colon Sigmoid	1 (0.03%)	7.14 (p = 0.130668)
Colon Transverse	9 (0.23%)	4.29 (p = 0.00033596)
Esophagus Gastroesophageal Junction	0 (0.00%)	-
Esophagus Mucosa	0 (0.00%)	-
Esophagus Muscularis	0 (0.00%)	-
Heart Atrial Appendage	1 (0.03%)	5.88 (p = 0.156371)
Heart Left Ventricle	2 (0.05%)	7.14 (p = 0.0325744)
Kidney Cortex	0 (0.00%)	-
Liver	0 (0.00%)	-
Lung	2 (0.05%)	2.82 (p = 0.15927)
Minor Salivary Gland	0 (0.00%)	-
Muscle Skeletal	4 (0.10%)	0.92 (p = 0.63004)
Nerve Tibial	0 (0.00%)	-
Ovary	1 (0.03%)	12.50 (p = 0.0768929)
Pancreas	0 (0.00%)	-
Pituitary	0 (0.00%)	-
Prostate	2 (0.05%)	1.39 (p = 0.421922)
Skin Not Sun Exposed Suprapubic	3 (0.08%)	3.12 (p = 0.0730636)
Skin Sun Exposed Lower leg	3 (0.08%)	5.45 (p = 0.0184417)
Small Intestine Terminal Ileum	1 (0.03%)	9.09 (p = 0.104185)
Spleen	1 (0.03%)	0.47 (p = 0.882392)
Stomach	1 (0.03%)	2.94 (p = 0.288275)
Testis	2 (0.05%)	0.96 (p = 0.617861)
Thyroid	0 (0.00%)	-
Uterus	3 (0.08%)	0.93 (p = 0.622227)
Vagina	0 (0.00%)	-

References

[1] Brynedal, B. *et al.* Large-scale trans-eQTLs affect hundreds of transcripts and mediate patterns of transcriptional co-regulation. *The American Journal of Human Genetics* **100**, 581–591 (2017). URL http://dx.doi.org/10.1016/j.ajhg.2017.02.004.

[2] Li, B. & Leal, S. M. Methods for detecting associations with rare variants for common diseases: application to analysis of sequence data. *The American Journal of Human Genetics* **83**, 311–321 (2008). URL https://doi.org/10.1016/j.ajhg.2008.06.024.

[3] Wu, M. C. *et al.* Rare-variant association testing for sequencing data with the sequence kernel association test. *The American Journal of Human Genetics* **89**, 82–93 (2011). URL https://doi.org/10.1016/j.ajhg.2011.05.029.

[4] Ionita-Laza, I., Lee, S., Makarov, V., Buxbaum, J. D. & Lin, X. Sequence kernel association tests for the combined effect of rare and common variants. *The American Journal of Human Genetics* **92**, 841–853 (2013). URL https://doi.org/10.1016/j.ajhg.2013.04.015.

[5] Stegle, O., Leopold, P., Richard, D. & John, W. A Bayesian framework to account for complex non-genetic factors in gene expression levels greatly increases power in eQTL studies. *PLOS Computational Biology* **6**, 1–11 (2010). URL https://doi.org/10.1371/journal.pcbi.1000770.

[6] Manor, O. & Eran, S. Robust prediction of expression differences among human individuals using only genotype information. *PLOS Genetics* **9**, 1–14 (2013). URL https://doi.org/10.1371/journal.pgen.1003396.

[7] Dasarathy, B. V. *Nearest neighbor (NN) norms: nn pattern classification techniques*. IEEE Computer Society Press tutorial (IEEE Computer Society Press, 1991). URL https://books.google.de/books?id=k2dQAAAAMAAJ.

[8] Hore, V. *et al.* Tensor decomposition for multiple-tissue gene expression experiments. *Nature Genetics* **48**, 1094–1100 (2016). URL https://doi.org/10.1038/ng.3624.

[9] Lonsdale, J. *et al.* The genotype-tissue expression (GTEx) project. *Nature Genetics* **45**, 580–585 (2013). URL https://doi.org/10.1038/ng.2653.

[10] GTEx Consortium. The genotype-tissue expression (GTEx) pilot analysis: Multitissue gene regulation in humans. *Science* **348**, 648–660 (2015). URL https://doi.org/10.1126/science.1262110.

[11] Aguet, F. *et al.* Genetic effects on gene expression across human tissues. *Nature* **550**, 204–213 (2017). URL https://doi.org/10.1038/nature24277.

[12] Small, K. S. *et al.* Identification of an imprinted master trans regulator at the klf14 locus related to multiple metabolic phenotypes. *Nature Genetics* **43**, 561–564 (2011). URL https://doi.org/10.1038/ng.833.

[13] GTEx Consortium. The GTEx consortium atlas of genetic regulatory effects across human tissues. *Science* **369**, 1318 (2020). URL http://science.sciencemag.org/content/369/6509/1318.abstract.

[14] Liu, X., Li, Y. I. & Pritchard, J. K. Trans effects on gene expression can drive omnigenic inheritance. *Cell* **177**, 1022–1034 (2019). URL https://doi.org/10.1016/j.cell.2019.04.014.

[15] Shabalin, A. A. Matrix eQTL: ultra fast eQTL analysis via large matrix operations. *Bioinformatics (Oxford, England)* **28**, 1353–1358 (2012). URL https://doi.org/10.1093/bioinformatics/bts163.

[16] Robinson, M. D. & Oshlack, A. A scaling normalization method for differential expression analysis of RNA-seq data. *Genome Biology* **11**, R25 (2010). URL https://doi.org/10.1186/gb-2010-11-3-r25.

[17] GTEx portal ©2019 The Broad Institute of MIT and Harvard. https://gtexportal.org/home. URL https://gtexportal.org/home. [Accessed: 10-March-2020].

[18] Saha, A. & Battle, A. False positives in trans-eQTL and co-expression analyses arising from RNA-sequencing alignment errors. *F1000Research* **7**, 1860 (2018). URL https://doi.org/10.12688/f1000research.17145.2.

[19] Weir, B. S. & Cockerham, C. C. Estimating f-statistics for the analysis of population structure. *Evolution* **38**, 1358–1370 (1984). URL https://doi.org/10.2307/2408641.

[20] Bhatia, G., Patterson, N., Sankararaman, S. & Price, A. L. Estimating and interpreting fst: The impact of rare variants. *Genome Res* **23**, 1514–1521 (2013). URL https://doi.org/10.1101/gr.154831.113.

[21] Thurman, R. E. *et al.* The accessible chromatin landscape of the human genome. *Nature* **489**, 75–82 (2012). URL http://dx.doi.org/10.1038/nature11232.

[22] NHGRI-EBI. GWAS catalog. https://www.ebi.ac.uk/gwas/ (2019). [Accessed 24-February-2020].

[23] MacArthur, J. *et al.* The new NHGRI-EBI catalog of published genome-wide association studies (GWAS catalog). *Nucleic Acids Research* **45**, 896–901 (2016). URL https://dx.doi.org/10.1093/nar/gkw1133.

[24] NHGRI-EBI. GWAS catalog inclusion criteria. https://www.ebi.ac.uk/gwas/docs/methods/criteria (2017). URL https://www.ebi.ac.uk/gwas/docs/methods/criteria. [Accessed 13-May-2020].

[25] Barbeira, A. N. *et al.* Exploiting the gtex resources to decipher the mechanisms at gwas loci. *Genome Biology* **22**, 49 (2021). URL https://doi.org/10.1186/s13059-020-02252-4.
