# Supplementary material for: Tejaas: reverse regression increases power for detecting trans-eQTLs
Source: Genome Biol. 2021 May 6;22:142. doi: 10.1186/s13059-021-02361-8 (PMC8101255; doi:10.1186/s13059-021-02361-8)
Supplement: Supplementary file 2 — Additional file 2 Review history [file 13059_2021_2361_MOESM2_ESM.docx]

Review History

**First round of review**

**Reviewer 1**

**Were you able to assess all statistics in the manuscript, including the appropriateness of statistical tests used?**

Yes: There are important questions regarding the appropriateness of the statistical methods used, as noted in the comments to the author.

**Were you able to directly test the methods?**

No

**Comments to author:**

The manuscript by Banerjee and colleagues introduces a regularization-based method to identify trans-eQTLs. The authors employ a reverse-regression with L2 regularization to cumulate weaker effects across many genes and thus detect which genetic variant(s) harbors at least one non-zero association with a distal gene-expression. In the process, the authors have introduced a number of newer approaches to supplement the standard trans-eQTL discovery like estimation of regularization parameters, kNN method of covariate adjustment etc. The results show that the derived method can potentially detect weaker trans-eQTL both in simulations and in GTEx data. Some further exploration is necessary to ensure the results are accurate:

1. The authors focus on identifying the genetic variants/loci which has an association with at least one distant gene. However, the method does not output which or how many genes are associated with the variant. This can restrict the applicability of the method since mapping these detected trans-eQTLs to target genes is one of the main goals of trans-eQTL mapping and informs downstream consequences. Though, this is not the principal goal of the paper as the authors point out in supplementary, but I think identifying trans-eQTLs without identifying the target genes stifles greatly the use of the method. In the current implementation there's an option to extract the SNP-gene association after the trans-eQTLs have been identified, but it is not clear how to declare which of them are significant, i.e., which are the target genes.

2. The authors claim that the null distribution for the test statistic can be approximated by a normal distribution with an estimated mean and variance for which the authors provide an analytical form. Additionally, they have shown that the analytically estimated mean and variance of the test statistics agree well with that from permutation-based estimates. However, the authors need to additionally evaluate whether the normality assumption is valid or not through comparisons of qq-plot (or otherwise) especially in the extreme tails (~5e-08). Slightest deviations from the normality assumption can result in substantial impact on the output. This is a critical addition, and could change the conclusions significantly if the tails are not well modeled.

3. Questions also arise in the choice to model a non-negative test statistic through normal distribution, which could also lead to incorrect conclusions. I believe the model framework and hypothesis of inference is exactly similar as the SKAT model (aggregating effects across multiple variants). The authors should test a variance-component based score test, which could be computationally efficient and the modelling test statistic as a summation of chi-squares null distribution has been proven to work well in moderate sample sizes and high significance levels (~2.5e-06).

4. I don't fully agree with the statement in the Discussion section that "…All trans-eQTLs identified to date, including the meta-analysis on whole blood with 31,684 individuals, were discovered by strong effects on a single, distant gene. Hence, by design, Tejaas might not replicate these existing trans-eQTLs with statistical significance…". The authors claim that their method is designed to aggregate weaker effects to identify trans-eQTLs. In eQTLGen, due to higher power resulting from much higher sample size, the standard trans-mapping is able to capture many weaker effects which Tejaas "might be" able to capture in a smaller sample size as in GTEx. I believe this statement was motivated by the very small overlap of the trans-eQTLs detected via Tejaas and eQTLGen. But since even such small overlap is much more than expected (enriched), this strengthens the method's properties. Furthermore, there are existing papers that aggregate signal to identify trans-eQTLs, including PMC5384037, PMC7444084 and others.

5. In Simulations, authors have used a gamma effect size distribution. However, we need to see how well this replicates weaker effects normally seen in trans-eQTLs. The authors should quantify average number of significant variant-gene associations obtained from such effect size distributions to calibrate the expectation from standard trans-mapping. Also, it would be good to get a sense of the heritability of gene-expressions explained by such effects since it is estimated that about 70% of gene-expression heritability is trans-regulated. Does the simulation scheme reflect that?

6. I find the enrichment of the detected trans-eQTLs among GWAS variants rather weak which might be caused by false/noisy signals captured by Tejaas? Further in Fig 3, the number of trans-eQTLs without cis-effect in several tissues like Blood and Muscle looks rather high. Can the author's comment if that's a real finding or would it be prone to errors.

7. The increase in trans-eQTLs after cross-mapping filtering is unusual and should be double checked and explained. No p-values should have changed and only a minority of candidate tests would be filtered, so the multiple hypothesis correction should not be affected dramatically. In response to the review, please provide examples of hits that were filtered by this step, and hits that were brought in by this step.

8. Relatedly the authors should provide the significant SNPs and potential explanatory genes for all data sets in a supplementary table.

**Reviewer 2**

**Were you able to assess all statistics in the manuscript, including the appropriateness of statistical tests used?**

No

**Were you able to directly test the methods?**

No

**Comments to author:**

This manuscript describes a method to detect trans-eQTLs by an L2-regularized multiple regression. Although it looks like an interesting manuscript, this review has some questions and comments on the study.

1. It seems to me that the methods' limitation of being unable to identify the target genes of a discovered trans-eQTL substantially diminish its practicability. In the genetics community, what we pressingly need is to find out all possible trans-eQTLs of a gene in a given tissue context, but not to merely know whether a SNP is a trans-eQTLs for some genes in some tissues. Once a SNP is a significant trans-eQTLs by the proposed method, one still has to use conventional regression to link the variant to one or more genes. The correlation of genes will always confuse the determination of functionally linked genes of a trans-eQTL.

2. The motivation of the reverse regression is not very convincing. The multiple regression is not good at handling covariates with correlation, and the collinearity may make the model unstable.

3. A more problematic issue is the "curse of dimensionality". You may have thousands of genes in around 50 tissues, which is a vast dimension. How the L2-regularized is sufficient for this issue in practices is doubtable.

4. What the Forward Regression does is the analysis of p-values at all genes for a SNP. So both the Forward Regression and revers regression have similar multiple testing burden.

5. In the methods section, "We model x with a univariate normal distribution…", x is just a standardized genotype code and discrete. Maybe it is not suitable to assume it follows the normal distribution.

6. MatrixEQTL and FR were both used to compare with the proposed method in simulation studies, and the proposed method turned out to be best. What is the comparison performed in a real dataset?

7. In simulation studies, why the correction methods were different (i.e. For MatrixEQTL and FR, we chose the CCLM correction and for Tejaas, the KNN correction.)?

8. The authors estimated trans-eQTLs in most of the GTEx tissues. I wonder if others have reported these findings? I also wonder if these findings can be validated in an independent dataset.

9. In Figure 4, there was no picture explanation for Figure 4a.

**Authors Response**

**Point-by-point responses to the reviewers’ comments:**

Comments by Referee 1 (GBIO-D-20-01439)

The manuscript by Banerjee and colleagues introduces a regularization-based method to identify trans-eQTLs. The authors employ a reverse-regression with L2 regularization to cumulate weaker effects across many genes and thus detect which genetic variant(s) harbors at least one non-zero association with a distal gene-expression. In the process, the authors have introduced a number of newer approaches to supplement the standard trans-eQTL discovery like estimation of regularization parameters, kNN method of covariate adjustment etc. The results show that the derived method can potentially detect weaker trans-eQTL both in simulations and in GTEx data. Some further exploration is necessary to ensure the results are accurate:

1.	The authors focus on identifying the genetic variants/loci which has an association with at least one distant gene. However, the method does not output which or how many genes are associated with the variant. This can restrict the applicability of the method since mapping these detected trans-eQTLs to target genes is one of the main goals of trans-eQTL mapping and informs downstream consequences. Though, this is not the principal goal of the paper as the authors point out in supplementary, but I think identifying trans-eQTLs without identifying the target genes stifles greatly the use of the method. In the current implementation there's an option to extract the SNP-gene association after the trans-eQTLs have been identified, but it is not clear how to declare which of them are significant, i.e., which are the target genes.

*We now include Benjamini-Hochberg false discovery rates (FDR) for all trans target genes identified with an FDR below 50% (or any threshold specified by --fdrgenethres), for all predicted trans eQTL SNPs with P-value below 5 × 10-8 (or any threshold specified by --psnpthres). This is discussed in Supplementary Sec. 2.9.*

*We have now also demonstrated the considerable improvement in predicting SNP-gene pairs by Tejaas (Fig. 2c and Fig. S11, Supplementary Sec. 4.4) using the simulations. All pairs of trans eQTL SNPs and target genes are ranked by their P-values. Each true positive is a correctly predicted SNP-gene pair, each false positive is a wrongly predicted pair. For Tejaas and FR, trans-eQTL SNPs were preselected using a cut-off, while MatrixEQTL does not provide any such preselection. The massive improvement underscores that, to identify target genes, a crucial step is to correctly predict the trans eQTL SNPs.*

*Additionally, we show in Fig. 3f the trans-eQTLs and their target genes for two different tissues, Artery Aorta and LCL. Trans-eQTLs and their target genes for all tissues are available for download (Data Availability). In lines 283 – 310, we provide three examples showing the functional relevance of target genes for three trans-eQTLs.*

2.	The authors claim that the null distribution for the test statistic can be approximated by a normal distribution with an estimated mean and variance for which the authors provide an analytical form. Additionally, they have shown that the analytically estimated mean and variance of the test statistics agree well with that from permutation-based estimates. However, the authors need to additionally evaluate whether the normality assumption is valid or not through comparisons of qq-plot (or otherwise) especially in the extreme tails (~5e-08). Slightest deviations from the normality assumption can result in substantial impact on the output. This is a critical addition, and could change the conclusions significantly if the tails are not well modeled.

*Thank you for the suggestion. First, we have included Fig. S1c. The caption reads:*

*“QQ plot of the empirical null distribution of q_rev for four representative SNPs with minor allele frequencies of 0.1, 0.2, 0.3 and 0.4. On the x-axis, we plot the theoretical quantiles of N(0,1) and on the y-axis, we plot the observed quantiles of (q_rev - \mu_q) / \sigma_q. With a sampling size of 1e7 empirical permutations, the maximum observed quantiles correspond to p-values of 1.2e-8, 1.5e-8, 3.7e-8 and 1.4e-8 respectively for the four SNPs. For all plots in this figure, we used the gene expression of adipose subcutaneous tissue from GTEx.”*

*Additionally, we included Fig. S4. The caption reads:*

*“Normal approximation of permuted q_rev remains valid after KNN correction. Using the gene expression of adipose subcutaneous tissue from GTEx, we applied the KNN correction with K = 30. Here, we show that the empirical null distribution of q_rev follows the normal distribution for four representative SNPs with minor allele frequencies (MAF) of 0.1, 0.2, 0.3 and 0.4 ...”*

3. Questions also arise in the choice to model a non-negative test statistic through normal distribution, which could also lead to incorrect conclusions.

*We argued for the normal distribution of the test statistic in Supplementary Sec 2.6*

I believe the model framework and hypothesis of inference is exactly similar as the SKAT model (aggregating effects across multiple variants). The authors should test a variance-component based score test, which could be computationally efficient and the modelling test statistic as a summation of chi-squares null distribution has been proven to work well in moderate sample sizes and high significance levels (~2.5e-06).

*We thank the reviewer for the valuable comment. We have added a paragraph to the Discussion briefly summarizing the differences between burden test, SKAT and Tejaas. Lines 356 – 363 reads:*

*“The aggregation of weak signals from many covariates in Tejaas is reminiscent of the burden test [54] and the sequence kernel association test (SKAT) [55, 56], which were developed for finding rare genetic variants associated with a trait. Whereas burden and SKAT ask whether to reject the null hypothesis β=0, Tejaas uses Bayesian model comparison of the null model β=0 with the alternative model β≠0 (Eq. (3)) while integrating out the unknown effect strengths β. For γ→0, Tejaas’ test statistic (q_rev ) tends towards the unweighted SKAT statistic (Supplementary Sec. 2.7). However, Tejaas predictions deteriorated for γ → 0 (Fig. S7b).”*

*We have also added Supplementary Sec. 2.7 to explain the differences in more detail:*

4. I don't fully agree with the statement in the Discussion section that "...All trans-eQTLs identified to date, including the meta-analysis on whole blood with 31,684 individuals, were discovered by strong effects on a single, distant gene. Hence, by design, Tejaas might not replicate these existing trans-eQTLs with statistical significance...". The authors claim that their method is designed to aggregate weaker effects to identify trans-eQTLs. In eQTLGen, due to higher power resulting from much higher sample size, the standard trans-mapping is able to capture many weaker effects which Tejaas "might be" able to capture in a smaller sample size as in GTEx. I believe this statement was motivated by the very small overlap of the trans-eQTLs detected via Tejaas and eQTLGen. But since even such small overlap is much more than expected (enriched), this strengthens the method's properties. Furthermore, there are existing papers that aggregate signal to identify trans-eQTLs, including PMC5384037, PMC7444084 and others.

*Many thanks for the helpful remarks. We have updated the Discussion in our main text, lines 371 – 379:*

*“Third, Tejaas was developed to aggregate weak effects across many target genes to detect trans-eQTLs and it may not pick up strong, single SNP-gene associations like standard trans-mapping methods. Therefore, Tejaas and standard trans-mapping methods are complementary and we expect rather low overlaps between trans-eQTLs predicted by these two approaches. However, the weak trans-effects predicted by Tejaas might be detected by standard trans-mapping when using a sufficiently large sample size. This seems to explain the significant fraction of overlap (enrichment p-value≈ 3 × 10 −9, Supplementary Appendix 3) between trans-eQTLs predicted in GTEx whole blood by Tejaas and those predicted in eQTLGen whole blood meta-analysis with 31 684 individuals [5].”*

5.	In Simulations, authors have used a gamma effect size distribution. However, we need to see how well this replicates weaker effects normally seen in trans-eQTLs. The authors should quantify average number of significant variant-gene associations obtained from such effect size distributions to calibrate the expectation from standard trans-mapping. Also, it would be good to get a sense of the heritability of gene-expressions explained by such effects since it is estimated that about 70% of gene-expression heritability is trans-regulated. Does the simulation scheme reflect that?

*To address the choice of parameters in the simulation, we have now included Supplementary Sec. 4.1.3. In Fig. S6a, we compare the effect size distribution in simulations with that of predicted cis-eQTLs and predicted trans-eQTLs using simple regression in GTEx. In Fig. S6b, we show the proportion of expression heritability explained by trans-eQTLs with the different sets of simulation parameters used for Fig. 2. The trans signals are indeed too weak to be predicted by simple regression. The proportion of trans-heritability is also much less than 0.7. Therefore, the simulation parameters represent rather more challenging settings than expected for real data.*

6.	I find the enrichment of the detected trans-eQTLs among GWAS variants rather weak which might be caused by false/noisy signals captured by Tejaas?

*Again a very helpful comment, thank you. The problem is, of course, that we do not know what would be the enrichment of true trans-eQTLs among the GWAS traits. However, we can get an idea of the scale of enrichment to expect by comparing with the enrichment of GTEx cis-eQTLs for the disease categories. Furthermore, the enrichment depends on the chosen p-value cutoff for the GWAS SNPs. We updated Fig. 5b to reflect both of these. The caption now reads:*

*“Enrichment of cis-eQTLs (left panel) predicted by GTEx consortium and enrichment of trans-eQTLs (right panel) predicted by Tejaas for 11 disease categories compiled from 86 GWAS of complex diseases [29]. The enrichment generally increases with decreasing p-value cutoff (x-axis) for the GWAS-associated SNPs. Only those tissues with significant enrichment (p ≤ 0.05) at a cutoff of p = 1 × 10-6 are shown in the plot. While cis-eQTLs are enriched for majority of tissues, enrichment of trans-eQTLs in any disease category is tissue-specific, and the top two tissues with maximum enrichments are noted in the respective panel headers”*

*We also updated the text in the Results section. The signals are indeed noisy, which could be due to statistical noise or false positives. However, the disease-tissue pairs are strongly suggestive of true physiological links.*

Further in Fig 3, the number of trans-eQTLs without cis-effect in several tissues like Blood and Muscle looks rather high. Can the author's comment if that's a real finding or would it be prone to errors.

*We added a paragraph to the Discussion (lines 380 – 397) addressing this point:*

*“Tejaas is to our knowledge the first method whose sensitivity for trans-eQTL discovery does not depend on the presence of a cis effect, because cis genes are masked before reverse regression. The trans eQTLs are therefore unbiased with respect to potential cis effects. We can detect a significant cis effect for about a fifth of the predicted trans eQTLs in most tissues, which is more than expected by chance (p<0.01 for most tissues, Fig. 4b). However, if trans-eQTLs act via diffusible factors as is generally believed, why do not all trans eQTLs have a cis effect? First, some diffusible factors might be as yet unannotated non-coding RNAs. Second, it is likely that we cannot detect the cis effects for a good fraction of trans eQTLs because of low signal-to-noise ratios. Third, cis and trans effects might not occur in the same tissue, and forth, the cis effects might have an influence on cellular or organismal decisions that amplify them enormously. For example, some SNPs might influence the bias in cell differentiation (such as B versus T cells), which impacts cell type composition, others influence the threshold for switching on or off certain pathways such as producing insulin. The consequences of such decisions would strongly manifest themselves in the gene expression levels as trans effects, while the cis effects would only be present in a tiny number of cells that might even reside in a different tissue. For example, the tiny fraction of hematopoietic stem cells in the bone marrow would influence blood cell composition, and beta cells producing insulin in the pancreas would influence gene expression in the liver, muscle, and adipose tissues.”*

7.	The increase in trans-eQTLs after cross-mapping filtering is unusual and should be double checked and explained. No p-values should have changed and only a minority of candidate tests would be filtered, so the multiple hypothesis correction should not be affected dramatically. In response to the review, please provide examples of hits that were filtered by this step, and hits that were brought in by this step.

*The increase in number of trans-eQTLs after cross-mapping filter was indeed a mistake in our analysis: We did not re-estimate the regularizer after cross-mappability filter. This is now clarified in Supplementary Sec. 5.9. The cross-mapping filter removed a significant number of genes (often more than thousands, e.g. ~3500 in average for whole blood) from the expression data, which necessitated re-estimation of the regularizer. Using the wrong regularizer for some tissues led to false positives in our original manuscript, which we have now fixed.*

*Examples of hits that were filtered and brought in by this step are now shown in Fig. S16:*

*“Effect of cross-mappable genes for trans-eQTL discovery. For each tissue, we show the fraction of significant and non-significant SNPs (brought in and filtered out, respectively) after cross-mappability filter with respect to the number of significant SNPs before the filter was applied. (A) Results using optimization of γ before cross-mappability filter (no optimization after cross-mappability filtering). (B) Results using optimization of γ before and after cross-mappability filter.”*

*A summary of the number of lead trans-eQTLs before and after cross-mappability filter are now provided in Table S1.*

8.	Relatedly the authors should provide the significant SNPs and potential explanatory genes for all data sets in a supplementary table.

*We had already uploaded this dataset to wwwuser.gwdg.de/~compbiol/tejaas/2020_03, as mentioned in Data availability, but unfortunately the link was broken. We have now fixed it.*

Comments by Referee 2 (GBIO-D-20-01439)

This manuscript describes a method to detect trans-eQTLs by an L2-regularized multiple regression. Although it looks like an interesting manuscript, this review has some questions and comments on the study.

1.	It seems to me that the methods' limitation of being unable to identify the target genes of a discovered trans-eQTL substantially diminish its practicability. In the genetics community, what we pressingly need is to find out all possible trans-eQTLs of a gene in a given tissue context, but not to merely know whether a SNP is a trans-eQTLs for some genes in some tissues. Once a SNP is a significant trans-eQTLs by the proposed method, one still has to use conventional regression to link the variant to one or more genes. The correlation of genes will always confuse the determination of functionally linked genes of a trans-eQTL.

*Tejaas does predict the target genes, which we did not state clearly enough in the main text. We have now mentioned it in the Introduction, lines 75 – 76:*

*“Predicted trans-eQTLs are ranked by p-value and possible target genes are reported with their estimated FDR.”*

*We now include Benjamini-Hochberg false discovery rates (FDR) for all trans target genes identified with an FDR below 50% (or any threshold specified by --fdrgenethres), for all predicted trans eQTL SNPs with P-value below 5 × 10-8 (or any threshold specified by --psnpthres). This is discussed in Supplementary Sec. 2.9.*

*We have now also demonstrated the considerable improvement in predicting SNP-gene pairs by Tejaas (Fig. 2c and Fig. S11, Supplementary Sec. 4.4) using the simulations. All pairs of trans eQTL SNPs and target genes are ranked by their P-values. Each true positive is a correctly predicted SNP-gene pair, each false positive is a wrongly predicted pair. For Tejaas and FR, trans-eQTL SNPs were preselected using a cut-off, while MatrixEQTL does not provide any such preselection. The massive improvement underscores that, to identify target genes, a crucial step is to correctly predict the trans eQTL SNPs.*

*Additionally, we show in Fig. 3f the trans-eQTLs and their target genes for two different tissues, Artery Aorta and LCL. Trans-eQTLs and their target genes for all tissues are available for download (Data Availability). In lines 283 – 310, we provide three examples showing the functional relevance of target genes for three trans-eQTLs.*

2.	The motivation of the reverse regression is not very convincing. The multiple regression is not good at handling covariates with correlation, and the collinearity may make the model unstable.

*We respectfully disagree. The correlation makes the prediction of target genes (causal covariates) unstable when the covariates are highly correlated, it does not make the prediction of trans eQTLs unstable. The distinction between null model and trans eQTL model is unaffected by the correlation in the covariates. That is the elegance of the reverse regression. In contrast, the sensitivity for trans eQTL discovery by Brynedal et al (AJHG 2018) is indeed negatively affected by the strong correlation.*

3.	A more problematic issue is the "curse of dimensionality". You may have thousands of genes in around 50 tissues, which is a vast dimension. How the L2-regularized is sufficient for this issue in practices is doubtable.

*We used L2-regularization in each tissue individually, not on all 50 tissues together.*

*We showed in rigorous simulations with transparent and realistic assumptions that the L2 regularization works well for trans eQTL discovery. To understand why, one should note first that, while sparsity-encouraging regularizers such as L1 or elastic net are better to identify causal covariates in sparse problems, L2 regularizers often perform similarly well in model comparison (Tejaas' main task) even on sparse problems, particularly when covariates have high measurement errors as in our case. Second, our problem is only moderately sparse. We expect on the order of 100 to 200 target genes per trans eQTL. Assuming that each gene is strongly correlated with 9 other genes on average, we therefore expect on the order of 1000 to 2000 genes whose expression level is somewhat predictive of the trans eQTL minor allele count. In summary, a sparsity-encouraging regularizer might not be better than L2. While we would still prefer to use L1, this choice does not lead to a score whose significance we could efficiently calculate analytically.*

4.	What the Forward Regression does is the analysis of p-values at all genes for a SNP. So both the Forward Regression and reverse regression have similar multiple testing burden.

*We did not mean to say that Reverse (multiple) Regression has a lower multiple testing burden than our CPMA-like Forward (multiple) Regression method. We do claim that it has a lower burden than simple regression approaches such as MatrixEQTL. MatrixEQTL performs 1E8 × 1E4 hypothesis tests, while reverse multiple regression performs 1E8 tests. Hence it reduces the multiple testing burden by a factor 1E4. We have improved the explanation in the main text (lines 70 – 71):*

*“Second, the multiple testing burden is reduced in comparison to single SNP-gene tests because association is tested for all genes at once.”*

5.	In the methods section, "We model x with a univariate normal distribution...", x is just a standardized genotype code and discrete. Maybe it is not suitable to assume it follows the normal distribution.

*To better motivate our modeling assumption, we have added a half-phrase in the Results part (lines 99 – 100) mentioning that this type of modeling assumption is actually quite common in genetics:*

*"Analogous to a common practice of modeling binary GWAS traits using linear instead of logistic regression for ease of computation [19], we model x using linear regression ..."*

*One more crucial point to note here is that the model is only used as a motivation to obtain the test statistic. Once we obtain the test statistic, the null model is calculated explicitly using a permutation of genotype data (and not a normal distribution of x).*

*"All models are wrong, but some models are useful" (George Box). We hope our results demonstrate that the model underlying Tejaas is useful.*

6.	MatrixEQTL and FR were both used to compare with the proposed method in simulation studies, and the proposed method turned out to be best. What is the comparison performed in a real dataset?

*We already compared Tejaas with MatrixEQTL for the GTEx data in our original manuscript (lines 186 – 188 on revised manuscript):*

*“For comparison, the latest analysis by the GTEx consortium on the the same data yielded 142 trans-eQTLs across 49 tissues analyzed at 5% false discovery rate (FDR), of which 41 were observed in testis [4]”*

*We could not compare enrichment analyses with so few predicted trans eQTL SNPs. As we had already written (lines 340 – 345):*

*“So far, most studies have predicted too few trans-eQTLs for such an analysis. Large-scale meta-analysis projects had inherent selection biases which did not allow for enrichment analyses. For example, the meta-analysis of 31684 individuals on whole blood by the eQTLGen consortium [5], which predicted 3853 trans-eQTLs, tested only GWAS-associated SNPs for trans-effects. Consequently, the discovered trans-eQTLs inherited the enrichments of the GWAS-associated SNPs.”*

*FR is worse than MatrixEQTL under all settings of the simulations (Fig. 2), hence we did not apply FR on the GTEx dataset.*

7.	In simulation studies, why the correction methods were different (i.e. For MatrixEQTL and FR, we chose the CCLM correction and for Tejaas, the KNN correction.)?

*This was explained in Fig 2a of our manuscript. MatrixEQTL and FR works best with CCLM correction, Tejaas works best with KNN correction. In lines 149 – 150, we had already written*

*“For FR and MatrixEQTL, CCLM works much better than KNN because we provided it with the known confounders, whereas KNN did not and can not use this”*

*We wanted to ensure the best possible outcome from MatrixEQTL and FR to be compared with Tejaas.*

8.	The authors estimated trans-eQTLs in most of the GTEx tissues. I wonder if others have reported these findings?

*To our knowledge, no other existing method is powerful enough to predict so many trans-eQTLs in the GTEx data. In lines 334 – 335, we had written*

*“To our knowledge, these results represent the first systematic large-scale prediction of trans-eQTLs in the GTEx dataset.”*

I also wonder if these findings can be validated in an independent dataset.

*In our original manuscript, we had addressed this (line 203 – 209 in revised manuscript):*

*“Given the known difficulties to replicate and validate trans-eQTLs and the lack of RNA-Seq datasets with coverage of tissues other than whole blood, we tested the validity of our results by analyzing the enrichment of the predicted trans-eQTLs in functionally annotated genomic regions. One would expect only true eQTLs to be enriched in these regions. The functional enrichment measurements were compared to a set of randomly chosen SNPs from the GTEx genotypes (Supplementary Sec. 5.6). The trans-eQTLs were discovered excluding all genes in the vicinity of that SNP and therefore it is unlikely to observe functional enrichments driven by falsely discovered cis-eQTLs.”*

*We had also reported significant enrichment (p ~ 1e-9) in overlap of our predicted trans-eQTLs in blood tissue with known blood trans-eQTLs (Appendix 3; however it is not a validation because eQTLGen includes GTEx as one of its cohorts).*

*In the only publication predicting trans eQTLs based on their joint effect on many target genes (Brynedal et al, AJHG 2018), no validation of predicted trans eQTLs was performed.*

*We hope the results could be validated when we get access to other RNA-Seq eQTL datasets with multiple tissues, which are common with GTEx (trans-eQTLs as we have shown are tissue-specific).*

9. In Figure 4, there was no picture explanation for Figure 4a.

*Thanks. The label "a" got lost, we added it back in the revised version.*

**Second round of review**

**Reviewer 1**

I thank the authors for taking the time to address my previous comments. They have satisfactorily answered my concerns regarding the theoretical properties of the test statistic, in particular the calibration of the estimated and empirical null distribution. Though their argument of approximating a quadratic form test statistic using a normal distribution is not entirely satisfying, their numerical experiments in the supplements seem to show that this might not be a huge concern from a practical standpoint. However, I still have several comments about the presentation of simulation and the results:

1. The authors show the pAUC for different methods including tejaas when FPR < 10%. This comparison is valid and answers partially my previous question. However, what I could not find an explicit answer to, was how many times in the simulation settings have there been an FPR > 10% or 5%. The authors have shown their results conditional on the FPR being less than 10%, but if the FPR for tejaas is more than 10% more often than the other methods, then it invalidates the broader objective of the comparison. I do not like the simulation results reported as pAUC rather than the more traditional approach of the proportion of times a true null (non-null) association was identified, could both be presented?

2. I still find it potentially concerning that there is very little replication of the results in GTEx Whole Blood in eQTLGen. I don’t agree with the authors’ explanation that tejaas is complementary to standard trans-mapping, and without evidence that this explains the lack of replication, other explanations including simply that there are false positives must be considered – without additional analysis the writeup should explicitly specify that the lack of replication could be caused by false positives in addition to their current explanation.

My own intuition suggests Tejaas would pick up strong as well as weaker signals while standard trans-mapping would pick only the stronger effects. If several of the weaker associations picked up by tejaas in GTEx whole blood were indeed true positives, I would expect a much larger overlap with the trans-eQTLs in eQTLGen (although there was is an enrichment that the authors report).

3. I am not fully satisfied by the authors’ explanation and procedure to discern target genes. As I understand, the authors identify the trans-eQTLs and then use the p-values for association of that SNP across all genes, calculate FDR levels and rank them. However, by doing this, the FDR adjustment would not be well calibrated overall (since the same data is used for the initial identification of SNPs, this is double-dipping) nor will it be comparable to standard trans-mapping. At minimum, the authors should be clear that these FDRs cannot be used as if they are calibrated values.

Minor comments:

1. I like the discussion comparing SKAT/Burden tests to tejaas. In my previous round of comments, what I meant was that the underlying model of SKAT and tejaas are the same (random effects model), but what is unclear to me is why the authors wanted to pursue the Bayesian route of inference (which is perhaps more computationally intensive) as compared to score based approach from the onset.

2. In Figure S1c, I think it is much more important to demonstrate the tail the behavior/calibration of the empirical and approximated null distribution. I would request the authors to go till +/-8 (or lower), especially in the range where the quantiles correspond to lower that (-\mu_q/sd_q) which is beyond the range of the empirical null but in the range for normal approximated null. Can the authors comment in the supplementary, how often we see that in real data (may be eQTLGen or GTEx)?

3. Other than the simulations context, I prefer the authors not use the term predict trans-eQTLs. In the real data they have only identified presence of one or more associations and have not “predicted”.

**Reviewer 2**

**Authors Response**

**Point-by-point responses to the reviewers’ comments:**

Responses to reviewer comments:

Reviewer #1: I thank the authors for taking the time to address my previous comments. They have satisfactorily answered my concerns regarding the theoretical properties of the test statistic, in particular the calibration of the estimated and empirical null distribution. Though their argument of approximating a quadratic form test statistic using a normal distribution is not entirely satisfying, their numerical experiments in the supplements seem to show that this might not be a huge concern from a practical standpoint. However, I still have several comments about the presentation of simulation and the results:

1. The authors show the pAUC for different methods including tejaas when FPR < 10%. This comparison is valid and answers partially my previous question. However, what I could not find an explicit answer to, was how many times in the simulation settings have there been an FPR > 10% or 5%. The authors have shown their results conditional on the FPR being less than 10%, but if the FPR for tejaas is more than 10% more often than the other methods, then it invalidates the broader objective of the comparison. I do not like the simulation results reported as pAUC rather than the more traditional approach of the proportion of times a true null (non-null) association was identified, could both be presented?

*We have now included Fig. S12, which shows the proportion of times a true non-null association was identified.*

*We realized that the partial AUC is not widely used in statistical genetics yet, and we therefore added an explanatory paragraph in the Results / Simulation section.*

*"Ranking performance is often summarized using the area under the ROC curve (AUC), the curve of true positive rate (fraction of true positives with respect to all positives) versus false positive rate (fraction of false positives with respect to all negatives) for all thresholds. However, for prediction tasks where the number of negatives is much larger than the number of positives, as in trans eQTL discovery, most part of the ROC curve corresponds to high FDR (false discovery rate / type I error rate) so high that it is irrelevant. For example if there are 10 times more negatives than positives, at FPR = 0.1 the number of false positives is equal to the total number of positives and hence the FDR is at least 0.5. To alleviate this deficiency, we use the partial area under the ROC curve (pAUC) up to FPR=0.1. An ideal predictor will obtain pAUC=0.1 (Fig. S7 and Supplementary Sec. 4.2)"*

*We also expanded Section 4.2 and included an explanatory Fig. S7.*

2. I still find it potentially concerning that there is very little replication of the results in GTEx Whole Blood in eQTLGen. I don't agree with the authors' explanation that tejaas is complementary to standard trans-mapping, and without evidence that this explains the lack of replication, other explanations including simply that there are false positives must be considered - without additional analysis the writeup should explicitly specify that the lack of replication could be caused by false positives in addition to their current explanation. My own intuition suggests Tejaas would pick up strong as well as weaker signals while standard trans-mapping would pick only the stronger effects. If several of the weaker associations picked up by tejaas in GTEx whole blood were indeed true positives, I would expect a much larger overlap with the trans-eQTLs in eQTLGen (although there is an enrichment that the authors report).

*We have updated the discussion:*

*“Third, Tejaas was developed to aggregate weak effects across many target genes to detect trans-eQTLs and it may not pick up strong, single SNP-gene associations like standard trans-mapping methods. Therefore, Tejaas and standard trans-mapping methods are complementary and we expect rather low overlaps between trans-eQTLs predicted by these two approaches. However, the weak trans-effects predicted by Tejaas might be detected by standard trans-mapping when using a sufficiently large sample size, for example, the eQTLGen whole blood meta-analysis with 31 684 individuals [5]. Only 0.96% of the eQTLGen trans-eQTLs overlap with the putative trans-eQTLs predicted by Tejaas on GTEx data (enrichment p-value ≈ 3×10−9 compared to random overlap; Supplementary Appendix 3). This could in part be due to the complementary nature of the analyses and in part by the prediction of false positive associations.”*

3. I am not fully satisfied by the authors' explanation and procedure to discern target genes. As I

understand, the authors identify the trans-eQTLs and then use the p-values for association of that SNP across all genes, calculate FDR levels and rank them. However, by doing this, the FDR adjustment would not be well calibrated overall (since the same data is used for the initial identification of SNPs, this is double-dipping) nor will it be comparable to standard trans-mapping. At minimum, the authors should be clear that these FDRs cannot be used as if they are calibrated values.

*We thank the reviewer for pointing out this problem with the FDR estimates. We agree that the Benjamini-Hochberg FDR values are not correctly calibrated. We now alert the reader to this issue:*

*"Finally, although we report the Benjamini-Hochberg adjusted p-values for the target genes of the trans-eQTLs, they suffer from two drawbacks: (1) Since we select the candidate trans-eQTL SNPs based on their association with gene expression levels (double dipping), the p-values of the SNP-gene pairs are not uniformly distributed under the null model any more. Therefore, the FDR adjustment can result in too optimistic values. (2) the i.i.d. assumption for the p-values is not correct due to correlation among the gene expression levels, leading to correlated p-values and miscalibrated FDR adjustments. Hence, the adjusted p-values can only serve to rank target genes for any given trans-eQTL, but they are neither directly comparable with standard trans-mapping FDR-adjusted p-values nor between different trans-eQTLs."*

Minor comments:

1. I like the discussion comparing SKAT/Burden tests to tejaas. In my previous round of comments, what I meant was that the underlying model of SKAT and tejaas are the same (random effects model), but what is unclear to me is why the authors wanted to pursue the Bayesian route of inference (which is perhaps more computationally intensive) as compared to score based approach from the onset.

*We added two paragraphs to the end of Supplemental Section 2.7 motivating the development of the statistic in TEJAAS and comparing it with SKAT.*

*"Whereas the SKAT statistic was devised because it was deemed useful to distinguish positives from negatives while being easy to compute, we used the Bayesian approach to derive a test statistic that should be reasonably good in distinguishing positives from negatives. Indeed, despite the similarity with the SKAT statistic, the equivalent TEJAAS kernel contains a correction R for the correlation between covariates, which does not appear in SKAT and related methods. This correction has its origin in the non-null model, for which regression coefficients deviating substantially from 0 are considered. "*

*"Once derived, we strove to make the TEJAAS statistic fast to compute by means of analytically calculating the first and second moments (Appendix 1). Its time complexity is dominated by the inversion of the matrix σ2/γ2 I +YᵀY, which takes O(N G min{G,N}), while p-values are computed in O(N2) (Appendix 1). In comparison, SKAT scores are computed in O(NG), while the computation of the p-values requires the inversion of an N×N matrix, taking time O(N3)."*

2.	In Figure S1c, I think it is much more important to demonstrate the tail the behavior/calibration of the empirical and approximated null distribution. I would request the authors to go till +/-8 (or lower), especially in the range where the quantiles correspond to lower that (-\mu_q/sd_q) which is beyond the range of the empirical null but in the range for normal approximated null. Can the authors comment in the supplementary, how often we see that in real data (may be eQTLGen or GTEx)?

*The empirical shuffling is computationally demanding and we have now performed 1e9 permutations. Figure S1c has been updated with the new empirical distribution. In the Supplementary Sec 2.6, we now write:*

*“With this empirical permutation, we could verify that a p-value up to 1x10-12 is accurately approximated by our normal approximation. In our subsequent analyses of GTEx, we found that 2781 association tests (out of 49 x 8048655 association tests) were beyond the empirically verified range.”*

3.	Other than the simulations context, I prefer the authors not use the term predict trans-eQTLs. In the real data they have only identified presence of one or more associations and have not "predicted".

*We use the word “predict” because we understand it to be weaker than "identifying associations", because a prediction could be true or false (association). We now explain this definition in the introduction:*

*"Note that in the remainder of the manuscript, "predicted trans-eQTLs" refers to both true and false associations identified as trans-eQTL SNPs."*

*We understand that “discovering association” does not necessarily imply “finding true trans-eQTLs”.*
